# Understanding the Pathogenicity of *Burkholderia contaminans*, an Emerging Pathogen in Cystic Fibrosis

**DOI:** 10.1371/journal.pone.0160975

**Published:** 2016-08-11

**Authors:** Jaroslav Nunvar, Lucie Kalferstova, Ruhi A. M. Bloodworth, Michal Kolar, Jose Degrossi, Silvina Lubovich, Silvia T. Cardona, Pavel Drevinek

**Affiliations:** 1 Department of Medical Microbiology, 2nd Faculty of Medicine, Charles University and Motol University Hospital, Prague, Czech Republic; 2 Department of Medical Microbiology & Infectious Disease, University of Manitoba, Winnipeg, Canada; 3 Laboratory of Genomics and Bioinformatics, Institute of Molecular Genetics, Academy of Sciences of the Czech Republic, Prague, Czech Republic; 4 School of Pharmacy and Biochemistry, University of Buenos Aires, Buenos Aires, Argentina; 5 Centro Respiratorio Dr. A. Alvarez, Hospital de Niños Ricardo Gutiérrez, Buenos Aires, Argentina; ENEA Casaccia Research Centre, ITALY

## Abstract

Several bacterial species from the *Burkholderia cepacia* complex (Bcc) are feared opportunistic pathogens that lead to debilitating lung infections with a high risk of developing fatal septicemia in cystic fibrosis (CF) patients. However, the pathogenic potential of other Bcc species is yet unknown. To elucidate clinical relevance of *Burkholderia contaminans*, a species frequently isolated from CF respiratory samples in Ibero-American countries, we aimed to identify its key virulence factors possibly linked with an unfavorable clinical outcome. We performed a genome-wide comparative analysis of two isolates of *B*. *contaminans* ST872 from sputum and blood culture of a female CF patient in Argentina. RNA-seq data showed significant changes in expression for quorum sensing-regulated virulence factors and motility and chemotaxis. Furthermore, we detected expression changes in a recently described low-oxygen-activated (*lxa*) locus which encodes stress-related proteins, and for two clusters responsible for the biosynthesis of antifungal and hemolytic compounds pyrrolnitrin and occidiofungin. Based on phenotypic assays that confirmed changes in motility and in proteolytic, hemolytic and antifungal activities, we were able to distinguish two phenotypes of *B*. *contaminans* that coexisted in the host and entered her bloodstream. Whole genome sequencing revealed that the sputum and bloodstream isolates (each representing a distinct phenotype) differed by over 1,400 mutations as a result of a mismatch repair-deficient hypermutable state of the sputum isolate. The inferred lack of purifying selection against nonsynonymous mutations and the high rate of pseudogenization in the derived isolate indicated limited evolutionary pressure during evolution in the nutrient-rich, stable CF sputum environment. The present study is the first to examine the genomic and transcriptomic differences between longitudinal isolates of *B*. *contaminans*. Detected activity of a number of putative virulence factors implies a genuine pathogenic nature of this novel Bcc species.

## Introduction

Bacteria from the *Burkholderia cepacia* complex (Bcc) cause serious respiratory infections in cystic fibrosis (CF) patients and thus significantly contribute to the reduction of life expectancy in CF [1]. Although the Bcc currently comprises 20 different bacterial species, only three of them have been under the spotlight of CF researchers until recently: *Burkholderia cenocepacia* (former genomovar III), which was recognized as a dominant Bcc species with arguably the highest potential for inter-patient transmission (evident from past large epidemic outbreaks caused by *B*. *cenocepacia* strains ET12, PHDC or ST32 [2]); *Burkholderia multivorans* (former genomovar II), which within the last 15 years replaced *B*. *cenocepacia* as the primary species of Bcc infection in many countries [3]; and *Burkholderia dolosa* (former genomovar VI), which is a far less common Bcc species than the former two but is equally clinically important [4]. The most worrisome feature of the three Bcc species in the CF care is the possibility of developing a fatal septic condition called cepacia syndrome [4–6].

*Burkholderia contaminans* was described taxonomically in 2009 as one of the latest Bcc members [7]. Its history related to the CF lung disease started in the early 2000s, when the species was recovered from respiratory samples from Portuguese and Argentinean patients [8, 9]. At that time, the bacteria were incorrectly or indefinitely classified, sometimes as taxon K, based on the restriction fragment length polymorphism (RFLP) pattern of the *recA* gene. Later, a study aiming to unravel the controversial identification of the *B*. *contaminans* metagenome in sea water (designated as *Burkholderia* SAR-1 at that time) indicated that *B*. *contaminans* was the species epidemiologically linked to the contamination of medical devices and products [10]. Today, *B*. *contaminans* is reported to be the most frequent Bcc species in Spanish [11] and Argentinean CF patients [12].

*B*. *contaminans* infections in CF are often of transient nature [13] and thus can be perceived as respiratory tract colonization rather than true infection. However, some patients develop chronic infection with long-term culture positivity [12, 13]. Furthermore, some of the cases can flare up and result in fatalities. Based on this unfavorable clinical manifestation, *B*. *contaminans* is likely an emerging CF pathogen. The circumstances leading to the establishment of true *B*. *contaminans* infections are unknown.

Recently, we performed a thorough transcriptomic analysis on *B*. *cenocepacia* isolates that were recovered from the bloodstream of CF patients with end-stage disease to identify the bacterial factors possibly associated with cepacia syndrome [14]. In this study, we applied an analogous approach. To gain insights into *B*. *contaminans* evolution during chronic infection, we compared transcriptomes and genomes of *B*. *contaminans* isolates recovered from different stages of CF infection from a single patient.

## Results/Discussion

We analyzed seven *B*. *contaminans* isolates that had been recovered on four separate occasions over the course of 6-year long chronic infection (**Table 1**). The patient died at the age of 10 upon the developing septic state with *B*. *contaminans* detected in her blood culture. All isolates were assigned to the multilocus sequence type (ST) 872. ST872 shares the *recA* gene-ST-71 allele with the predominating *B*. *contaminans* population in Argentina [12].

**Table 1:** *B*. *contaminans* isolates used in the study.

Isolate ID	Collection date	Source	Multilocus sequence type	Analyses performed
FFH2055	25-Jul-2005	Sputum	872	WGS [15], phenotypic testing
i_S	14-Oct-2009	Sputum	872	phenotypic testing
L_S	14-Oct-2009	Sputum	872	phenotypic testing
466_S	5-Jan-2011	Sputum	872	phenotypic testing
467_S	5-Jan-2011	Sputum	872	RNA-Seq, WGS, phenotypic testing
MF16_B	11-Oct-2011	Blood	872	RNA-Seq, WGS, phenotypic testing
MF17_B	11-Oct-2011	Blood	872	phenotypic testing

### Transcriptomic differences between ST872 isolates

Initially, we performed comparative transcriptomics between isolate 467_S from sputum and isolate MF16_B from blood. Due to their different sites of origin and dates of collection, we expected to identify changes in gene expression that would be linked to the increasing severity of the infection. The experimental layout included three growth media (CF sputum, CF serum and control medium) and three replicates per isolate and condition. Among the 7,109 protein-coding genes, we observed a greater than 3-fold change in expression in more than 18% of the genes (1,308) under at least one of the three cultivation conditions assessed (for a complete set of genes, see **S1, S2, S3 and S4 Tables, Table 2**). Altered expression was detected for genes with a well established function in *Burkholderia* virulence as well as for more recently discovered genetic clusters with only a putative role in the Bcc pathogenicity.

**Table 2:** The *lxa* locus, putative regulators and co-regulated genes as derived from *B*. *contaminans* genomic and transcriptomic data.

Gene product	Accession No: *B*. *contaminans* FFH2055	Accession No: *B*. *cenocepacia* J2315	Accession No: *B*. *multivorans* ATCC 17616	Fold change of expression (MF16_B/467_S)		
				Serum	Sputum	BSM
***lxa locus***						
pyruvate, water dikinase PpsA	WR30_RS16490	ortholog absent	BMULJ_05585	14.1	3.0	10.4
esterase / lipase	WR30_RS16495	ortholog absent	BMULJ_05586	16.4	2.1	12.6
hypothetical protein	WR30_RS16500	ortholog absent	BMULJ_05590	48.8	14.9	99.7
hypothetical protein	WR30_RS16505	ortholog absent	BMULJ_05591	18.8	4.0	9.3
hypothetical protein	WR30_RS16510	ortholog absent	BMULJ_05592	58.1	15.2	87.4
hypothetical protein	WR30_RS16515	ortholog absent	BMULJ_05594	149.1	8.7	62.2
nicotinate phosphoribosyltransferase PncB	WR30_RS16520	ortholog absent	BMULJ_05598	139.1	7.0	71.5
sulfonate/nitrate/taurine transport system permease SsuC	WR30_RS16525	ortholog absent	BMULJ_05602	50.6	5.7	37.5
sulfonate/nitrate/taurine transport system ATP-binding protein SsuB	WR30_RS16530	ortholog absent	BMULJ_05603	16.4	2.6	8.1
hypothetical protein	WR30_RS16540	ortholog absent	BMULJ_05604	104.7	12.1	70.5
putative signal transduction protein	WR30_RS16545	ortholog absent	BMULJ_05606	48.8	6.6	20.8
hypothetical protein	WR30_RS16550	ortholog absent	BMULJ_05607	15.2	9.9	22.5
hypothetical protein	WR30_RS16570	BCAM0275A	BMULJ_05608	46.2	10.9	39.7
putative universal stress protein	WR30_RS16575	BCAM0276	BMULJ_05609	40.2	3.3	38.3
protein of unknown function	WR30_RS16580	BCAM0277	BMULJ_05610	40.5	3.8	32.9
putative heat shock protein	WR30_RS16585	BCAM0278	BMULJ_05611	230.7	33.1	328.6
hypothetical protein	WR30_RS16590	BCAM0279	BMULJ_05612	81.6	3.9	50.2
putative phospholipid-binding protein	WR30_RS16595	BCAM0280	BMULJ_05613	101.8	18.4	85.6
hypothetical protein	WR30_RS16600	BCAM0280A	BMULJ_05614	136.2	6.9	48.5
putative sulfate transporter family protein	WR30_RS16605	BCAM0281	BMULJ_05615	67.2	2.8	18.0
hypothetical protein	WR30_RS16610	BCAM0282	BMULJ_05616	4.2	4.1	8.5
putative lysine decarboxylase	WR30_RS16615	BCAM0283	BMULJ_05617	8.7	2.8	22.2
putative cytochrome c	WR30_RS16620	BCAM0284	BMULJ_05618	3.7	1.2	4.2
hypothetical protein	WR30_RS16625	BCAM0285	BMULJ_05619	5.2	2.8	6.5
putative alcohol dehydrogenase	WR30_RS16630	BCAM0286	BMULJ_05620	12.9	3.0	9.2
two-component regulatory system response regulator protein	WR30_RS16645	BCAM0288	BMULJ_05622	1.9	1.8	1.7
putative universal stress protein	WR30_RS16650	BCAM0290	BMULJ_05624	44.3	2.7	26.9
putative universal stress protein	WR30_RS16655	BCAM0291	BMULJ_05625	75.6	9.1	28.4
putative universal stress protein	WR30_RS16660	BCAM0292	BMULJ_05626	51.6	11.6	124.5
putative acetate kinase	WR30_RS16665	BCAM0293	BMULJ_05627	37.3	13.1	30.7
putative universal stress protein	WR30_RS16670	BCAM0294	BMULJ_05628	119.4	22.2	135.3
hypothetical protein	WR30_RS16675	BCAM0295	BMULJ_05629	58.1	23.3	41.9
acetoacetyl-CoA reductase PhbB	WR30_RS16680	BCAM0296	BMULJ_05630	168.9	15.6	82.1
putative poly(3-hydroxyalkanoate) polymerase	WR30_RS16685	BCAM0297	BMULJ_05631	54.2	15.3	49.2
putative phosphate acetyl/butyryl transferase	WR30_RS16690	BCAM0298	BMULJ_05632	132.5	15.9	34.5
putative zinc-binding alcoholdehydrogenase	WR30_RS16695	BCAM0299	BMULJ_05633	108.4	9.5	21.9
metallo-beta-lactamase superfamily protein	WR30_RS16700	BCAM0300	BMULJ_05634	52.0	7.3	25.8
hypothetical protein	WR30_RS16705	ortholog absent	BMULJ_05635	196.7	4.9	33.6
RND efflux system outer membrane lipoprotein	WR30_RS16710	ortholog absent	BMULJ_05636	102.5	5.0	9.8
HlyD family secretion protein	WR30_RS16715	ortholog absent	BMULJ_05637	17.6	4.5	16.8
ABC-type antimicrobial peptide transport system ATPase component	WR30_RS16720	ortholog absent	BMULJ_05638	14.9	3.2	12.0
putative ABC-type antimicrobial peptide transport system permease	WR30_RS16725	ortholog absent	BMULJ_05639	29.2	6.6	31.8
hypothetical protein	WR30_RS16730	BCAM0301	BMULJ_05640	43.7	1.2	5.4
putative ABC transporter protein	WR30_RS16735	BCAM0302	BMULJ_05641	20.4	7.2	16.2
ABC transporter ATP-binding membrane protein	WR30_RS16740	BCAM0303	BMULJ_05642	34.1	4.0	21.4
transporter system transport protein	WR30_RS16745	BCAM0304	BMULJ_05643	51.3	3.8	49.5
hypothetical protein	WR30_RS16755	BCAM0306	BMULJ_05645	16.2	7.8	34.5
hypothetical protein	WR30_RS16760	BCAM0307	BMULJ_05646	148.7	24.0	88.1
hypothetical protein	WR30_RS16765	BCAM0308	BMULJ_05647	89.9	11.6	61.8
putative cell division-related metallo peptidase	WR30_RS16770	BCAM0309	BMULJ_05648	118.6	11.0	63.6
ribonucleotide reductase-like protein	WR30_RS16775	BCAM0310	BMULJ_05649	128.9	1.0	16.9
putative 6-phosphofructokinase	WR30_RS16780	BCAM0311	BMULJ_05650	61.0	3.9	17.5
putative polysaccharide deacetylase	WR30_RS16785	BCAM0312	BMULJ_05651	17.8	4.1	23.9
hypothetical protein	WR30_RS16805	BCAM0313	BMULJ_05652	38.1	3.4	23.1
hypothetical protein	WR30_RS16795	BCAM0314	BMULJ_05653	23.3	5.8	16.8
hypothetical protein	WR30_RS16800	BCAM0315	BMULJ_05654	15.5	3.9	33.6
cytochrome c553	WR30_RS29415	ortholog absent	BMULJ_05656	5.5	1.4	4.3
putative cytochrome b	WR30_RS29420	ortholog absent	BMULJ_05658	3.7	-1.4	4.2
hypothetical protein	WR30_RS16555	BCAM0317	BMULJ_05663	173.6	32.9	102.5
putative cation-transporting ATPase	WR30_RS16560	BCAM0318	BMULJ_05664	26.4	3.1	23.8
putative universal stress protein	WR30_RS16565	BCAM0319	BMULJ_05665	36.3	12.2	43.1
putative cytochrome b561	WR30_RS29470	BCAM0320	ortholog absent	6.4	1.7	17.3
hypothetical protein	WR30_RS29475	BCAM0321	ortholog absent	46.2	-1.1	58.1
two-component regulatory system, response regulator protein	WR30_RS29480	BCAM0322	ortholog absent	7.3	1.3	8.9
two-component regulatory system, sensor kinase protein	WR30_RS29485	BCAM0323	ortholog absent	5.9	1.2	5.8
***Putative regulators of lxa locus***						
CRP family regulatory protein	WR30_RS16635	BCAM0287	BMULJ_05621	7.4	7.2	8.8
CRP family regulatory protein	WR30_RS29560	BCAM0049	BMULJ_03224	6.9	-1.7	2.7
cyclic nucleotide-binding transcriptional regulator	WR30_RS23390	BCAM1483	BMULJ_04241	18.8	9.3	10.9
***Co-regulated genes***						
protein of unknown function (DUF1488)	WR30_RS23375	BCAM1480	ortholog absent	44.3	5.1	20.1
hypothetical protein	WR30_RS23380	BCAM1481	BMULJ_04240	22.2	2.5	12.2
hypothetical protein	WR30_RS23385	BCAM1482	ortholog absent	32.7	3.1	3.6
putative universal stress protein	WR30_RS23455	BCAM1495	BMULJ_04252	38.5	7.3	23.6
hypothetical protein	WR30_RS23460	BCAM1496	BMULJ_04253	3.6	1.5	1.4
alcohol dehydrogenase AdhA	WR30_RS24145	BCAM1570	ortholog absent	106.2	2.4	21.7
TonB-dependent receptor	WR30_RS24150	BCAM1571	BMULJ_04320	3.7	-2.5	3.7

#### Quorum sensing (QS) systems

QS is known to regulate a multitude of functions that are involved in Bcc virulence in various laboratory models (reviewed in [16]). The genes encoding signal molecule synthases as well as virtually all QS-regulated virulence-related functions were expressed at higher levels in MF16_B compared with 467_S. They included the extracellular metalloproteases ZmpA and ZmpB [17, 18], siderophore ornibactin [19], lectins BclA and BclC [20–22], the nematocidal protein AidA [23], Flp-type pili and type III (T3SS) and type VI secretion systems [24] (**S2 Table**). However, the extent of expression differences varied considerably among particular genes and among cultivation conditions, with no obvious trend between the expression levels of QS signaling molecule synthases and the transcription of QS-regulated genes.

Bcc QS systems represent an intricate network of pleiotropic, mutually agonistic or antagonistic and often redundant regulators [16, 25], which together leads to a complex output response. A study that examined changes in AHL production by a large Bcc collection during the course of the CF infection found QS signaling to be functionally preserved [26]. Comparison of the gene expression between two *B*. *cenocepacia* isolates from different stages of the CF pulmonary infection revealed both upregulation (ornibactin) and downregulation (secretion systems, extracellular proteases) of QS-dependent functions [27]. Our recent study, similar in design, but involving a bloodstream *B*. *cenocepacia* isolate, demonstrated a significant increase in expression of the entire T3SS cluster [14], a finding consistent with T3SS expression in MF16_B.

#### Motility and chemotaxis

Over 70 genes encoding proteins with a motility-related function and spanning all previously described motility clusters [24] exhibited increased expression in the blood isolate MF16_B (**S3 Table**). This was in marked contrast with our previous observation on *B*. *cenocepacia* strain ST32 where all tested blood isolates (obtained at the time of cepacia syndrome) showed a uniform loss of motility or rapid decrease in motility as measured by swimming activity of the isolates [14]. Another study on a wide range of clinical isolates that belonged to various Bcc species found no apparent association between changes in motility and the clinical outcome of the Bcc infection [28].

#### Biosynthesis of antifungal compounds

Two gene clusters responsible for the biosynthesis of antifungal compounds showed significantly higher expression in MF16_B than in 467_S (**S4 Table**). One of them, pyrrolnitrin, which is produced by some Bcc and several other Gram-negative bacteria, inhibits growth of a wide range of fungi and of some Gram-positive bacteria [29]. Interestingly, a study by Schmidt *et al*. [30] noted the absence of the pyrrolnitrin biosynthetic operon in all of their examined strains of *B*. *contaminans*. However, in our studied *B*. *contaminans* strain ST872, all four genes of the pyrrolnitrin biosynthetic pathway were present, and these genes were also found in every other *B*. *contaminans* sequenced up to now. Pyrrolnitrin production depends entirely on QS [30]; the detected expression changes are thus in accordance with those observed for other QS-regulated functions whose expression was upregulated in the bloodstream isolate MF16_B.

Another antifungal compound, occidiofungin, was first described in *B*. *contaminans* MS14 [31], displaying its activity against a wide range of fungi [32]. The biosynthetic cluster, consisting of 18 genes [33], is *cis-*regulated by two regulators [34]. This cluster is absent in the genomes of some Bcc species, including *B*. *cenocepacia* and *B*. *multivorans* [35]. The occidiofungin biosynthetic cluster, including genes encoding both regulators (AmbR1 and AmbR2), showed increased expression in MF16_B, most markedly in sputum medium (**S4 Table**). Similar to pyrrolnitrin, occidiofungin production was found to be positively regulated by QS [36].

#### *lxa* locus

In 2013, Sass *et al*. described a gene cluster in *B*. *cenocepacia* strain J2315 (BCAM0275A - BCAM0323) whose expression was induced specifically under low oxygen concentration (low-oxygen-activated or *lxa* locus) [37]. The locus exhibits an irregular pattern of presence in Bcc bacteria, e.g., being absent in some strains of *B*. *cenocepacia* or *B*. *multivorans* while being present in the others [37]. Notably, the *lxa* locus (as delineated in *B*. *cenocepacia* J2315) can be found in genomes of all sequenced strains of *B*. *contaminans*, either as a complete set of genes (i.e., *B*. *contaminans* FFH2055 or *B*. *contaminans* MS14; see Table 2) or truncated (missing approximately one quarter of genes from the 3’ end of the locus, i.e., *B*. *contaminans* LMG 23361), whereas it is absent in *Burkholderia lata* strain 383, the closest relative of *B*. *contaminans* [37]. We detected a rapid increase in expression of the entire cluster in MF16_B vs. 467_S. The expression was most pronounced when the bacteria were cultivated in serum and a control medium (**Table 2**). Importantly, all three genes that were predicted to regulate *lxa* expression (BCAM0049, BCAM0287 and BCAM1483) [37] also displayed increased expression in MF16_B. Moreover, several genes neighboring the *lxa* cluster (as delineated in *B*. *cenocepacia* J2315 annotation) exhibited a similar pattern of differential expression. These genes were homologous to *lxa*-adjacent genes in the annotated genome of *B*. *multivorans* ATCC17616 (**Table 2**), suggesting that additional genes form extended, strain-specific *lxa* clusters. Although low-oxygen level was the only condition capable of inducing *lxa* transcription in the study of Sass *et al*. [37], we detected expression of the entire locus under normal aerobic conditions in *B*. *contaminans*. We believe that there is another eminent functional significance of this enigmatic part of multiple Bcc genomes.

### Phenotypic differences between *B*. *contaminans* ST872 isolates

The transcriptomic differences between *B*. *contaminans* isolates 467_S and MF16_B allowed predicting changes in their phenotypic features. To verify the transcriptomic results, we assessed four phenotypes: swimming motility (a proxy of flagellar gene activity), proteolysis (*zmpA* and *zmpB* genes), antifungal activity (pyrrolnitrin and occidiofungin biosynthetic genes) and hemolysis. We assumed that occidiofungin had hemolytic properties because molecules synthesized by pathways highly homologous to that of occidiofungin were tested hemolytic in *B*. *vietnamiensis* [35] and *B*. *ambifaria* [38].

**Fig 1:**
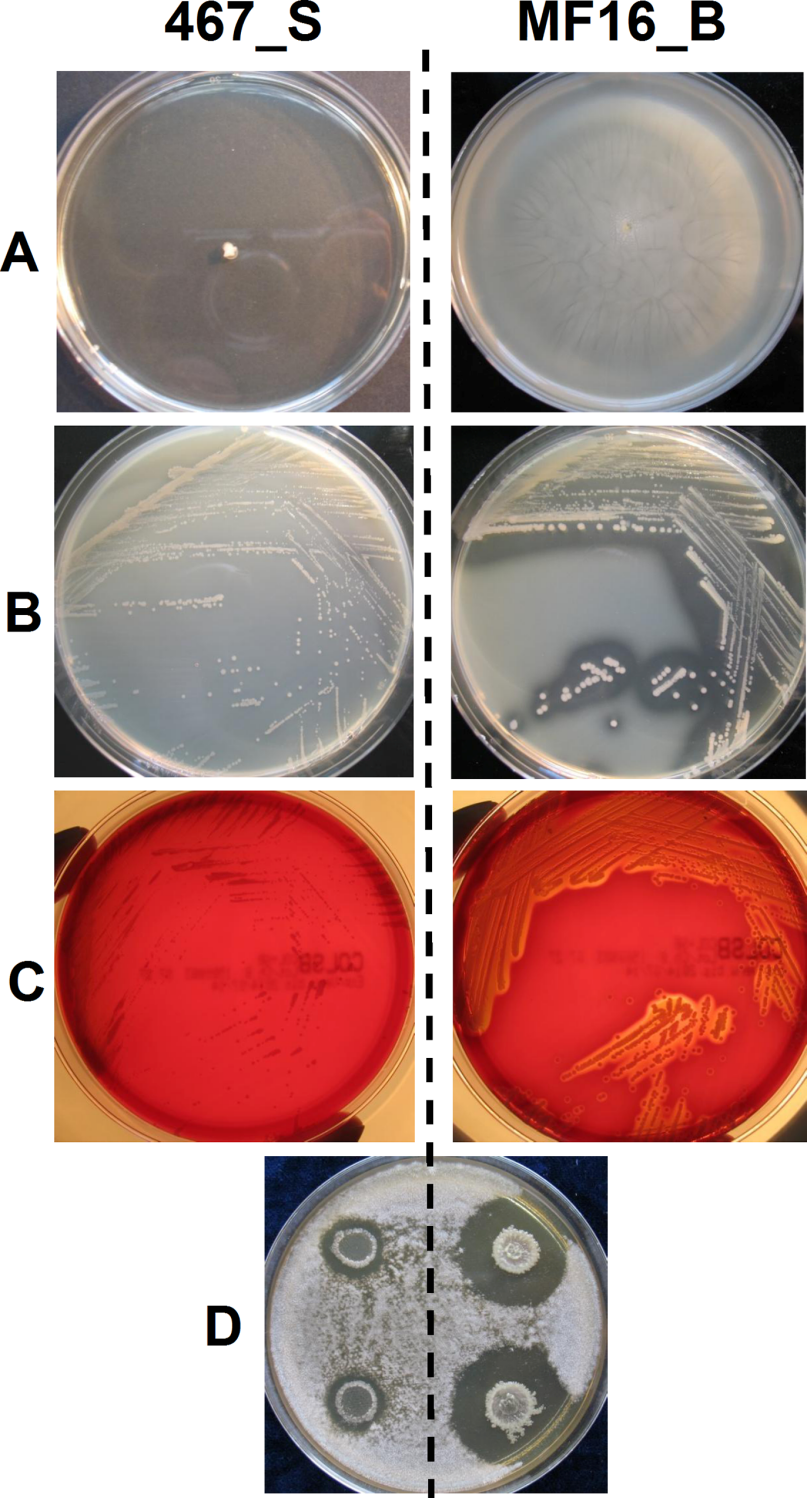
Agar plate phenotypic assays. A) swimming motility, B) proteolysis, C) hemolysis and D) antifungal activity against *Geotrichum candidum*. *Left*–isolate 467_S, *right–*isolate MF16_B.

The phenotypic differences that had been revealed in agar plate assays were in agreement with changes observed at the transcriptional level (**Fig 1**): isolate 467_S was nonmotile and did not show any proteolytic or hemolytic activity after 48 hours of incubation; isolate MF16_B exhibited high motility, proteolysis and hemolysis. Antagonism against the fungus *Geotrichum candidum* was very pronounced in MF16_B, not in 467_S. Although 467_S exhibited slower growth on all agar plates, the differences were equally apparent after prolonged incubation, which gave rise to colonies of 467_S of similar size to that of faster-growing MF16_B (data not shown).

These phenotypic tests were used to screen additional five *B*. *contaminans* isolates that had been collected from the patient at various time points. Strikingly, this simple screening indicated coexistence of two distinct phenotypic groups in the subject’s lung as well as in blood, termed group A (represented by MF16_B) and group B (represented by 467_S) (**Table 3**). It is noteworthy that the phenotype of the blood isolate MF16_B matched the sputum isolate FFH2055 (with the exception of swimming motility), which was retrieved six years earlier. The sputum isolate 467_S had its phenotypic counterpart, isolate MF17, which was obtained from the same blood culture as the MF16_B.

**Table 3:** Phenotypic properties of *B*. *contaminans* ST872 isolates.

Isolate ID	Swimming	Hemolysis	Proteolysis	Activity against *G*. *candidum*	Mutation frequency^§^ (x 10^−8^)	Phenotypic class
FFH2055	-	+++	+++	+++	11	A
i_S	++	-	-	-	1,560	B
L_S	+	-	+	-	5,300	B
466_S	-	-	-	-	2,400	B
467_S	-	-	-	-	1,200	B
MF16_B	+++	+++	+++	+++	9	A
MF17_B	-	-	-	-	1,250	B

^§^ Median frequency of mutations towards rifampicin resistance from three independent experiments

### Genomic differences between *B*. *contaminans* isolates

Genome sequencing of 467_S and MF16_B was performed (Illumina, 2 x 150 bp paired-end sequencing) in order to identify the extent of genetic differences between the phenotypic groups A and B and to complement information from their transcriptomes. Genomic sequences were compared with the reference genome of *B*. *contaminans* FFH2055, the initial isolate obtained from the studied patient and sequenced earlier in another study (8 contigs, PacBio sequencing) [15]. Of note, the reference genome was 8.21 Mb in size, encoded 7,109 proteins, and had a GC content of 66.40%.

When the sequencing reads covering MF16_B genome were mapped to the reference assembly, 44 variants were detected between both genomes. These were exclusively single-nucleotide insertions at tandem poly-GC repeats in MF16_B, indicating possible sequencing bias. To resolve this, we remapped Illumina sequencing reads for FFH2055 on the PacBio-assembled reference of the same isolate (**S5 Table**). This approach revealed that the called differences between FFH2055 and MF16_B genomes come from different sequencing technologies. The omission of nucleotides on poly-GC runs is in line with other observations for PacBio assemblies [39]. After this correction, the genomic sequences of isolates MF16_B and FFH2055 proved to be identical.

Contrary to genome uniformity observed within the phenotypic group A, a substantial number of genetic differences were detected when groups A and B were compared between each other. In total, 1,433 mutations separated the isolates 467_S and MF16_B, representing 0.017% of the total genome size and affecting 1,002 genes (14%) (**Table 4, S6 Table**).

**Table 4:** Summary of the genetic differences between *B*. *contaminans* isolates 467_S and MF16_B.

Type of mutations	Particular mutations^§^ (class: number)					Total number
single-nucleotide substitutions	AT→GC: 855	GC→AT: 327	transversions (all types): 43			1,225
insertions (tandem repeats)	+1 GC: 105	+1 AT: 6	+ two or more nucleotides: 39			150
deletions (tandem repeats)	-1 GC: 15	-1 AT: 12	- two or more nucleotides: 19			46
other indel mutations						12
Total						1,433
intragenic mutations	synonymous: 230	missense: 796	nonsense: 13	frameshifts: 94	other: 12	1,145
intergenic mutations						288
Total						1,433

^**§**^ Mutations in 467_S with respect to the genomic sequence of MF16_B

**Fig 2:**
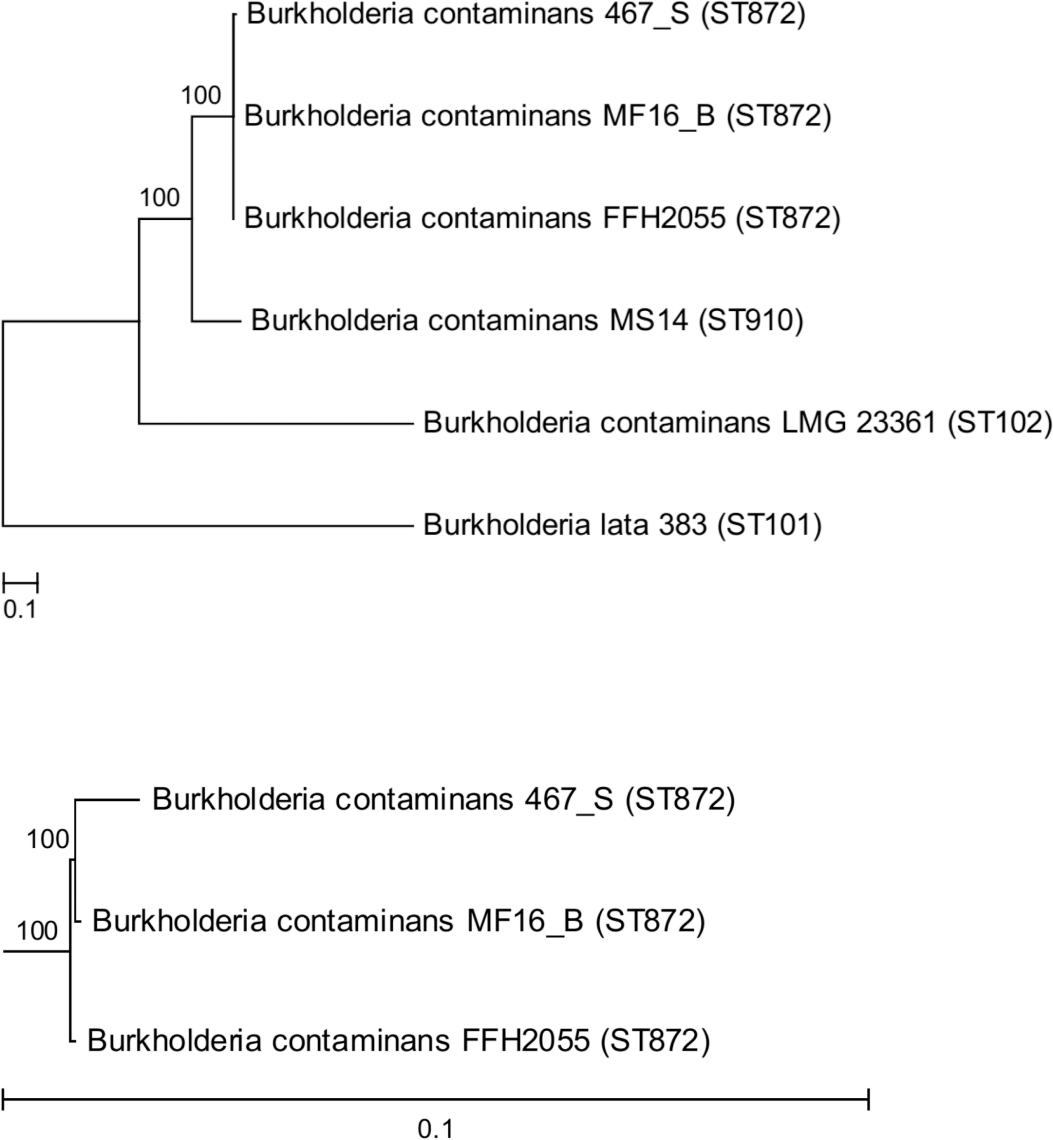
Genetic relationship of sequenced *B*. *contaminans*. The overall tree (*top*) depicts whole-genome phylogeny inferred from SNPs. Multilocus sequence types of the isolates are denoted in parentheses. *Bottom*–the subtree containing only ST872 isolates.

Over 200 indel mutations were detected among the two ST872 genomes representing the two phenotypic groups. Most indels occurred as single-nucleotide insertions or deletions at tandem repeats with a strong bias towards GC repeats. The indel mutations caused 94 frameshifts in coding sequences (**S6 Table**). All affected genes coded for full-length proteins in the reference genome FFH2055 (and in MF16_B), whereas frameshift mutations resulted in their pseudogenization in 467_S. This suggests that isolates from phenotypic group A represent a state ancestral to the derived isolate 467_S, regardless the opposite chronology of collection of respective clinical samples. The notion about the evolutionary order of 467_S vs. MF16_B and FFH2055 was further supported by the basal position of MF16_B (and its genotypically identical early isolate FFH2055) in the phylogenetic tree of *B*. *contaminans* ST872 (**Fig 2**), where isolate 467_S appeared as their genomic derivative. Therefore, the longitudinal position of MF16_B, which was isolated from blood culture, indicates that it might have been re-introduced into the patient from an unknown source (such as some disinfectants, cosmetic products, ultrasound gels or stopcocks for i.v. administration that were all found contaminated with *B*. *contaminans* in different Argentinian hospitals in the past; JD unpublished data). It is unlikely for a bacterium to remain genetically unchanged for 6 years of chronic infection; a study on *B*. *dolosa* reported average genomic substitution rate of approximately 2 SNPs per year of infection [40]. Nevertheless, we cannot definitely rule out an alternative that some members of the clonal phenotypic group A have persisted genetically unchanged, probably in a dormant state intracellularly. The coexistence of diverse lineages as a result of independent transfer of multiple clones into the patient’s bloodstream has been documented in *B*. *dolosa* chronic infection [41].

80% of the detected mutations in 467_S were present in protein-coding genomic regions that occupied 85% of the genome. Among the intragenic single-base substitution mutations, only 230 (22%) were synonymous. This proportion was even lower than a value of 27.8%, which was calculated by Dillon *et al*. as the theoretical probability of synonymous coding substitutions in *B*. *cenocepacia* HI2424 [42]. Taken together with the high number of frameshift mutations, this proportion of synonymous/nonsynonymous mutations indicated absence of strong selection against mutations that would affect a protein function. We can speculate that most of the gene-inactivating mutations (such as frameshifts, nonsense and missense mutations) are tolerable by bacterial cells because they do not disturb vital processes needed for survival of bacteria in CF sputum. A recent study demonstrated that less than 500 genes were essential for *P*. *aeruginosa* growth in CF sputum [43], which is a complex environment very rich in organic nutrients that can be utilized by bacteria. Furthermore, it is known that mutations that negatively affect nutrient uptake or their utilization in a CF sputum are not eliminated by natural selection as rapidly as in more selective environments, as manifested by the frequently observed auxotrophy of Bcc and *P*. *aeruginosa* CF isolates [44, 45].

Regarding the differences in transcriptomes of the two lineages, we found no mutations in genes encoding signal molecule synthases or in their upstream sequences, implicating physiological rather than genetically-based causes of QS expression differences between MF16_B and 467_S. A considerable number of missense and frameshift mutations were identified within motility and chemotaxis genes (7 and 4, respectively–**S3 Table**). In combination with the very low expression of motility genes and a lack of swimming motility in 467_S and other isolates from phenotypic group B, it indicates that these genes are dispensable during long-term *B*. *contaminans* persistence in CF sputum. Decreased production of occidiofungin in isolate 467_S can be, in addition to its diminished transcription, explained by frameshift mutations in two biosynthetic genes (**S4 Table**).

### Hypermutability in ST872

The spectrum of mutations that distinguished 467_S from MF16_B demonstrated a highly uneven distribution in particular mutation types (**Table 4**). Notably, a large excess (approximately 30-fold) of transitions compared with transversion nucleotide substitutions was observed. AT→GC mutations prevailed among transitions and accounted for 70% of all nucleotide substitutions. This pattern of mutations was in perfect accordance with the values obtained in a mutation-accumulation experiment with mismatch repair (MMR)-deficient *Escherichia coli* [46], which is believed to provide the best estimation of the spontaneous mutation rate in bacteria [47]. Therefore, we looked closely at mutations in the genes encoding MMR enzymes, *mutS* and *mutL*. Indeed, one amino acid change was identified in each of the translated proteins in 467_S isolate: 628N→D (1882A→G) in MutS and 317S→P (949A→G) in MutL (**S6 Table**). Both mutations were localized in conserved domains of the MMR proteins and may have therefore abolished their proper function in DNA repair. Notably, both mutations were unique for 467_S and have not been detected in the study that examined many hypermutable Argentinean *B*. *contaminans* isolates [48]. Additional analysis of homologous protein sequences of other Bcc clearly showed that corresponding amino acids in isolates FFH2055 and MF16_B represented wild-type state (not shown). These findings suggest that both mutations in MutS and MutL detected in isolate 467_S have arisen during the evolution of ST872 within the patient´s lungs.

Calculation of the mutation frequency confirmed that isolate 467_S, which carried both MMR mutations, was a hypermutator. The mutation frequency of this isolate was over two orders of magnitude higher than that of MF16_B. FFH2055, the first isolate from the patient, exhibited a normal mutation frequency. All other ST872 isolates, collected after FFH2055 and belonging to the phenotypic group B, were hypermutable (**Table 3**). Thus, the mutator phenotype likely arose early after patient´s colonization and has persisted since then. We assume that during the chronic infection, mutations that escaped correction by MMR have accumulated. The emergence of hypermutators during evolution in CF lungs is a widespread phenomenon that was reported in *P*. *aeruginosa* [49], *Staphylococcus aureus* and *Haemophilus influenzae* [50, 51]. Recently, Martina *et al*. reported a high prevalence of hypermutable *B*. *contaminans* among Argentinean CF patients [48]. Our findings are the first to demonstrate the effect of MMR deficiency on the genomic evolution of *B*. *contaminans* during infection in CF hosts.

## Conclusions

The presented work is the first study that focuses on the genomic and transcriptomic changes that underlie the progression of chronic *B*. *contaminans* infection in CF. Unlike other Bcc species, only little attention has been paid to the mechanisms of pathogenicity of *B*. *contaminans*. This lack of information is worrisome in light of increasing proportion and even predominance of *B*. *contaminans* infections in Argentina [12], Spain [11] and other Ibero-American countries [8], indicating a large and characteristic geographic distribution of considerable concern.

Our results demonstrate that *B*. *contaminans* sequential isolates can hold high genomic diversity despite belonging to the same ST type. This diversity results in differential gene expression patterns, which in turn modify bacterial virulence. We demonstrate the profound effect of DNA repair deficiency on the evolution of the *B*. *contaminans* genome in the stable environment of CF sputum. Observed mutational and transcriptional inactivation of Bcc virulence factors might help to unravel the mechanisms that drive the adaptation of this primarily environmental bacterium to the CF lung during chronic infection.

## Materials and Methods

### Bacterial strains

Seven isolates of *B*. *contaminans* ST872 were obtained from the research *Burkholderia* strain collection of the University of Buenos Aires, Argentina. All isolates originated from a female CF patient (homozygous for the F508del mutation; born in January 2001) who attended Hospital de Niños Ricardo Gutierrez (Buenos Aires) and provided clinical specimens for microbiological investigation as part of her standard CF care. *B*. *contaminans* was cultivated from the patient´s sputum for the first time in July 2005; the lung remained infected since that time. *S*. *aureus* was isolated for the first time in January 2002 (episodes of pulmonary exacerbations were treated with amoxicilin-clavulanic acid); chronic infection with *P*. *aeruginosa* was first observed in March 2007 (inhaled tobramycin was administered in on/off cycles). Between 2008 and 2009, pulmonary function began to decrease (forced expiratory volume in 1 second [FEV1] 45–48% predicted). The patient started to be on frequent courses of outpatient anti-infective therapy (oral minocycline or trimethoprim-sulfamethoxazole), and was also admitted at the hospital every three months for i.v. antibiotic treatment, combining two or more of the following drugs: piperacillin-tazobactam, ceftazidime, amikacin, colistin and trimethoprim-sulfamethoxazole. The patient died in the intensive care unit in October 2011. The death was attributed to *B*. *contaminans* septicemia.

### Bacterial cultivation and RNA extraction

All bacterial cultures were incubated at 37°C with shaking at 230 rpm in 50 ml Falcon conical centrifugation tubes. The bacteria were cultivated in synthetic basal salts medium (BSM) containing 14.3 mM glucose and 0.05% casamino acids [52], CF serum (pooled serum samples from 5 CF patients in various stages of infection, heat-inactivated at 56°C for 30 minutes) and CF sputum (pooled sputum samples from 5 CF patients of various stages of infection, diluted with BSM to a final concentration of 10% w/vol). We used CF serum and sputum samples that were collected and archived for the purpose of this study at the Motol University Hospital, Prague.

Starter cultures were grown overnight in Luria-Bertani broth and diluted to an OD_600_ of 0.5. The bacteria were harvested by centrifugation and resuspended in the same volume of BSM, serum or sputum. In total, 4 ml of BSM and serum and 6.25 ml of sputum were used. The cultures were incubated for 270 minutes (37°C, 230 rpm) into the mid-log growth phase. Then, the cultures were immediately snap-cooled in liquid nitrogen and centrifuged (5 minutes, 160 x *g*, 4°C). The pellets were resuspended in 1 ml of Trizol (Ambion), vortexed thoroughly and incubated for 5 minutes at room temperature. Chloroform (0.2 ml) was added; the samples were shaken for 30 seconds and incubated for 3 minutes at room temperature. The samples were centrifuged (15 minutes, 160 x *g*, 4°C), and the upper water phase was transferred to 0.5 ml of ice-cold 70% ethanol. The RNA samples were further processed using the RiboPure-Bacteria kit (Ambion), and the MICROBExpress kit (Invitrogen) was subsequently used to remove rRNA. The quality of the mRNA was assessed using Total RNA Nano chips on a Bioanalyzer 2100 (Agilent).

The complete procedure (bacterial cultivation, RNA extraction) was repeated three times to obtain three biological replicates per condition.

### Whole genome sequencing and comparative genomics

To extract the genomic DNA, overnight cultures were grown in LB broth and DNA was extracted from collected cells using the DNAeasy kit (Qiagen). The genomic DNA was converted into a sequencing-ready library using the Nextera XT DNA sample Preparation Kit (Illumina). Illumina sequencing was performed in a MiSeq personal sequencing system with TrueSeq^TM^ sequencing by synthesis (SBS) reversible terminator chemistry at the Next Generation Sequencing Core Platform at the Manitoba Institute for Children’s Health (MICH). Data output was assembled by Velvet [53]. Sequencing reads were deposited at the ArrayExpress Archive of Functional Genomics Data (http://www.ebi.ac.uk/arrayexpress/) under accession number E-MTAB-4649.

Sequencing reads were subsequently mapped onto *B*. *contaminans* FFH2055, a reference genome of the multilocus sequence type ST872 [15]. Read mapping was performed with Geneious software 7.1.9 [54] using the Geneious mapper, with a maximum allowed mismatches set to 5%. Variant calling was performed (minimum coverage: 5 reads; minimum variant frequency: 0.75), and the effects of the mutations on the translated proteins were determined.

Complete and draft bacterial genomes for comparison were downloaded from GenBank [55]. A phylogenetic tree was inferred from single nucleotide polymorphisms (SNPs) among the compared genomes using CSI Phylogeny 1.1 [56]. The phylogram was visualized from the tree file using MEGA6 [57].

### RNA-Seq and comparative transcriptomics

RNA samples were processed at the vertis Biotechnologie AG (Freising, Germany) service laboratory for cDNA preparation, library preparation (TruSeq, Illumina) and high-throughput sequencing (Illumina MiSeq, 100 bp reads). Raw RNA-Seq sequencing data were deposited at the ArrayExpress Archive of Functional Genomics Data (http://www.ebi.ac.uk/arrayexpress/) under accession number E-MTAB-4645.

The read sequences were trimmed of low-quality portions and Illumina-specific adapters using Trimmomatic v 0.32 [58] with the following parameters: ILLUMINACLIP:./adapter.fa:2:30:8, LEADING:13, TRAILING:13, SLIDINGWINDOW:4:19, MINLEN:40, and HEADCROP:10. Any reads matching rRNA or tRNA sequences were removed using SortMeRna v 1.99beta [59] utilizing rRNA sequences from the SILVA [60] and Rfam [61] databases as well as rRNA and tRNA sequences from genome assembly of the reference *B*. *contaminans* genome. At each preprocessing step, the technical quality of the reads was assessed with FastQC v0.10.1 (www.bioinformatics.babraham.ac.uk/projects/fastqc/). Subsequently, the reads were mapped to the reference genome (this study). Mapping was performed using GSNAP version 2014-02-28 [62] with the default parameters.

Overlaps of the mapped reads with annotated genes were counted within the Bioconductor environment [63] using the packages GenomicFeatures and GenomicAlignments [64]. Generally, 60% of the raw reads mapped to the reference genome, which is typically 5.5 million mapped reads per sample (range 3.1–8.9, median 5.5 million reads). Quality control for the count data was performed utilizing principal component analysis (PCA), hierarchical clustering, and correlation analysis. All samples were considered adequate for further analyses. The count data were fitted with a generalized linear model (~ source + medium), and differences in the expression intensity between *B*. *contaminans* isolates 467_S and MF16_B in different cultivation media (serum, sputum, and BSM) were evaluated using DESeq2 [65]. Differentially expressed genes were selected with a 3-fold change and a *p*-value of less than 0.05 as the default cutoff. This choice was made due to the high fluctuation of levels of expression among replicates and to select the genes with most pronounced expression changes between the lung and bloodstream isolates.

The nucleotide sequences of differentially expressed genes were subjected to BLAST search [66] against complete *Burkholderia* genomes. Homologous genes from the first sequenced and best-studied *B*. *cenocepacia* strain J2315 [24] were assigned when possible. In cases where no homologs were identified in *B*. *cenocepacia* J2315, homologs from other completely sequenced *Burkholderia* strains with the highest similarity were chosen. Predicted functions were assigned to genes according to the *Burkholderia* Genome Database [67].

### Phenotypic assays

Hemolysis was determined on Columbia sheep blood agar plates (Oxoid) after 48 hours of incubation at 37°C. Protease production was determined on D-BHI agar plates [68] after 48 hours of incubation at 37°C. Production of antifungal substances was determined by the modified method of Chen *et al*. [69]. Five microliters of *B*. *contaminans* suspension (OD_600_ = 1) were spot-inoculated on a malt extract agar plate (Oxoid) and incubated at 37°C for 48 hours. The indicator fungus *Geotrichum candidum* was then replica-plated from a completely covered agar plate (spread-inoculated and incubated for 3 days at room temperature) onto the *B*. *contaminans* plate. The inhibition zones were recorded after 24 hours of incubation at 30°C. Swimming motility was determined on Luria-Bertani (LB) plates containing 0.3% (w/v) Bacto agar (Sigma-Aldrich). The plates were inoculated by injecting 2 μl of bacteria in LB broth (OD_600_ = 1) under the surface of the center of the soft agar plate. The swimming zones were recorded after 48 hours of incubation at 37°C. The mutation frequency was determined as described in [48] with modifications. One microliter of an overnight culture grown from a single colony was diluted in 1 ml of LB broth. One microliter of the diluted culture was then inoculated into 5 ml of LB broth. The cultures were incubated at 37°C with shaking for 24 hours. The cell concentration was then determined by serial dilution of the cultures, followed by plating 100 μl aliquots in parallel onto LB agar and LB agar + rifampicin (100 μg/ml). Colonies were counted after incubation at 37°C for 48 hours, and the mutation frequency was calculated as the fraction of rifampicin-resistant cells in the population. The procedure was repeated three times per isolate.

### Ethics statement

Sputum and serum samples used for bacterial cultivation were taken with the written informed consent of the CF adult subjects. The study was approved by the Ethics Committee for Multi-centric clinical trials of the University Hospital Motol, Prague on September 22, 2010, No.4.2.6.

## Supporting Information

S1 Table Genes exhibiting differential expression between *B*. *contaminans* isolates 467_S and MF16_B (> 3-fold change, *p* < 0.05), not included in Table 2, S2, S3 and S4 Tables. (DOCX)

S2 Table QS regulators and QS-regulated virulence genes with differential expression between *B*. *contaminans* isolates 467_S and MF16_B (> 3-fold change, *p* < 0.05). (DOCX)

S3 Table Motility and chemotaxis genes exhibiting differential expression between *B*. *contaminans* isolates 467_S and MF16_B (> 3-fold change, *p* < 0.05). (DOCX)

S4 Table Antifungal compound biosynthetic clusters with differential expression between *B*. *contaminans* isolates 467_S and MF16_B (> 3-fold change, *p* < 0.05). (DOCX)

S5 Table Variants detected by mapping Illumina sequencing reads (isolates MF16_B and FFH2055) on PacBio reference assembly of FFH2055 (contigs NZ_LASC01000001.1 to NZ_LASC01000008.1). Nucleotide sequence and reading frame of affected genes was assessed by comparison with homologues in *B*. *contaminans* MS14 genome.
(XLSX)

S6 Table List of the genomic differences between *B*. *contaminans* ST872 isolates 467_S and MF16_B. Differences were detected by mapping Illumina reads of 467_S on the FFH2055 reference assembly and subtracting variants present also in MF16_B. The mutations are denoted in the direction MF16_B → 467_S.
(XLSX)
