# Supplementary material for: Understanding the Pathogenicity of Burkholderia contaminans, an Emerging Pathogen in Cystic Fibrosis
Source: PLoS One. 2016 Aug 11;11(8):e0160975. doi: 10.1371/journal.pone.0160975 (PMC4981469; doi:10.1371/journal.pone.0160975)
Supplement: S1 Table — (DOCX) [file pone.0160975.s001.docx]

| **Gene product** | **Accession No: *B. contaminans* FFH2055** | **Accession No: *B. cenocepacia* J2315 or other Bcc** | **Fold change of expression (MF16_B/467_S)** | | |
| --- | --- | --- | --- | --- | --- |
|  |  |  | **SERUM** | **SPUTUM** | **BSM** |
| response regulator receiver modulated diguanylate cyclase/phosphodiesterase | WR30_RS18470 | Bamb_1597 | 2,0 | 3,4 | 3,3 |
| amino acid adenylation protein | WR30_RS32645 | Bamb_5388 | 2,7 | 4,2 | 2,5 |
| filamentous hemagglutinin family outer membrane protein | WR30_RS32920 | Bamb_5443 | 3,6 | 10,0 | 47,5 |
| Polypeptide-transport-associated domain protein, ShlB-type | WR30_RS32925 | Bamb_5444 | 4,3 | 17,5 | 39,4 |
| PpiC-type peptidyl-prolyl cis-trans isomerase | WR30_RS32930 | Bamb_5445 | 6,7 | 24,8 | 54,6 |
| adhesin HecA family | WR30_RS32935 | Bamb_5446 | 5,6 | 18,6 | 18,8 |
| hypothetical protein | WR30_RS32955 | Bamb_5450 | 4,5 | 6,4 | 17,1 |
| hypothetical protein | WR30_RS32960 | Bamb_5451 | 1,8 | 3,3 | 7,2 |
| ATPase domain-containing protein | WR30_RS17065 | Bamb_5743 | -3,3 | -2,0 | -3,2 |
| hypothetical protein | WR30_RS19090 | Bamb_6303 | 1,0 | 6,0 | 3,6 |
| amino acid adenylation protein | WR30_RS20005 | Bamb_6472 | 9,0 | 68,1 | 17,6 |
| extracellular ligand-binding receptor | WR30_RS18460 | BamMC406_1615 | 4,9 | 5,0 | 4,1 |
| NADP oxidoreductase coenzyme F420-dependent | WR30_RS15685 | BamMC406_1814 | -14,3 | -18,3 | -13,1 |
| LemA family protein | WR30_RS31555 | BamMC406_3391 | 57,3 | 1,8 | 57,7 |
| protein of unknown function DUF477 | WR30_RS31560 | BamMC406_3392 | 64,0 | 3,1 | 54,9 |
| AMP-dependent synthetase and ligase HhqA | WR30_RS17255 | BamMC406_5523 | 1,5 | 11,3 | 4,9 |
| hypothetical protein HhqB | WR30_RS17250 | BamMC406_5524 | 1,5 | 5,1 | 4,5 |
| Beta-ketoacyl-acyl-carrier-protein synthase I HhqC | WR30_RS17245 | BamMC406_5525 | 1,4 | 13,4 | 2,4 |
| 3-oxoacyl-ACP synthase HhqD | WR30_RS17240 | BamMC406_5526 | 1,8 | 4,5 | 1,6 |
| beta-lactamase domain-containing protein HhqE | WR30_RS17235 | BamMC406_5527 | -1,7 | 7,6 | 2,9 |
| AMP-dependent synthetase and ligase | WR30_RS17230 | BamMC406_5528 | 1,1 | 7,5 | 3,2 |
| aldehyde oxidase and xanthine dehydrogenase molybdopterin binding | WR30_RS19100 | BamMC406_6005 | 5,0 | 13,0 | 2,3 |
| integral membrane sensor signal transduction histidine kinase | WR30_RS29435 | BamMC406_6227 | 11,4 | 1,3 | 16,7 |
| hypothetical protein | WR30_RS29440 | BamMC406_6228 | 249,0 | 13,3 | 93,1 |
| alkylhydroperoxidase like protein, AhpD family | WR30_RS29445 | BamMC406_6230 | 54,6 | 4,2 | 49,9 |
| putative cation efflux protein | WR30_RS29450 | BamMC406_6231 | 8,1 | 3,2 | 38,9 |
| heavy metal translocating P-type ATPase | WR30_RS16560 | BamMC406_6233 | 16,7 | 3,2 | 22,9 |
| UspA domain protein | WR30_RS16565 | BamMC406_6234 | 16,9 | 6,4 | 29,7 |
| pterin-4-alpha-carbinolamine dehydratase PhhB | WR30_RS36425 | BCAL0009 | 3,5 | -1,4 | 4,0 |
| putative branched-chain amino acid ABC transporter periplasmic protein | WR30_RS36465 | BCAL0017 | 1,7 | 3,8 | 1,4 |
| putative branched-chain amino acid ABC transporter periplasmic substrate binding protein | WR30_RS36480 | BCAL0020 | -1,1 | 1,0 | 3,1 |
| putative branched-chain amino acid ABC transporter permease | WR30_RS36485 | BCAL0021 | 1,4 | 1,1 | 3,9 |
| putative branched-chain amino acid ABC transporter ATP-binding membrane protein | WR30_RS36490 | BCAL0022 | 2,5 | 1,4 | 6,9 |
| putative branched-chain amino acid ABC transporter ATP-binding protein | WR30_RS36495 | BCAL0023 | 1,3 | 2,2 | 7,5 |
| trifunctional transcriptional regulator/proline dehydrogenase/pyrroline-5-carboxylate dehydrogenase PutA | WR30_RS21855 | BCAL0042 | -5,0 | -1,4 | -1,1 |
| putative extracellular ligand-binding protein | WR30_RS21850 | BCAL0043 | -5,4 | -2,6 | -1,8 |
| AraC family regulatory protein | WR30_RS21785 | BCAL0056 | 3,4 | 4,4 | 4,8 |
| hypothetical protein (Conserved TM helix) | WR30_RS21780 | BCAL0057 | 6,6 | 16,1 | 10,9 |
| acetaldehyde dehydrogenase AcoD | WR30_RS21715 | BCAL0064 | 2,5 | 2,4 | 7,4 |
| hypothetical protein | WR30_RS21690 | BCAL0069 | 1,1 | -1,7 | -3,4 |
| glycine dehydrogenase [decarboxylating] GcvP | WR30_RS21670 | BCAL0073 | -5,3 | -3,2 | -1,3 |
| glycine cleavage system H protein GcvH | WR30_RS21665 | BCAL0074 | -4,7 | -2,5 | -0,9 |
| glycine cleavage system aminomethyltransferase GcvT | WR30_RS21660 | BCAL0075 | -4,8 | -3,9 | -1,6 |
| putative cytochrome | WR30_RS21635 | BCAL0080 | 7,1 | 6,8 | 1,2 |
| aquaporin ZAqpZ | WR30_RS21535 | BCAL0121 | 5,3 | 12,6 | 4,8 |
| histone-like nucleoid-structuring (H-NS) protein | WR30_RS21530 | BCAL0122 | 2,3 | 4,4 | 1,7 |
| adenosylhomocysteinase AhcY | WR30_RS21405 | BCAL0145 | -6,6 | -12,2 | -2,3 |
| putative membrane protein | WR30_RS21400 | BCAL0146 | -6,2 | -9,4 | -2,4 |
| 5,10-methylenetetrahydrofolate reductase MetF | WR30_RS21395 | BCAL0147 | -4,4 | -6,3 | -3,7 |
| extracellular ligand binding protein | WR30_RS21375 | BCAL0151 | 1,4 | 4,7 | 2,9 |
| putative cation efflux protein | WR30_RS21355 | BCAL0155 | 2,9 | 2,3 | 4,7 |
| putative outer membrane protein | WR30_RS21210 | BCAL0198 | 2,4 | 8,9 | 3,8 |
| putative lipoprotein | WR30_RS21205 | BCAL0199 | 1,9 | 3,7 | 1,5 |
| putative lipoprotein | WR30_RS21200 | BCAL0200 | 1,5 | 5,1 | 1,6 |
| NADP-dependent malic enzyme | WR30_RS21175 | BCAL0205 | -8,6 | -2,3 | -3,4 |
| putative outer membrane protein | WR30_RS21165 | BCAL0206A | -1,1 | -8,3 | -2,2 |
| 4-hydroxyphenylpyruvic acid dioxygenase HppD | WR30_RS21160 | BCAL0207 | -3,4 | -17,1 | -0,2 |
| acetyltransferase (GNAT) family protein | WR30_RS21150 | BCAL0209 | 5,8 | 1,6 | 1,3 |
| putative phenylacetic acid degradation NADH oxidoreductase | WR30_RS21135 | BCAL0212 | 1.6 | -7.2 | 22.6 |
| phenylacetic acid degradation protein | WR30_RS21130 | BCAL0213 | 1.1 | -7.5 | 12.4 |
| phenylacetic acid degradation protein | WR30_RS21125 | BCAL0214 | -1.1 | -13.7 | 39.9 |
| phenylacetate-CoA oxygenase | WR30_RS21120 | BCAL0215 | -1.6 | -13.5 | 29.0 |
| phenylacetate-CoA oxygenase | WR30_RS21115 | BCAL0216 | -1.1 | -14.3 | 41.0 |
| transcription antitermination protein NusG | WR30_RS04945 | BCAL0221 | -3,4 | -2,1 | 1,6 |
| 50S ribosomal protein L11 | WR30_RS04950 | BCAL0222 | -4,1 | -2,3 | 1,5 |
| 50S ribosomal protein L10 | WR30_RS04960 | BCAL0224 | -3,9 | -2,9 | 1,1 |
| 30S ribosomal protein S12 | WR30_RS04985 | BCAL0229 | -4,5 | -1,7 | 1,5 |
| 30S ribosomal protein S7 | WR30_RS04990 | BCAL0230 | -4,5 | -1,5 | 1,5 |
| elongation factor G | WR30_RS04995 | BCAL0231 | -4,7 | -2,3 | -1,1 |
| 30S ribosomal protein S10 | WR30_RS05005 | BCAL0233 | -4,1 | -2,3 | 1,2 |
| 50S ribosomal protein L3 | WR30_RS05010 | BCAL0234 | -4,0 | -2,5 | 1,7 |
| 50S ribosomal protein L4 | WR30_RS05015 | BCAL0235a | -4,5 | -2,8 | 1,8 |
| 50S ribosomal protein L23 | WR30_RS05020 | BCAL0236 | -5,7 | -2,2 | 1,6 |
| 50S ribosomal protein L2 | WR30_RS05025 | BCAL0237 | -5,7 | -2,8 | 1,3 |
| 30S ribosomal protein S19 | WR30_RS05030 | BCAL0238 | -3,4 | -1,7 | 1,4 |
| 50S ribosomal protein L22 | WR30_RS05035 | BCAL0239a | -4,8 | -2,7 | 1,2 |
| 30S ribosomal protein S3 | WR30_RS05040 | BCAL0240 | -5,2 | -2,6 | 1,1 |
| 50S ribosomal protein L16 | WR30_RS05045 | BCAL0241 | -3,9 | -2,4 | 1,1 |
| 50S ribosomal protein L29 | WR30_RS05050 | BCAL0242 | -4,1 | -2,5 | 1,0 |
| 30S ribosomal protein S17 | WR30_RS05055 | BCAL0243 | -3,2 | -1,8 | 1,2 |
| 50S ribosomal protein L14 | WR30_RS05060 | BCAL0244 | -4,2 | -2,5 | 1,2 |
| 50S ribosomal protein L24 | WR30_RS05065 | BCAL0245 | -4,4 | -1,7 | 1,3 |
| 50S ribosomal protein L5 | WR30_RS05070 | BCAL0246 | -5,1 | -2,1 | 1,1 |
| 30S ribosomal protein S14 | WR30_RS05075 | BCAL0247 | -4,7 | -1,8 | 1,4 |
| 30S ribosomal protein S8 | WR30_RS05080 | BCAL0248 | -3,4 | -2,1 | 1,0 |
| 50S ribosomal protein L6 | WR30_RS05085 | BCAL0249 | -4,3 | -1,9 | -1,1 |
| 50S ribosomal protein L18 | WR30_RS05090 | BCAL0250 | -4,3 | -2,3 | -1,5 |
| 30S ribosomal protein S5 | WR30_RS05095 | BCAL0251 | -3,9 | -2,2 | -1,3 |
| 50S ribosomal protein L15 | WR30_RS05105 | BCAL0253 | -4,6 | -1,9 | -1,2 |
| 30S ribosomal protein S13 | WR30_RS05125 | BCAL0257 | -3,2 | -2,3 | 1,2 |
| 30S ribosomal protein S11 | WR30_RS05130 | BCAL0258 | -3,7 | -3,1 | -1,4 |
| 30S ribosomal protein S4 | WR30_RS05135 | BCAL0259 | -3,1 | -2,5 | -1,1 |
| DNA-directed RNA polymerase subunit alpha RpoA | WR30_RS05140 | BCAL0260 | -3,1 | -2,0 | 1,2 |
| 50S ribosomal protein L17 | WR30_RS05145 | BCAL0261 | -4,4 | -2,4 | -1,9 |
| delta-aminolevulinic acid dehydratase HemB | WR30_RS05160 | BCAL0264 | 3,1 | -1,5 | 2,0 |
| 3-dehydroquinate synthase AroB | WR30_RS05235 | BCAL0280 | -2,6 | -1,4 | -3,0 |
| deoxyguanosinetriphosphate triphosphohydrolase-like protein | WR30_RS05240 | BCAL0281 | -1,6 | -1,6 | -3,1 |
| glycerol-3-phosphate transporter membrane protein | WR30_RS05255 | BCAL0284 | 1,3 | 3,5 | 2,1 |
| hypothetical protein | WR30_RS05275 | BCAL0288 | 2,4 | 3,0 | 3,7 |
| sodium:amino acid symporter family protein | WR30_RS05290 | BCAL0291 | -4,4 | -2,8 | -1,7 |
| thiamine-phosphate pyrophosphorylase ThiE | WR30_RS05335 | BCAL0300 | -1,5 | -3,3 | -1,8 |
| hypothetical protein | WR30_RS05345 | BCAL0302 | -3,6 | -2,0 | -2,3 |
| BolA-like protein | WR30_RS05380 | BCAL0309 | -4,0 | -1,9 | 1,3 |
| UDP-N-acetylglucosamine 1-carboxyvinyltransferase MurA | WR30_RS05385 | BCAL0310 | -6,1 | -1,8 | -2,2 |
| ATP phosphoribosyltransferase catalytic subunit HisG | WR30_RS05390 | BCAL0311 | -3,2 | -2,2 | -2,6 |
| putative amino acid permease | WR30_RS05725 | BCAL0369 | -1,5 | -3,8 | -2,3 |
| putative amino acid permease | WR30_RS05810 | BCAL0385 | 1,2 | 1,4 | -11,5 |
| indole-3-glycerol-phosphate synthase TrpC | WR30_RS05855 | BCAL0396 | -4,5 | -2,8 | 5,1 |
| anthranilate phosphoribosyltransferase TrpD | WR30_RS05860 | BCAL0397 | -3,2 | -1,7 | 4,2 |
| anthranilate synthase component II TrpG | WR30_RS05865 | BCAL0398 | -2,8 | -3,5 | 5,7 |
| anthranilate synthase component I TrpE | WR30_RS05870 | BCAL0399 | -2,7 | -2,6 | 6,7 |
| phenylacetate-coenzyme A ligase | WR30_RS05900 | BCAL0404 | -1.1 | -5.9 | 6.9 |
| phenylacetic acid degradation protein | WR30_RS05905 | BCAL0405 | -2.1 | -10.8 | 10.7 |
| probable enoyl-CoA hydratase | WR30_RS05910 | BCAL0406 | -1.7 | -9.8 | 8.9 |
| beta-ketoadipyl CoA thiolase | WR30_RS05915 | BCAL0407 | -1.8 | -9.4 | 14.5 |
| putative phenylacetic acid degradation oxidoreductase | WR30_RS05920 | BCAL0408 | -3.5 | -8.4 | 16.4 |
| enoyl-CoA hydratase | WR30_RS05925 | BCAL0409 | -1.6 | -2.0 | 3.6 |
| 50S ribosomal protein L34 | WR30_RS00125 | BCAL0423a | -3,9 | -2,9 | -1,4 |
| ribonuclease P protein component RnpA | WR30_RS00130 | BCAL0424 | -2,5 | -2,7 | -1,1 |
| spermidine N(1)-acetyltransferase SpeG | WR30_RS00180 | BCAL0433 | 1,7 | 1,8 | 3,2 |
| hypothetical protein | WR30_RS00185 | BCAL0434 | 2,2 | 57,3 | 1,4 |
| putative L-lactate permease | WR30_RS00225 | BCAL0441 | 4,7 | 21,0 | 3,0 |
| putative alcohol dehydrogenase | WR30_RS00470 | BCAL0488 | -1,2 | -5,7 | -2,4 |
| ATP-dependent protease ATP-binding subunit HslU | WR30_RS00565 | BCAL0500 | -3,7 | -2,5 | 2,1 |
| putative deoxygenases | WR30_RS00630 | BCAL0511 | 3,5 | 1,3 | 2,1 |
| hypothetical protein | WR30_RS00645 | BCAL0514 | 4,4 | 2,0 | 7,8 |
| putative coniferyl aldehyde dehydrogenase CalB | WR30_RS00670 | BCAL0518 | 2,4 | 3,3 | 1,1 |
| two-component regulatory system, response regulator protein | WR30_RS00760 | BCAL0534 | 7,8 | 1,2 | 2,1 |
| sensor kinase protein | WR30_RS00765 | BCAL0535 | 12,2 | 1,6 | 2,1 |
| putative ATPase | WR30_RS00795 | BCAL0540 | 1,9 | 4,6 | 1,5 |
| putative iron-sulfur protein | WR30_RS00805 | BCAL0542 | 3,6 | 2,5 | -2,8 |
| major facilitator superfamily protein | WR30_RS00810 | BCAL0543 | 2,3 | 3,2 | 1,7 |
| dipeptide transporter ATP-binding subunit DppE | WR30_RS00835 | BCAL0548 | 3,6 | -1,5 | 1,0 |
| subfamily M24B peptidase | WR30_RS00895 | BCAL0560 | 5,2 | 1,0 | 4,4 |
| conserved hypothetical protein | WR30_RS01010 | BCAL0585 | 1,5 | 5,0 | 3,8 |
| putrescine transport system permease PotI | WR30_RS01050 | BCAL0595 | 3,4 | 6,4 | 1,8 |
| putrescine ABC transporter permease PotH | WR30_RS01055 | BCAL0596 | 2,6 | 11,4 | -1,4 |
| putrescine ABC transporter ATP-binding protein PotG | WR30_RS01060 | BCAL0597 | 2,5 | 4,4 | -1,5 |
| putrescine ABC transporter binding exported protein PotF | WR30_RS01065 | BCAL0598 | 4,7 | 11,8 | 1,4 |
| putative aminotransferase | WR30_RS01070 | BCAL0599 | 9,4 | 26,2 | 1,0 |
| putative glutamine synthetase | WR30_RS01075 | BCAL0600 | 10,3 | 25,8 | 3,0 |
| putative gamma-glutamyl-gamma-aminobutyrate hydrolase | WR30_RS01080 | BCAL0601 | 24,7 | 22,9 | 1,6 |
| MerR family regulatory protein | WR30_RS01090 | BCAL0602 | 6,4 | 5,2 | -1,5 |
| gamma-glutamyl-gamma-aminobutyraldehyde dehydrogenase PuuC | WR30_RS01095 | BCAL0603 | 3,7 | 4,3 | -1,1 |
| putative cheavy metal binding protein | WR30_RS01100 | BCAL0604 | 4,8 | 5,1 | -1,4 |
| hypothetical protein | WR30_RS01105 | BCAL0605 | -5,1 | -1,7 | -5,1 |
| putative transport related, membrane protein | WR30_RS01110 | BCAL0606 | -4,7 | -1,5 | -6,2 |
| hypothetical protein | WR30_RS01150 | BCAL0616 | -1,4 | -3,6 | -2,0 |
| PfkB family carbohydrate kinase | WR30_RS01165 | BCAL0618 | -3,8 | -5,4 | -3,1 |
| putative N-acylglucosamine 2-epimerase | WR30_RS01170 | BCAL0619 | -5,3 | -6,2 | -2,3 |
| LacI family regulatory protein | WR30_RS01175 | BCAL0620 | -3,4 | -3,9 | -6,5 |
| LysR family regulatory protein | WR30_RS01200 | BCAL0625 | 2,0 | 5,0 | 5,3 |
| putative 2-nitropropane dioxygenase | WR30_RS01205 | BCAL0626 | 3,5 | 3,6 | 1,5 |
| putative hydrolase | WR30_RS01210 | BCAL0627 | 3,6 | 2,2 | -1,2 |
| sulfate-binding protein Sbp | WR30_RS01305 | BCAL0645 | -2,2 | -1,2 | -12,8 |
| putative pyruvate-flavodoxin oxidoreductase | WR30_RS01325 | BCAL0650 | -9,6 | -15,3 | 1,6 |
| biotin synthase BioB | WR30_RS01355 | BCAL0664 | -2,6 | -4,1 | -3,1 |
| 8-amino-7-oxononanoate synthase BioF | WR30_RS01365 | BCAL0666 | -2,9 | -4,2 | -2,3 |
| adenosylmethionine--8-amino-7-oxononanoate transaminase BioA | WR30_RS01370 | BCAL0667 | -4,8 | -5,8 | -4,9 |
| conserved hypothetical protein | WR30_RS01455 | BCAL0683 | 2,9 | -1,6 | 9,6 |
| putative cytidylyltransferase | WR30_RS01495 | BCAL0691 | -4,5 | -1,6 | -1,1 |
| putative trifunctional enoyl-CoA hydratase/3,2-trans-enoyl-CoA isomerase/3-hydroxyacyl-CoA dehydrogenase | WR30_RS01640 | BCAL0716 | -2,1 | -4,1 | -1,4 |
| putative acyl-CoA dehydrogenase | WR30_RS01645 | BCAL0717 | -1,5 | -5,4 | 1,3 |
| nitrogen regulatory protein P-II 1 GlnB1 | WR30_RS01705 | BCAL0729 | 2,5 | 2,8 | 11,4 |
| hypothetical protein | WR30_RS01830 | BCAL0755 | 1,2 | 3,4 | 2,9 |
| hypothetical protein | WR30_RS01870 | BCAL0763 | -7,9 | -3,1 | -1,1 |
| hypothetical protein | WR30_RS01900 | BCAL0769 | 2,3 | 4,1 | 5,8 |
| error-prone DNA polymerase DnaE2 | WR30_RS01905 | BCAL0770 | 2,1 | 2,7 | 3,0 |
| GntR family regulatory protein | WR30_RS01910 | BCAL0777 | -7,7 | -2,7 | -4,7 |
| hypothetical protein | WR30_RS01940 | BCAL0783 | 24,8 | 4,7 | 19,4 |
| cytochrome d ubiquinol oxidase subunit II CydB | WR30_RS01945 | BCAL0784 | 23,1 | 3,8 | 10,0 |
| cytochrome d ubiquinol oxidase subunit I CydA | WR30_RS01950 | BCAL0785 | 20,4 | 2,7 | 10,6 |
| hypothetical protein | WR30_RS01955 | BCAL0786 | 30,5 | 2,4 | 15,2 |
| 50S ribosomal protein L25/general stress protein Ctc | WR30_RS02020 | BCAL0799 | -4,9 | -3,3 | 1,0 |
| ribose-phosphate pyrophosphokinase PrsA | WR30_RS02025 | BCAL0800 | -2,9 | -4,1 | -1,3 |
| putative RNA polymerase sigma-54 factor RpoN | WR30_RS02085 | BCAL0813 | 4,0 | 1,6 | 4,4 |
| formyltetrahydrofolate deformylase PurU | WR30_RS02135 | BCAL0823 | -2,0 | -3,5 | -1,3 |
| putative ParA family protein | WR30_RS02170 | BCAL0830 | 31,8 | 4,2 | 21,3 |
| putative storage protein | WR30_RS02175 | BCAL0831 | 26,2 | 14,4 | 15,2 |
| putative Acetoacetyl-CoA reductase PhbB | WR30_RS02185 | BCAL0833 | 13,7 | 5,1 | 19,7 |
| hypothetical protein | WR30_RS02190 | BCAL0834 | 7,6 | 6,5 | 4,2 |
| putative exported protein | WR30_RS02195 | BCAL0835 | 2,1 | 1,9 | 8,4 |
| glycolate permease GlcA | WR30_RS02270 | BCAL0850 | 14,3 | -8,1 | 1,3 |
| putative iron-sulphur cluster containing protein | WR30_RS02275 | BCAL0851 | 14,2 | -39,1 | 3,9 |
| hypothetical protein | WR30_RS02280 | BCAL0852 | 37,5 | -6,4 | 1,4 |
| hypothetical protein | WR30_RS02285 | BCAL0853 | 15,6 | -28,6 | 2,5 |
| GntR family regulatory protein | WR30_RS02290 | BCAL0854 | -3,4 | -2,0 | -4,1 |
| putative exported protein | WR30_RS02305 | BCAL0858 | 7,2 | 1,9 | 2,7 |
| putative hydrolase | WR30_RS02310 | BCAL0859 | 8,5 | 2,2 | 2,5 |
| threonine dehydratase biosynthetic IlvA | WR30_RS02365 | BCAL0869 | -8,0 | -2,2 | -1,3 |
| dimethyladenosine transferase KsgA | WR30_RS02505 | BCAL0897 | -4,3 | -2,0 | -2,4 |
| glycyl-tRNA synthetase subunit beta GlyS | WR30_RS02535 | BCAL0903 | -3,6 | -1,8 | 1,1 |
| hypothetical protein | WR30_RS02545 | BCAL0905 | -1,7 | -1,4 | -4,8 |
| putative apolipoprotein N-acyltransferase | WR30_RS02550 | BCAL0906 | -4,0 | -4,9 | -7,5 |
| putative cation transporter efflux protein | WR30_RS02555 | BCAL0907 | -2,2 | -3,2 | -1,3 |
| putative oxidoreductase | WR30_RS02605 | BCAL0917 | -1,9 | -1,2 | -4,3 |
| conserved hypothetical protein | WR30_RS02660 | BCAL0928 | 1,7 | 4,6 | 3,1 |
| major facilitator superfamily protein | WR30_RS02795 | BCAL0950 | 3,5 | 5,4 | 1,0 |
| O-antigen polymerase family protein | WR30_RS02845 | BCAL0960 | -2,0 | -1,1 | -3,1 |
| AraC family regulatory protein | WR30_RS08620 | BCAL0969 | 1,1 | -1,6 | 3,2 |
| CreA protein 2 | WR30_RS08625 | BCAL0970 | -3,9 | -3,8 | -1,5 |
| hypothetical protein | WR30_RS08730 | BCAL0989 | -1,4 | 1,1 | 3,0 |
| putative glycerol-3-phosphate acyltransferase PlsX | WR30_RS08740 | BCAL0991 | -1,7 | -1,8 | -3,6 |
| putative DNA-binding protein | WR30_RS08895 | BCAL1023 | 6,3 | -1,3 | 5,2 |
| radical SAM superfamily protein | WR30_RS09015 | BCAL1051 | -4,5 | -1,9 | 2,3 |
| periplasmic solute-binding protein | WR30_RS09085 | BCAL1065 | -3,0 | -4,2 | -1,2 |
| hypothetical protein | WR30_RS09090 | BCAL1068 | 1,6 | 1,2 | 3,3 |
| putative cyclic-di-GMP signaling protein | WR30_RS09095 | BCAL1069 | 1,3 | 1,6 | 4,4 |
| multidrug resistance protein MdtC | WR30_RS09120 | BCAL1079 | -2,2 | -3,9 | -6,1 |
| multidrug resistance protein MdtB | WR30_RS09125 | BCAL1080 | -3,5 | -5,5 | -7,6 |
| multidrug resistance protein MdtA precursor | WR30_RS09130 | BCAL1081 | -2,4 | -6,4 | -9,5 |
| IclR family regulatory protein | WR30_RS09135 | BCAL1082 | -1.6 | -5.7 | -6.3 |
| putative amino acid transport system, membrane protein | WR30_RS09190 | BCAL1094 | -3,0 | -3,9 | -3,4 |
| thiamine biosynthesis protein ThiC | WR30_RS09240 | BCAL1104 | -2,3 | -4,5 | -3,8 |
| hypothetical protein | WR30_RS09330 | BCAL1211 | -1,8 | -2,3 | -3,7 |
| 2-oxoisovalerate dehydrogenase alpha subunit BkdA1 | WR30_RS09335 | BCAL1212 | 1,3 | -33,3 | -11,1 |
| 2-oxoisovalerate dehydrogenase beta subunit BkdA2 | WR30_RS09340 | BCAL1213 | 1,1 | -32,0 | -8,2 |
| lipoamide acyltransferase component of branched-chain alpha-keto acid dehydrogenase complex BkdB | WR30_RS09345 | BCAL1214 | 1,9 | -23,6 | -10,0 |
| dihydrolipoamide dehydrogenase LpdV | WR30_RS09350 | BCAL1215 | 2,4 | -4,8 | -16,8 |
| putative transport-related membrane protein | WR30_RS09370 | BCAL1220 | -4,3 | -1,8 | -1,4 |
| putative porin | WR30_RS09375 | BCAL1221 | -1,2 | -4,6 | -2,9 |
| putative malonate decarboxylase alpha-subunit | WR30_RS09495 | BCAL1240 | -2,6 | -2,5 | -3,5 |
| putative phosphoribosyl transferase protein | WR30_RS09535 | BCAL1248 | 6,5 | 2,9 | 1,4 |
| putative proline/betaine transporter | WR30_RS09555 | BCAL1252 | -1,9 | -6,9 | -1,1 |
| leucine export protein LeuE | WR30_RS09600 | BCAL1261 | 8,5 | 1,8 | 1,0 |
| MATE family transporter protein | WR30_RS09710 | BCAL1285 | -4,1 | -3,8 | -6,7 |
| conserved hypothetical protein | WR30_RS09715 | BCAL1286 | -1,2 | -2,6 | -4,2 |
| family M23 peptidase | WR30_RS09725 | BCAL1288 | -3,7 | -3,3 | -3,0 |
| conserved hypothetical protein | WR30_RS18400 | BCAL1294 | 4,6 | 7,3 | 9,7 |
| glucosamine--fructose-6-phosphate aminotransferase GlmS3 | WR30_RS09745 | BCAL1321 | -3,2 | -3,6 | -2,1 |
| putative hydroxyquinol 1,2-dioxygenase | WR30_RS09805 | BCAL1342 | 4,3 | -2,6 | 1,4 |
| putative porin | WR30_RS09865 | BCAL1368 | -1,9 | -7,6 | -4,1 |
| citrate transporter | WR30_RS09890 | BCAL1377 | -1,1 | -16,1 | -1,8 |
| hypothetical protein | WR30_RS09975 | BCAL1392 | 3,8 | 2,4 | -1,9 |
| putative glucose 1-dehydrogenase | WR30_RS10155 | BCAL1425 | 2,4 | 34,1 | 6,7 |
| hypothetical protein | WR30_RS10160 | BCAL1426 | 4,0 | 20,8 | 3,7 |
| putative ribose ABC transport system, substrate-binding exported protein | WR30_RS10185 | BCAL1431 | -2,6 | 1,7 | -6,8 |
| putative sugar ABC transporter ATP-binding protein | WR30_RS10190 | BCAL1432 | -2,0 | 1,5 | -3,1 |
| putative sugar transport system permease protein | WR30_RS10195 | BCAL1433 | -4,5 | -1,1 | -6,2 |
| putative myo-inositol catabolism protein | WR30_RS10200 | BCAL1434 | -10,1 | 1,4 | -4,2 |
| inositol 2-dehydrogenase Idh | WR30_RS10205 | BCAL1435 | -3,3 | 1,1 | -4,9 |
| putative oxidoreductase | WR30_RS10215 | BCAL1437 | -5,2 | 1,1 | -5,2 |
| putative fusaric acid resistance transport protein | WR30_RS10295 | BCAL1453 | 1,0 | -1,2 | 7,9 |
| LysR family regulatory protein | WR30_RS10315 | BCAL1457 | 2,5 | -3,4 | 2,0 |
| hypothetical protein | WR30_RS10510 | BCAL1498 | 3,5 | 1,3 | 2,9 |
| dihydrolipoamide succinyltransferase SucB | WR30_RS10600 | BCAL1516 | -3,7 | -2,3 | -1,1 |
| putative lipoprotein | WR30_RS10620 | BCAL1520 | 3,7 | -1,3 | -1,4 |
| putative sigma-54 related transcriptional regulatory protein | WR30_RS10700 | BCAL1536 | 1,8 | 2,7 | 3,5 |
| putative sugar ABC transporter lipoprotein | WR30_RS10760 | BCAL1548 | -4,7 | 1,7 | 1,1 |
| molybdenum-pterin binding protein II MopII | WR30_RS10905 | BCAL1622 | -13,5 | 1,4 | -1,2 |
| putative oxidoreductase | WR30_RS11385 | BCAL1639 | -4,2 | -1,7 | -1,7 |
| aliphatic sulfonate utilization regulatory protein SsuR | WR30_RS11470 | BCAL1656 | -8,1 | -1,6 | -1,5 |
| periplasmic solute-binding protein | WR30_RS11585 | BCAL1668 | -37,5 | -2,3 | -2,3 |
| TetR family regulatory protein (pseudogene) | WR30_RS11620 | BCAL1672 | -4,1 | -12,0 | -13,4 |
| periplasmic multidrug efflux lipoprotein AmrA | WR30_RS11625 | BCAL1674 | -2,8 | -6,7 | -12,2 |
| multidrug efflux system transporter protein AmrB | WR30_RS11630 | BCAL1675 | -9,7 | -14,3 | -18,5 |
| multidrug efflux system outer membrane protein | WR30_RS11635 | BCAL1676 | -2,5 | -13,2 | -18,9 |
| hypothetical protein | WR30_RS11765 | BCAL1704 | -2,8 | -3,1 | -2,6 |
| cobyrinic acid a,c-diamide synthase CobB | WR30_RS11770 | BCAL1705 | -4,6 | -3,8 | -3,0 |
| cob(I)yrinic acid a,c-diamide adenosyltransferase CobO | WR30_RS11775 | BCAL1706 | -1,7 | -2,8 | -3,9 |
| putative cobalamin biosynthesis-related protein CobW | WR30_RS11795 | BCAL1710 | -5,5 | -6,1 | -7,5 |
| major facilitator superfamily protein | WR30_RS12055 | BCAL1754 | -2,5 | -3,5 | -2,5 |
| OsmC-like protein | WR30_RS12135 | BCAL1766 | 3,9 | -1,4 | 9,3 |
| putative DNA-binding protein | WR30_RS12290 | BCAL1785 | -5,8 | -5,2 | -4,7 |
| putative saccharopine dehydrogenase | WR30_RS12355 | BCAL1796 | -6,6 | -5,1 | 5,7 |
| hypothetical protein | WR30_RS12390 | BCAL1799 | -3,2 | -1,5 | -2,5 |
| conserved hypothetical protein | WR30_RS12395 | BCAL1800 | -2,1 | -2,0 | -3,9 |
| 2-ketogluconate reductase TkrA | WR30_RS12415 | BCAL1803 | 20,8 | 1,6 | 12,6 |
| major facilitator superfamily protein | WR30_RS12420 | BCAL1804 | 7,8 | 1,7 | 4,7 |
| putative transport-related, membrane protein | WR30_RS12445 | BCAL1809 | -1,9 | -1,7 | -3,7 |
| metallo-beta-lactamase superfamily protein | WR30_RS12485 | BCAL1818 | 17,0 | 1,1 | 4,6 |
| hypothetical protein | WR30_RS12490 | BCAL1819 | 17,4 | 1,4 | 2,5 |
| hypothetical protein | WR30_RS12520 | BCAL1825 | 7,8 | 1,1 | 7,6 |
| putative outer membrane protein | WR30_RS12525 | BCAL1829 | 82,1 | 9,2 | 35,5 |
| putative 2-nitropropane dioxygenase | WR30_RS12530 | BCAL1830 | 48,8 | 3,1 | 8,9 |
| UDP glycosyltransferase | WR30_RS12575 | BCAL1838 | -1,1 | -1,3 | -6,5 |
| putative asparagine synthase | WR30_RS12580 | BCAL1839 | 1,0 | -1,2 | -6,0 |
| putative dehydrogenase | WR30_RS12635 | BCAL1850 | -4,3 | -1,7 | -4,7 |
| LysR family regulatory protein | WR30_RS12640 | BCAL1851 | -3,7 | -2,4 | -2,8 |
| putative transcription accessory protein Tex | WR30_RS12750 | BCAL1870 | -4,8 | -3,9 | -1,7 |
| protein HflC | WR30_RS12780 | BCAL1876 | -3,5 | -1,6 | -3,6 |
| 23S rRNA 5-methyluridine methyltransferase RumA | WR30_RS12845 | BCAL1889 | -2,3 | -3,2 | -1,7 |
| recombination protein RecR | WR30_RS12885 | BCAL1897 | -4,7 | -2,0 | -1,4 |
| 50S ribosomal protein L31 type B | WR30_RS12925 | BCAL1905 | -5,1 | -1,8 | 1,2 |
| acetoin:2,6-dichlorophenolindophenol oxidoreductase subunit beta AcoA | WR30_RS12950 | BCAL1910 | -2,1 | 44,9 | -2,1 |
| acetoin:2,6-dichlorophenolindophenol oxidoreductase subunit alpha AcoA | WR30_RS12955 | BCAL1911 | -1,1 | 69,6 | -3,3 |
| heat-shock protein ClpB | WR30_RS12995 | BCAL1919 | 3,0 | -1,5 | 9,3 |
| threonine synthase ThrC | WR30_RS13025 | BCAL1925 | -4,5 | -1,8 | -1,2 |
| homoserine dehydrogenase Hom | WR30_RS13030 | BCAL1926 | -7,6 | -1,9 | 1,1 |
| putative undecaprenyl-phosphate 4-deoxy-4-formamido-l-arabinose transferase | WR30_RS13060 | BCAL1932 | -3,3 | -2,7 | -2,0 |
| putative phosphorous metabolism-related protein | WR30_RS13085 | BCAL1937 | -3,6 | -2,9 | -2,6 |
| cysteine peptidase, family C40 | WR30_RS13090 | BCAL1938 | 1,3 | -1,5 | -3,6 |
| putative integral membrane transport protein | WR30_RS33525 | BCAL1939 | -2,0 | -1,6 | -3,8 |
| 30S ribosomal protein S18 | WR30_RS13115 | BCAL1943 | -3,6 | -2,4 | 1,0 |
| putative lipase | WR30_RS13245 | BCAL1969 | -3,8 | -1,1 | -1,2 |
| putative lipoprotein | WR30_RS13295 | BCAL1974 | 1,9 | 5,9 | 1,9 |
| BolA-like protein | WR30_RS13340 | BCAL1984 | 1,2 | -5,7 | -2,0 |
| putative exported isomerase | WR30_RS13345 | BCAL1985 | -3,7 | -3,2 | -2,4 |
| 2-dehydropantoate 2-reductase | WR30_RS13430 | BCAL2000 | -1,4 | -1,3 | -6,2 |
| hypothetical protein | WR30_RS13570 | BCAL2027 | 3,6 | 4,0 | 1,8 |
| LysR family regulatory protein | WR30_RS13575 | BCAL2028 | -2,0 | -3,0 | -3,8 |
| putative deaminase | WR30_RS13650 | BCAL2045 | -1,7 | -1,3 | -7,4 |
| putative exonuclease | WR30_RS13715 | BCAL2060 | -1,6 | -4,2 | -1,8 |
| hypothetical protein | WR30_RS13790 | BCAL2073 | -1,9 | 1,0 | -3,5 |
| isocitrate lyase AceA | WR30_RS14020 | BCAL2118 | 2,7 | -2,5 | 4,5 |
| putative patatin-like phospholipase | WR30_RS14100 | BCAL2127 | -2,6 | -5,9 | -4,4 |
| cytochrome O ubiquinol oxidase protein CyoD | WR30_RS14135 | BCAL2141 | 1,5 | -5,0 | -9,7 |
| cytochrome o ubiquinol oxidase subunit III CyoC | WR30_RS14140 | BCAL2142 | 1,0 | -10,8 | -9,3 |
| ubiquinol oxidase polypeptide I CyoB | WR30_RS14145 | BCAL2143 | -1,6 | -8,2 | -7,1 |
| ubiquinol oxidase polypeptide II precursor CyoA | WR30_RS14150 | BCAL2144 | -1,1 | -10,5 | -7,1 |
| acetyl-CoA carboxylase carboxyltransferase subunit alpha AccA | WR30_RS14180 | BCAL2148 | -3,2 | -1,9 | -1,2 |
| hypothetical protein | WR30_RS14195 | BCAL2151 | -1,7 | -1,7 | -3,6 |
| 2-dehydro-3-deoxyphosphooctonate aldolase | WR30_RS14350 | BCAL2180 | -3,4 | -2,1 | 1,1 |
| putative single-stranded-DNA-specific exonuclease | WR30_RS14390 | BCAL2188 | -4,3 | -3,8 | -1,7 |
| lysyl-tRNA synthetase LysS | WR30_RS14405 | BCAL2190 | -5,8 | -2,7 | 1,2 |
| chaperone protein HscA | WR30_RS14425 | BCAL2194 | -4,1 | -1,6 | -2,2 |
| co-chaperone HscB | WR30_RS14430 | BCAL2195 | -4,3 | 1,0 | -1,6 |
| scaffold protein | WR30_RS14440 | BCAL2197 | -3,4 | -2,3 | -2,3 |
| cysteine desulfurase IscS | WR30_RS14445 | BCAL2198 | -4,1 | -2,4 | -2,6 |
| D-alanyl-D-alanine endopeptidase | WR30_RS14480 | BCAL2205 | 2,0 | 4,5 | 2,9 |
| phasin-like protein PhaP | WR30_RS14485 | BCAL2206 | 2,2 | -1,3 | 5,3 |
| putative ATP-dependent helicase | WR30_RS14595 | BCAL2230 | -3,5 | -3,2 | -2,4 |
| putative glutamine synthetase | WR30_RS14665 | BCAL2238 | -1,3 | -4,2 | 1,6 |
| HutG protein | WR30_RS14675 | BCAL2240 | -4,8 | -1,5 | -1,1 |
| putative imidazolonepropionase | WR30_RS14685 | BCAL2242 | -11,7 | 1,2 | -1,2 |
| conserved hypothetical protein | WR30_RS14690 | BCAL2243 | -21,4 | -1,3 | -1,4 |
| urocanate hydratase HutU | WR30_RS14695 | BCAL2244 | -22,0 | -2,1 | 1,4 |
| putative histidine utilization repressor | WR30_RS14700 | BCAL2245 | -30,1 | 1,1 | -1,4 |
| histidine ammonia-lyase HutH | WR30_RS14705 | BCAL2246 | -21,9 | -1,2 | 1,3 |
| conserved hypothetical protein | WR30_RS14775 | BCAL2267 | -1,2 | -1,1 | -3,2 |
| hypothetical protein | WR30_RS14785 | BCAL2269 | -1,6 | -1,9 | -3,1 |
| putative toxic anion resistance protein | WR30_RS14795 | BCAL2271 | -2,2 | -1,5 | -3,7 |
| hypothetical protein | WR30_RS14805 | BCAL2273 | 4,7 | 2,6 | 1,3 |
| putative exported protein | WR30_RS14800 | BCAL2274 | 3,6 | 2,6 | 1,3 |
| hypothetical protein | WR30_RS14815 | BCAL2277 | 6,9 | 5,7 | 1,7 |
| hypothetical protein | WR30_RS04875 | BCAL2315 | 4,7 | 1,6 | 2,5 |
| family M14 peptidase | WR30_RS04865 | BCAL2317 | -3,8 | -3,3 | -2,1 |
| NADH dehydrogenase subunit I | WR30_RS04770 | BCAL2336 | 1,0 | -3,5 | -2,0 |
| NADH dehydrogenase subunit H | WR30_RS04765 | BCAL2337 | -1,3 | -2,7 | -1,3 |
| NADH dehydrogenase subunit G | WR30_RS04760 | BCAL2338 | -2,0 | -3,3 | -1,3 |
| NADH dehydrogenase I chain F | WR30_RS04755 | BCAL2339 | -1,3 | -3,1 | -1,4 |
| NADH dehydrogenase subunit E | WR30_RS04750 | BCAL2340 | -1,4 | -3,1 | -1,5 |
| NADH dehydrogenase subunit D | WR30_RS04745 | BCAL2341 | -1,3 | -2,7 | -1,5 |
| NADH dehydrogenase subunit C | WR30_RS04740 | BCAL2342 | -1,9 | -3,8 | -1,5 |
| NADH dehydrogenase subunit B | WR30_RS04735 | BCAL2343 | -2,4 | -3,2 | -1,3 |
| NADH dehydrogenase subunit A | WR30_RS04730 | BCAL2344 | -1,8 | -2,4 | -1,1 |
| putative exported protein | WR30_RS04440 | BCAL2401 | 3,8 | 3,5 | 3,1 |
| chaperone protein HtpG | WR30_RS04245 | BCAL2442 | -4,5 | -3,5 | 1,2 |
| putative metal transport integral membrane protein | WR30_RS04205 | BCAL2450 | 3,2 | 4,3 | 2,0 |
| DNA topoisomerase IV subunit B ParE | WR30_RS04180 | BCAL2455 | -3,5 | -2,2 | -1,6 |
| putative exported protein | WR30_RS04115 | BCAL2463 | -2,2 | -4,1 | -13,5 |
| hypothetical protein | WR30_RS04020 | BCAL2607 | 19,2 | 2,0 | 2,9 |
| putative exported outer membrane porin protein | WR30_RS03980 | BCAL2615 | -1,3 | 22,3 | 5,5 |
| putative porphyrin biosynthesis related protein | WR30_RS03930 | BCAL2628 | 3,8 | 3,6 | 2,2 |
| putative exported protein | WR30_RS09405 | BCAL2634a | 1,3 | 4,8 | 1,7 |
| putative exported protein | WR30_RS09420 | BCAL2635 | 3,9 | 4,9 | 1,4 |
| putative fimbriae chaperone | WR30_RS09415 | BCAL2636 | 4,0 | 4,3 | 1,2 |
| putative fimbriae usher protein | WR30_RS09410 | BCAL2637 | 1,4 | 7,7 | 0,9 |
| putative ornithine decarboxylase | WR30_RS03885 | BCAL2641 | -5,2 | -3,1 | -1,2 |
| putative aspartate 1-decarboxylase PanD | WR30_RS03830 | BCAL2652 | -1,3 | -1,9 | -3,2 |
| putative vitamin B12 transport protein | WR30_RS03795 | BCAL2660 | -1,9 | -1,8 | -6,0 |
| phosphoglycerate mutase family | WR30_RS03790 | BCAL2661 | -3,4 | -2,9 | -4,5 |
| cobalamin synthase CobS | WR30_RS03785 | BCAL2662 | -2,6 | -2,1 | -5,4 |
| nicotinate-nucleotide--dimethylbenzimidazole phosphoribosyltransferase CobT | WR30_RS03780 | BCAL2663 | -2,0 | -3,4 | -8,6 |
| ABC transporter ATP-binding protein | WR30_RS03775 | BCAL2664 | 1,1 | 1,7 | -5,5 |
| putative transmembrane ABC transporter permease | WR30_RS03770 | BCAL2665 | 4,8 | 1,3 | -4,4 |
| conserved hypothetical protein | WR30_RS03725 | BCAL2674 | -2,7 | -1,9 | -3,6 |
| putative sulfate adenylyltransferase subunit 1 CysN | WR30_RS03690 | BCAL2681 | -6,5 | -1,2 | -4,0 |
| putative sulfate adenylyltransferase subunit 2 CysD1 | WR30_RS03685 | BCAL2682 | -3,3 | -2,1 | -4,0 |
| phosphoadenosine phosphosulfate reductase CysH | WR30_RS03680 | BCAL2683 | -3,9 | -1,6 | -4,1 |
| hypothetical protein | WR30_RS03675 | BCAL2684 | -8,3 | 1,0 | -1,8 |
| putative sulfite reductase CysI | WR30_RS03670 | BCAL2685 | -9,2 | -1,2 | -3,0 |
| aminotransferase class-III | WR30_RS03585 | BCAL2701 | -2,9 | -4,3 | -3,0 |
| hypothetical protein | WR30_RS03575 | BCAL2703 | -3,4 | -1,1 | -1,2 |
| putative transmembrane fatty acid desaturase | WR30_RS03505 | BCAL2719 | 2,2 | 1,1 | 3,9 |
| isoleucyl-tRNA synthetase IleS | WR30_RS03480 | BCAL2724 | -3,3 | -1,9 | 1,1 |
| cold shock-like protein | WR30_RS03440 | BCAL2732 | 4,8 | 17,1 | 3,9 |
| hypothetical protein | WR30_RS03430 | BCAL2734 | 3,4 | -2,2 | 6,1 |
| isocitrate dehydrogenase | WR30_RS03425 | BCAL2735 | -4,3 | -3,1 | -1,4 |
| high-affinity nickel transport protein HoxN | WR30_RS03395 | BCAL2740 | -3,7 | -4,9 | -5,9 |
| 30S ribosomal protein S20 | WR30_RS03285 | BCAL2765 | -3,3 | -1,4 | 1,2 |
| putative membrane protein | WR30_RS03260 | BCAL2770 | -2,0 | -1,9 | -4,4 |
| ThiF family protein | WR30_RS03205 | BCAL2781 | 8,3 | 1,3 | 7,8 |
| pyridoxamine 5'-phosphate oxidase PdxH | WR30_RS03200 | BCAL2782 | 3,4 | -1,2 | 5,0 |
| putative cyclopropane-fatty-acyl-phospholipid synthase | WR30_RS03195 | BCAL2783 | 3,8 | 1,0 | 4,7 |
| putative tryptophan 2,3-dioxygenase | WR30_RS03150 | BCAL2792 | -1,4 | -3,8 | 1,4 |
| subfamily S1B serine peptidase | WR30_RS02980 | BCAL2829 | -3,7 | -3,7 | -2,5 |
| Protein of unknown function (DUF4088) | WR30_RS08605 | BCAL2904 | 1,5 | 8,3 | 32,0 |
| hypothetical protein | WR30_RS08555 | BCAL2914 | -1,2 | -1,1 | -3,9 |
| 50S ribosomal protein L19 | WR30_RS08495 | BCAL2925 | -3,2 | -1,6 | 1,3 |
| radical SAM superfamily protein | WR30_RS08465 | BCAL2931 | 3,8 | 2,8 | 2,3 |
| leucine-responsive regulatory protein Lrp | WR30_RS08460 | BCAL2932 | 3,3 | 1,4 | 1,2 |
| ADP-l-glycero-D-manno-heptose-6-epimerase HldD | WR30_RS08390 | BCAL2944 | -4,1 | -2,2 | -1,5 |
| 30S ribosomal protein S1 | WR30_RS08360 | BCAL2950 | -3,4 | -2,2 | 1,2 |
| cytidylate kinase Cmk | WR30_RS08355 | BCAL2951 | -1,7 | -3,1 | -2,6 |
| possible regulatory protein | WR30_RS08295 | BCAL2975 | 13,6 | 4,8 | 23,8 |
| NAD-dependent formate dehydrogenase subunit gamma FdsG | WR30_RS08290 | BCAL2976 | -1,4 | 15,6 | -1,3 |
| hypothetical protein | WR30_RS08200 | BCAL2991 | -3,9 | -2,3 | -3,3 |
| transglycosylase associated protein | WR30_RS08160 | BCAL2998 | 2,4 | 4,1 | 1,4 |
| hypothetical protein | WR30_RS08140 | BCAL3003 | 3,9 | 2,5 | 4,3 |
| putative chorismate mutase | WR30_RS08135 | BCAL3004 | -3,7 | -2,0 | -2,7 |
| cold shock-like protein CspA | WR30_RS08125 | BCAL3006 | 11,2 | 7,8 | 2,6 |
| hypothetical protein | WR30_RS08120 | BCAL3007 | 7,7 | 8,1 | 2,2 |
| putative outer membrane porin protein | WR30_RS08115 | BCAL3008 | 6,5 | 5,6 | 5,1 |
| acetyltransferase (GNAT) family protein | WR30_RS07990 | BCAL3028 | 1,4 | 3,9 | -1,1 |
| maltose-binding protein MalE | WR30_RS07915 | BCAL3041 | -1,8 | 5,0 | 1,6 |
| coproporphyrinogen III oxidase HemN | WR30_RS07690 | BCAL3094 | 7,2 | -2,2 | 7,1 |
| hypothetical protein | WR30_RS07685 | BCAL3095 | 3,7 | -3,2 | 3,8 |
| 3-deoxy-D-manno-octulosonic-acid transferase WaaA | WR30_RS07610 | BCAL3110 | 3,9 | 1,0 | 1,6 |
| hypothetical protein WbxY | WR30_RS07605 | BCAL3111 | 3,4 | -1,6 | 3,4 |
| heptosyltransferase I WaaC | WR30_RS07600 | BCAL3112 | 5,9 | -1,8 | 3,5 |
| glycosyltransferase WbxA | WR30_RS07585 | BCAL3115 | -3,7 | -3,8 | -1,2 |
| UDP-glucose epimerase GalE | WR30_RS07575 | BCAL3117 | -2,3 | -3,6 | 1,3 |
| putative 1-acyl-SN-glycerol-3-phosphate acyltransferase PlsC | WR30_RS07450 | BCAL3137 | -5,2 | -2,2 | -3,4 |
| dihydroorotase PyrX | WR30_RS07445 | BCAL3138 | -3,6 | -1,4 | -1,4 |
| aspartate carbamoyltransferase catalytic subunit PyrB | WR30_RS07440 | BCAL3139 | -5,0 | -2,9 | -1,1 |
| chaperonin GroEL | WR30_RS07395 | BCAL3146 | -3,2 | -3,7 | 1,6 |
| co-chaperonin GroES | WR30_RS07390 | BCAL3147 | -3,3 | -5,8 | -1,4 |
| putative exported hydrolase | WR30_RS07310 | BCAL3165 | 2,3 | 4,1 | 2,9 |
| putative lipoprotein | WR30_RS07305 | BCAL3166 | 1,3 | 4,4 | 2,9 |
| putative xanthine dehydrogenase large subunit XdhB | WR30_RS07275 | BCAL3172 | 4,3 | 1,5 | 2,8 |
| xanthine dehydrogenase XdhA | WR30_RS07270 | BCAL3173 | 4,7 | 1,2 | 3,0 |
| LysR family regulatory protein | WR30_RS07245 | BCAL3178 | 6,6 | 1,9 | 2,8 |
| putative hydrolase | WR30_RS07220 | BCAL3183 | -3,6 | -14,2 | -1,1 |
| homogentisate 1,2-dioxygenase HmgA | WR30_RS07215 | BCAL3184 | -5,2 | -10,6 | 1,5 |
| 4-hydroxybenzoate transporter PcaK | WR30_RS07210 | BCAL3185 | -1,4 | -6,1 | -2,0 |
| hypothetical protein | WR30_RS07205 | BCAL3186 | 1,2 | -4,5 | 1,3 |
| putative oxidoreductase | WR30_RS07200 | BCAL3187 | -3,7 | -11,6 | -1,1 |
| putative TolR-related protein | WR30_RS07100 | BCAL3201 | -5,3 | -3,3 | -2,1 |
| possible TolA-related transport transmembrane protein | WR30_RS07095 | BCAL3202 | -4,9 | -2,1 | -2,5 |
| carboxymuconolactone decarboxylase family protein | WR30_RS07030 | BCAL3214 | 1,2 | 6,5 | 1,3 |
| adenylyl-sulfate kinase CysC | WR30_RS07020 | BCAL3216 | 3,1 | 3,5 | 4,0 |
| hypothetical protein | WR30_RS06970 | BCAL3263 | -2,8 | -3,2 | -1,9 |
| putative polynucleotide adenylyltransferase | WR30_RS06965 | BCAL3264 | -4,1 | -2,1 | -1,8 |
| putative deoxyguanosine kinase/deoxyadenosine kinase | WR30_RS06955 | BCAL3266 | -4,8 | -4,7 | -2,9 |
| putative 3-methyl-2-oxobutanoate hydroxymethyltransferase PanB | WR30_RS06950 | BCAL3267 | -8,1 | -3,5 | -5,8 |
| putative phospho-2-dehydro-3-deoxyheptonate aldolase AroG | WR30_RS06875 | BCAL3282 | -3,6 | -9,7 | -1,6 |
| putative ferritin DPS-family DNA binding protein | WR30_RS06825 | BCAL3297 | -4,7 | -22,2 | -8,5 |
| peroxidase/catalase KatB | WR30_RS06820 | BCAL3299 | -5,4 | -13,2 | -4,2 |
| hypothetical protein | WR30_RS06770 | BCAL3310 | -4,7 | -2,5 | -4,8 |
| putative glutathione S-transferase | WR30_RS06690 | BCAL3331 | 9,8 | -1,2 | 6,0 |
| tyrosyl-tRNA synthetase TyrZ | WR30_RS06625 | BCAL3344 | -3,1 | -1,7 | 1,0 |
| 30S ribosomal protein S9 | WR30_RS06610 | BCAL3347 | -4,3 | -2,7 | 1,4 |
| 50S ribosomal protein L13 | WR30_RS06605 | BCAL3348 | -3,0 | -2,4 | 1,4 |
| family C44 non-peptidase-like protein | WR30_RS06585 | BCAL3352 | 1,9 | 1,5 | 3,2 |
| putative glutamate dehydrogenase | WR30_RS06555 | BCAL3359 | 4,1 | 2,2 | 7,7 |
| phosphogluconate dehydratase Edd | WR30_RS06510 | BCAL3367 | -4,3 | 3,6 | 1,6 |
| allantoicase | WR30_RS06445 | BCAL3380 | -1,1 | -2,3 | -3,3 |
| putative L-arabinose transport system, exported protein | WR30_RS06325 | BCAL3405 | 1,4 | 3,4 | 1,3 |
| family S10 serine peptidase | WR30_RS06295 | BCAL3411 | 1,4 | 3,6 | -2,2 |
| 50S ribosomal protein L21 | WR30_RS06140 | BCAL3442 | -3,0 | -1,7 | 1,7 |
| type IV prepilin leader peptide type M1 GspO | WR30_RS06105 | BCAL3447 | -1,5 | -2,7 | -3,6 |
| NUDIX hydrolase | WR30_RS06085 | BCAL3450 | -6,7 | -2,8 | -2,8 |
| hypothetical protein | WR30_RS06080 | BCAL3451 | -4,2 | -1,9 | -1,1 |
| bifunctional ornithine acetyltransferase/N-acetylglutamate synthase protein ArgJ | WR30_RS06075 | BCAL3452 | -4,3 | -2,1 | -1,1 |
| putative outer membrane porin | WR30_RS05965 | BCAL3473 | 3,8 | 8,4 | 6,9 |
| putative type-b cytochrome | WR30_RS36105 | BCAL3476 | -1,4 | 8,1 | -7,6 |
| putative catalase | WR30_RS36110 | BCAL3477 | -1,7 | 7,8 | -4,9 |
| putative RNA polymerase sigma factor | WR30_RS36115 | BCAL3478 | -2,5 | 3,8 | -4,4 |
| RNA polymerase sigma factor SigJ | WR30_RS36150 | BCAL3486 | -3,3 | -3,2 | -2,7 |
| type III restriction system endonuclease | WR30_RS36185 | BCAL3493 | -3,8 | -4,1 | -1,9 |
| type III restriction-modification system methylase | WR30_RS36190 | BCAL3494 | -6,5 | -4,0 | -1,2 |
| LrgB family protein | WR30_RS36260 | BCAL3508 | 14,2 | -2,2 | 3,9 |
| LrgA family protein | WR30_RS36265 | BCAL3509 | 7,4 | 1,8 | 1,4 |
| hypothetical protein | WR30_RS36290 | BCAL3513 | 2,6 | 3,4 | 2,6 |
| putative sigma factor | WR30_RS29270 | BCAM0001 | -5,8 | -3,6 | -6,7 |
| ArsC family arsenate reductase | WR30_RS29275 | BCAM0002 | -3,0 | -2,4 | -1,5 |
| hypothetical protein | WR30_RS29305 | BCAM0008 | 2,0 | 3,0 | 3,2 |
| 2-amino-3-ketobutyrate coenzyme A ligase Kbl | WR30_RS29315 | BCAM0010 | 4,0 | -5,2 | 1,0 |
| tdh threonine 3-dehydrogenase | WR30_RS29320 | BCAM0011 | 5,8 | -3,7 | 1,6 |
| hypothetical protein | WR30_RS29390 | BCAM0025 | 4,2 | 1,5 | 3,5 |
| putative FHA-domain-containing protein | WR30_RS29495 | BCAM0028 | 3,7 | 1,9 | 9,3 |
| putative transporter-LysE family | WR30_RS29550 | BCAM0047 | -3,2 | -2,8 | -36,0 |
| chromosome replication initiation inhibitor | WR30_RS29555 | BCAM0048 | 3,6 | -4,1 | 1,8 |
| universal stress-related protein | WR30_RS29565 | BCAM0050 | 6,6 | 1,3 | 5,4 |
| 4-hydroxybenzoate 3-monooxygenase PobA | WR30_RS29600 | BCAM0057 | 1,0 | -3,3 | -5,9 |
| 3-oxoadipate CoA-transferase subunit A PcaI | WR30_RS29605 | BCAM0058 | -108,4 | -136,2 | -96,3 |
| 3-oxoadipate CoA-transferase subunit B PcaJ | WR30_RS29610 | BCAM0059 | -96,3 | -171,3 | -61,0 |
| 3-carboxy-cis,cis-muconate cycloisomerase PcaB | WR30_RS29615 | BCAM0060 | -14,3 | -50,0 | -25,0 |
| putative 3-oxoadipate enol-lactonase I | WR30_RS29620 | BCAM0061 | -27,9 | -38,9 | -26,4 |
| 4-carboxymuconolactone decarboxylase PcaC | WR30_RS29625 | BCAM0062 | -22,2 | -25,1 | -43,4 |
| putative 4-hydroxybenzoate transporter | WR30_RS29630 | BCAM0063 | -2,6 | -10,3 | -14,5 |
| hypothetical protein | WR30_RS29635 | BCAM0064 | 3,3 | 1,5 | 1,2 |
| putative transporter-LysE family | WR30_RS29640 | BCAM0065 | -1,2 | -8,1 | -1,9 |
| putative polysaccharide deacetylase | WR30_RS29745 | BCAM0082 | -17,9 | -3,9 | -1,5 |
| hypothetical protein | WR30_RS29750 | BCAM0083 | -22,5 | -5,9 | -2,9 |
| putative glycosyl transferase | WR30_RS29755 | BCAM0084 | -6,7 | -3,9 | -3,0 |
| putative sodium:dicarboxylate symporter family protein | WR30_RS29760 | BCAM0086 | -4,8 | -35,0 | -5,0 |
| hypothetical protein | WR30_RS29840 | BCAM0102 | 1,6 | 9,8 | 1,2 |
| hypothetical protein | WR30_RS16890 | BCAM0120 | -5,2 | -3,4 | -2,1 |
| 2-keto-3-deoxygluconate permease KdgT | WR30_RS30020 | BCAM0153 | 6,0 | 5,5 | 2,2 |
| putative type IV secretion system protein VirB6 | WR30_RS30750 | BCAM0328 | -1,2 | -3,7 | -2,8 |
| ABC transporter ATP-binding membrane protein | WR30_RS30840 | BCAM0355 | -1,8 | -1,9 | -3,1 |
| putative aldo/keto reductase | WR30_RS30845 | BCAM0356 | -1,2 | 6,8 | -2,0 |
| ArsR family regulatory protein | WR30_RS30865 | BCAM0358 | -3,6 | -2,5 | -10,2 |
| putative short chain dehydrogenase | WR30_RS30895 | BCAM0365 | -1,9 | -1,6 | -20,1 |
| hypothetical protein | WR30_RS30920 | BCAM0370 | 3,5 | 2,3 | 1,5 |
| putative lipoprotein | WR30_RS30990 | BCAM0382 | 2,0 | 4,1 | 1,3 |
| putative acetyltransferase | WR30_RS31060 | BCAM0392 | 4,2 | 7,2 | 3,1 |
| LysR family regulatory protein | WR30_RS31185 | BCAM0412a | -1,4 | -1,4 | -5,9 |
| hypothetical protein | WR30_RS31190 | BCAM0413 | 1,3 | 4,3 | 1,6 |
| hypothetical protein | WR30_RS31250 | BCAM0429 | -4,5 | -3,2 | -1,9 |
| hypothetical protein | WR30_RS31265 | BCAM0432 | -1,7 | -4,1 | -1,4 |
| conserved hypothetical protein | WR30_RS31315 | BCAM0438 | -1,7 | -9,8 | -14,2 |
| putative cation efflux protein | WR30_RS31320 | BCAM0439 | -1,9 | -8,5 | -17,0 |
| conserved hypothetical protein | WR30_RS31325 | BCAM0440 | 1,1 | -7,6 | -5,8 |
| putative ThiJ/PfpI family protein | WR30_RS31425 | BCAM0456 | -1,2 | -1,1 | -6,0 |
| Rieske [2Fe-2S] protein | WR30_RS31495 | BCAM0472 | -7,2 | -7,9 | -6,8 |
| LysR family regulatory protein | WR30_RS31715 | BCAM0501 | -6,5 | -1,6 | -1,4 |
| conserved hypothetical protein | WR30_RS31720 | BCAM0502 | -39,4 | -2,8 | 1,6 |
| CsbD-like protein | WR30_RS31750 | BCAM0507 | 2,7 | 3,5 | 1,9 |
| putative periplasmic binding protein | WR30_RS31755 | BCAM0508 | -12,0 | -1,1 | 1,1 |
| putative FAD dependent oxidoreductase | WR30_RS31760 | BCAM0509 | -5,0 | -1,3 | -1,1 |
| putative esterase | WR30_RS31765 | BCAM0511 | -4,3 | 1,1 | -4,3 |
| putative aminotransferase | WR30_RS31770 | BCAM0512 | -3,2 | 1,1 | 1,1 |
| putative aminotransferase protein | WR30_RS31870 | BCAM0525 | -24,8 | 1,5 | 1,2 |
| hypothetical protein | WR30_RS31985 | BCAM0535 | 1,0 | 4,4 | 1,9 |
| putative arylsulfatase | WR30_RS32135 | BCAM0569 | -13,8 | -1,7 | -3,8 |
| hypothetical protein | WR30_RS32170 | BCAM0576 | 31,6 | 7,7 | -2,3 |
| major facilitator superfamily protein | WR30_RS32175 | BCAM0577 | 20,7 | 2,4 | -3,3 |
| putative hydantoinase/oxoprolinase family protein | WR30_RS32180 | BCAM0578 | 7,6 | 1,7 | -1,6 |
| LysR family regulatory protein | WR30_RS18815 | BCAM0586 | 6,2 | 1,1 | 1,5 |
| AraC family regulatory protein | WR30_RS32390 | BCAM0631 | -2,3 | -3,5 | -4,5 |
| putative acetyl transferase-GNAT family | WR30_RS32395 | BCAM0632 | -3,0 | -2,0 | -5,3 |
| conserved hypothetical protein | WR30_RS32400 | BCAM0633 | -2,6 | -2,3 | -9,5 |
| hypothetical protein | WR30_RS32620 | BCAM0679 | 5,0 | 1,7 | 4,2 |
| putative membrane protein | WR30_RS32675 | BCAM0689 | -5,3 | -5,8 | -4,2 |
| putative NADPH-dependent FMN reductase | WR30_RS32690 | BCAM0692 | -2,3 | -6,4 | -2,3 |
| O-acetylhomoserine (thiol)-lyase | WR30_RS32830 | BCAM0721 | -7,7 | -2,3 | -3,3 |
| putative diguanylate cyclase | WR30_RS32975 | BCAM0748 | 5,0 | 1,6 | 3,0 |
| periplasmic lysine-arginine-ornithine-binding protein ArgT | WR30_RS33075 | BCAM0759 | -4,0 | -1,1 | 1,2 |
| histidine transport system permease HisM | WR30_RS33085 | BCAM0761 | -3,0 | 1,3 | 1,4 |
| histidine ABC transporter ATP-binding protein HisP | WR30_RS33090 | BCAM0762 | -6,9 | -1,7 | 2,2 |
| poly[D-(-)-3-hydroxybutyrate] depolymerase | WR30_RS33105 | BCAM0774 | 1,6 | 2,9 | 4,5 |
| glutathione-S-tranferase family protein | WR30_RS33115 | BCAM0775 | 14,9 | 1,4 | 17,3 |
| putative cNMP-binding domain-containing protein | WR30_RS33120 | BCAM0776 | 15,5 | 6,6 | 118,6 |
| hypothetical protein | WR30_RS33265 | BCAM0800 | -1,3 | 3,5 | 2,1 |
| LysR family regulatory protein | WR30_RS33270 | BCAM0801 | -4,2 | -1,7 | -1,6 |
| catechol 1,2-dioxygenase 1 CatA1 | WR30_RS33290 | BCAM0804 | 1,4 | -3,4 | -1,5 |
| muconate cycloisomerase I 1 CatB1 | WR30_RS33295 | BCAM0805 | 1,2 | -50,0 | -6,7 |
| putative aromatic oxygenase | WR30_RS33310 | BCAM0810 | 1,3 | -95,0 | -1,5 |
| putative aromatic oxygenase | WR30_RS33315 | BCAM0811 | 1,7 | -232,3 | -7,1 |
| putative aromatic hydrocarbons catabolism-related dioxygenase | WR30_RS33320 | BCAM0812 | 1,4 | -117,0 | -5,3 |
| putative aromatic hydrocarbons catabolism-related reductase | WR30_RS33325 | BCAM0813 | -3,7 | -21,4 | -7,8 |
| putative DNA binding protein | WR30_RS33335 | BCAM0815 | 1,0 | 1,1 | -4,6 |
| putative voltage gated chloride channel membrane protein | WR30_RS33400 | BCAM0827 | -1,9 | -1,7 | -2,7 |
| putative manganese transport protein, NRAMP family | WR30_RS33440 | BCAM0836 | -7,1 | -5,7 | -3,7 |
| hypothetical protein | WR30_RS33445 | BCAM0837 | -1,7 | -1,2 | -7,5 |
| short chain dehydrogenase | WR30_RS33450 | BCAM0839 | -4,1 | -6,9 | -3,1 |
| putative lipoprotein | WR30_RS33470 | BCAM0843 | 2,3 | 1,7 | 9,4 |
| putative endonuclease/exonuclease/phosphatase family protein | WR30_RS36085 | BCAM0845 | 4,9 | 3,2 | 1,4 |
| hypothetical protein | WR30_RS36045 | BCAM0853 | 1,1 | 3,9 | 2,9 |
| putative transcriptional regulator | WR30_RS35950 | BCAM0871 | 2,0 | 3,2 | 2,2 |
| conserved hypothetical protein | WR30_RS35865 | BCAM0888 | 7,1 | 1,0 | 2,7 |
| putative molybdenum transport system permease | WR30_RS35850 | BCAM0891 | 5,2 | 1,4 | 1,0 |
| AMP nucleosidase | WR30_RS35805 | BCAM0901 | -1,3 | -3,4 | -1,5 |
| conserved hypothetical protein | WR30_RS35695 | BCAM0921 | -5,4 | -4,1 | -3,9 |
| putative exported protein | WR30_RS35680 | BCAM0929 | -2,6 | -3,0 | -3,9 |
| putative cytochrome b561 | WR30_RS35590 | BCAM0946 | 3,3 | -1,3 | 1,3 |
| hypothetical protein | WR30_RS35585 | BCAM0946a | 2,1 | -1,5 | -3,8 |
| TonB-dependent receptor | WR30_RS35580 | BCAM0948 | 1,4 | -2,8 | -4,0 |
| ABC transporter ATP-binding protein | WR30_RS35560 | BCAM0952 | 8,3 | 7,2 | 1,3 |
| extracellular solute-binding protein | WR30_RS35555 | BCAM0953 | 3,1 | 3,9 | -1,1 |
| binding-protein-dependent transport system protein | WR30_RS35550 | BCAM0954 | 2,9 | 4,7 | -1,2 |
| binding-protein-dependent transport system protein | WR30_RS35545 | BCAM0955 | 2,1 | 3,3 | -1,4 |
| 2-methylcitrate dehydratase PrpD | WR30_RS35500 | BCAM0962 | -3,8 | -1,6 | -1,4 |
| hypothetical protein | WR30_RS35495 | BCAM0963 | -4,4 | -2,4 | -1,9 |
| malate dehydrogenase Mdh | WR30_RS35485 | BCAM0965 | -3,6 | -2,4 | -1,3 |
| putative succinate dehydrogenase cytochrome b556 subunit | WR30_RS35475 | BCAM0967 | -1,7 | -3,2 | -1,1 |
| succinate dehydrogenase flavoprotein subunit SdhA | WR30_RS35465 | BCAM0969 | -3,7 | -3,1 | -1,3 |
| succinate dehydrogenase iron-sulfur subunit SdhB | WR30_RS35460 | BCAM0970 | -2,9 | -3,2 | -1,1 |
| hypothetical protein | WR30_RS35455 | BCAM0971 | -2,8 | -3,6 | -1,4 |
| hypothetical protein (Protein of unknown function (DUF1479)) | WR30_RS35435 | BCAM0975 | 90,5 | 1,2 | 1,4 |
| putative amidinotransferase | WR30_RS35430 | BCAM0976 | 56,1 | 3,1 | 1,4 |
| AnsC family regulatory protein | WR30_RS35425 | BCAM0977 | 25,5 | -1,9 | -3,9 |
| lysine-specific permease LysP | WR30_RS35420 | BCAM0978 | 15,6 | 1,3 | -2,1 |
| putative glutathione-S-transferase | WR30_RS35415 | BCAM0979 | 3,4 | 1,5 | -1,5 |
| putative bifunctional folylpolyglutamate synthase/dihydrofolate synthase FolC | WR30_RS35330 | BCAM0995 | -4,0 | -1,3 | 1,0 |
| GCP-mannose 4,6-dehydratase Gca | WR30_RS35300 | BCAM1004 | 1,5 | 1,4 | 4,0 |
| putative porin | WR30_RS35240 | BCAM1015 | 4,3 | 3,9 | 3,3 |
| formate dehydrogenase iron-sulfur subunit FdnH | WR30_RS35220 | BCAM1019 | -4,8 | -1,6 | -2,9 |
| FdhE-like protein | WR30_RS35210 | BCAM1021 | -3,1 | -1,2 | 1,0 |
| putative membrane protein | WR30_RS35180 | BCAM1097 | -4,0 | -1,2 | -1,4 |
| NUDIX hydrolase | WR30_RS35175 | BCAM1098 | -3,6 | -1,9 | -3,5 |
| putrescine transporter PotE | WR30_RS35110 | BCAM1113 | 1,7 | 3,0 | -2,9 |
| LysR family regulatory protein | WR30_RS35105 | BCAM1114 | 1,1 | 2,4 | -5,9 |
| MarR family regulatory protein | WR30_RS34915 | BCAM1139 | 2,8 | -1,2 | 7,6 |
| 3-hydroxyisobutyrate dehydrogenase MmsB | WR30_RS34865 | BCAM1150 | 2,6 | -5,4 | -15,3 |
| methylmalonate-semialdehyde dehydrogenase | WR30_RS34860 | BCAM1151 | 3,3 | -15,3 | -12,4 |
| major facilitator superfamily protein | WR30_RS34855 | BCAM1152 | 11,0 | -22,0 | -16,8 |
| putative sulfatase | WR30_RS34835 | BCAM1158 | -2,4 | -1,4 | -4,5 |
| hypothetical protein | WR30_RS34805 | BCAM1164 | 20,5 | 3,4 | 3,5 |
| major facilitator superfamily protein | WR30_RS34770 | BCAM1171 | 1,3 | 19,8 | -2,1 |
| gamma-glutamylputrescine oxidoreductase PuuB | WR30_RS34765 | BCAM1172 | 1,1 | 28,4 | -1,5 |
| gamma-aminobutyrate permease GabP | WR30_RS34760 | BCAM1173 | 1,3 | 33,1 | -1,1 |
| putative molybdopterin oxidoreductase | WR30_RS34755 | BCAM1174 | -12,4 | 2,5 | -6,1 |
| putative dimethyl sulfoxide reductase subunit | WR30_RS34750 | BCAM1175 | -9,3 | 2,5 | -7,2 |
| putative dimethyl sulfoxide reductase subunit DmsC | WR30_RS34745 | BCAM1176 | -5,8 | 3,2 | -4,0 |
| IclR family regulatory protein | WR30_RS34740 | BCAM1177 | -4,7 | 4,4 | -6,5 |
| aerobic cobaltochelatase CobS subunit | WR30_RS34735 | BCAM1178 | -4,1 | 2,3 | -7,1 |
| aerobic cobaltochelatase subunit CobT | WR30_RS34730 | BCAM1179 | -5,2 | 2,9 | -6,8 |
| TonB-dependent siderophore receptor | WR30_RS21890 | BCAM1187 | -3,8 | -1,1 | 1,0 |
| two-component regulatory system sensor kinase | WR30_RS21940 | BCAM1195 | 3,7 | 5,1 | 6,2 |
| LysR family regulatory protein | WR30_RS21975 | BCAM1201 | -1,3 | -1,1 | -3,5 |
| hypothetical protein | WR30_RS21995 | BCAM1205 | -1,6 | 6,2 | -2,3 |
| ABC transporter ATP-binding membrane protein | WR30_RS22005 | BCAM1207 | 2,8 | 196,7 | 11,7 |
| glutamine ABC transporter periplasmic protein | WR30_RS22010 | BCAM1208 | 1,2 | 89,3 | 44,6 |
| glutamine ABC transporter permease GlnP | WR30_RS22015 | BCAM1209 | -1,2 | 64,0 | 16,7 |
| glutamine ABC transporter ATP-binding protein GlnQ | WR30_RS22020 | BCAM1210 | -1,2 | 37,5 | 14,3 |
| cysteine peptidase/transferase, family C45 | WR30_RS22025 | BCAM1211 | -1,4 | 24,6 | 14,6 |
| hypothetical protein | WR30_RS22030 | BCAM1212 | -2,5 | 26,5 | 5,8 |
| hypothetical protein | WR30_RS22035 | BCAM1213 | -9,8 | 36,5 | 6,1 |
| LysR family regulatory protein | WR30_RS22125 | BCAM1232 | 3,7 | 4,1 | 1,6 |
| putative acyltransferase | WR30_RS22170 | BCAM1241 | -1,3 | 3,1 | 1,5 |
| putative acetyl-CoA hydrolase/transferase | WR30_RS22225 | BCAM1250 | 5,0 | 1,2 | 4,1 |
| pseudo | WR30_RS22230 | BCAM1251 | 6,4 | 1,0 | 5,4 |
| MarR family regulatory protein | WR30_RS22235 | BCAM1254 | -1,5 | -1,9 | -3,0 |
| MerR family regulatory protein | WR30_RS22245 | BCAM1257 | -3,0 | -3,0 | -4,1 |
| putative DNA-binding protein | WR30_RS22255 | BCAM1258 | 9,2 | 2,1 | 5,4 |
| putative dihydrodipicolinate synthase | WR30_RS22295 | BCAM1266 | 27,7 | 1,3 | 1,3 |
| hypothetical protein | WR30_RS22355 | BCAM1280a | 2,0 | 5,5 | 1,6 |
| L-asparaginase | WR30_RS22405 | BCAM1291 | -1,4 | -4,4 | -1,3 |
| ABC transporter ATP-binding protein | WR30_RS22410 | BCAM1292 | -1,8 | -5,5 | 1,4 |
| ABC transporter, substrate-binding protein | WR30_RS22415 | BCAM1293 | -2,8 | -6,7 | -1,1 |
| ABC transporter, permease protein | WR30_RS22420 | BCAM1294 | -2,4 | -10,3 | -1,3 |
| ABC transporter permease | WR30_RS22425 | BCAM1295 | -1,5 | -11,1 | -1,8 |
| protocatechuate 3,4-dioxygenase subunit alpha PcaG | WR30_RS22450 | BCAM1299 | -3,6 | -3,5 | -5,3 |
| protocatechuate 3,4-dioxygenase beta chain PcaH | WR30_RS22455 | BCAM1300 | -12,6 | -24,4 | -5,9 |
| putative beta-glucosidas | WR30_RS22560 | BCAM1326 | 1,6 | 10,6 | 4,4 |
| acyl carrier protein | WR30_RS22655 | BCAM1347 | -1,6 | 3,8 | 4,6 |
| putative cyclic nucleotide-binding protein | WR30_RS22660 | BCAM1348 | 2,2 | 2,6 | 5,0 |
| putative regulatory protein (DksA; DnaK suppressor protein) | WR30_RS22680 | BCAM1351 | 42,8 | 2,3 | 10,7 |
| putative phosphoesterase | WR30_RS22685 | BCAM1352 | 12,4 | 2,8 | 2,3 |
| alanine dehydrogenase Ald | WR30_RS22690 | BCAM1353 | 5,9 | 3,7 | 1,0 |
| hypothetical protein | WR30_RS22695 | BCAM1354 | 6,9 | -1,5 | 1,9 |
| putative penicillin-binding protein | WR30_RS22725 | BCAM1362 | 3,3 | 1,5 | 2,0 |
| Protein of unknown function (DUF3564) | WR30_RS22730 | BCAM1363 | 26,9 | 16,2 | 24,3 |
| 5-carboxymethyl-2-hydroxymuconate isomerase HpaF | WR30_RS22765 | BCAM1370 | -2,2 | -3,5 | -1,3 |
| two-component regulatory system response regulator protein | WR30_RS22825 | BCAM1382 | -1,3 | -1,4 | -4,0 |
| hypothetical protein | WR30_RS22985 | BCAM1409 | -1,8 | -1,8 | -1,9 |
| putative cyclic nucleotide binding protein | WR30_RS23070 | BCAM1422 | 4,3 | 1,5 | 1,7 |
| hypothetical protein | WR30_RS23085 | BCAM1425 | -1,9 | 4,5 | -1,3 |
| voltage-gated potassium channel | WR30_RS23090 | BCAM1426 | -1,7 | -4,9 | -2,6 |
| LysE family transporter | WR30_RS23095 | BCAM1427 | 5,1 | -3,1 | -3,0 |
| Major Facilitator Superfamily protein | WR30_RS23170 | BCAM1439 | -5,4 | -4,0 | -11,2 |
| LysR family regulatory protein | WR30_RS23175 | BCAM1440 | -12,5 | -12,7 | -11,4 |
| beta alanine--pyruvate transaminase | WR30_RS23180 | BCAM1441 | 3,2 | 10,9 | 4,1 |
| putative methylmalonate-semialdehyde dehydrogenase | WR30_RS23185 | BCAM1442 | 2,3 | 7,9 | 2,3 |
| putative exported protein | WR30_RS23190 | BCAM1443 | 6,3 | 2,8 | 4,8 |
| two-component regulatory system, response regulator protein | WR30_RS23395 | BCAM1484 | 6,3 | 11,7 | 4,9 |
| ornithine cyclodeaminase | WR30_RS23400 | BCAM1485 | 4,1 | 1,3 | 4,8 |
| hypothetical protein | WR30_RS23425 | BCAM1490 | 5,5 | 3,9 | 36,3 |
| hypothetical protein | WR30_RS23430 | BCAM1491 | 1,0 | 1,1 | 6,1 |
| pseudo | WR30_RS23470 | BCAM1498 | -2,3 | -4,2 | -2,2 |
| putative universal stress protein | WR30_RS23475 | BCAM1500 | 27,1 | -1,4 | 14,1 |
| conserved hypothetical protein | WR30_RS23480 | BCAM1501 | 9,4 | 4,3 | 7,0 |
| Protein of unknown function (DUF3562) | WR30_RS23485 | BCAM1502 | 8,1 | 2,5 | 8,7 |
| glyoxalase/bleomycin resistanceprotein/dioxygenase superfamily protein | WR30_RS23985 | BCAM1547 | 3,7 | 5,6 | 6,0 |
| putative peptidoglycan-associated lipoprotein | WR30_RS24035 | BCAM1550 | 10,6 | 2,5 | 2,8 |
| recombination associated protein RdgC | WR30_RS13285 | BCAM1558 | -4,4 | -2,0 | -1,1 |
| putative BNR/Asp-box protein | WR30_RS24140 | BCAM1569 | 42,2 | 1,8 | 14,7 |
| phosphoenolpyruvate carboxykinase PckG | WR30_RS24220 | BCAM1581 | 5,6 | -1,2 | 2,4 |
| 3-alpha-(or 20-beta)-hydroxysteroid dehydrogenase | WR30_RS24225 | BCAM1582 | 11,4 | 7,2 | 4,4 |
| hypothetical protein | WR30_RS24380 | BCAM1602 | 4,4 | 1,7 | 3,4 |
| hypothetical protein | WR30_RS24455 | BCAM1617 | 1,5 | 6,1 | 1,4 |
| translation initiation factor IF-1 InfA2 | WR30_RS24480 | BCAM1618 | 1,7 | 2,3 | 3,8 |
| putative cyclic-di-GMP signaling protein | WR30_RS24745 | BCAM1670 | -1,2 | -1,8 | -3,8 |
| putative nitrite/sulfite reductase | WR30_RS24780 | BCAM1676 | -14,0 | -2,3 | -6,2 |
| putative fumarylpyruvate hydrolase | WR30_RS24870 | BCAM1692 | -2,2 | -2,9 | -10,6 |
| conserved hypothetical protein | WR30_RS24875 | BCAM1693 | -4,5 | -4,9 | -10,1 |
| Major Facilitator Superfamily protein | WR30_RS24880 | BCAM1694 | -10,1 | -4,9 | -16,1 |
| MarC family protein | WR30_RS24930 | BCAM1709 | -2,6 | -3,4 | -0,5 |
| putative enoyl-CoA hydratase/isomerase | WR30_RS24935 | BCAM1710 | -1.3 | -10.1 | 4.9 |
| phenylacetate-coenzyme A ligase | WR30_RS24940 | BCAM1711 | -2.2 | -10.9 | 11.0 |
| 3-hydroxy-acyl-CoA dehydrogenase | WR30_RS24945 | BCAM1712 | -1.7 | -12.6 | 10.7 |
| LysR family regulatory protein | WR30_RS25005 | BCAM1722 | -2,4 | -1,8 | -3,3 |
| family S9 serine peptidase | WR30_RS25115 | BCAM1744 | 12,0 | 23,9 | 91,8 |
| putative magnesium-transporting ATPase | WR30_RS25120 | BCAM1745 | -3,7 | -3,0 | -2,6 |
| putative DNA-binding protein | WR30_RS25130 | BCAM1746 | -14,1 | -15,6 | -5,9 |
| LysR family regulatory protein | WR30_RS25135 | BCAM1747 | -25,3 | -14,8 | -10,6 |
| putative sulfate transporter | WR30_RS25180 | BCAM1753A | -2,2 | -1,3 | -4,3 |
| putative mechanosensitive ion channel | WR30_RS25185 | BCAM1754 | -5,5 | -1,9 | -1,8 |
| GntR family regulatory protein | WR30_RS25190 | BCAM1755 | -4,5 | -2,2 | -2,3 |
| putative molybdopterin oxidoreductase | WR30_RS25195 | BCAM1756 | -3,1 | -3,5 | -1,4 |
| putative solute-binding protein | WR30_RS25230 | BCAM1762 | -6,6 | -2,6 | -4,3 |
| putative regulatory protein HipA | WR30_RS25255 | BCAM1766 | -3,8 | -3,4 | -5,5 |
| putative regulatory protein HipB | WR30_RS25260 | BCAM1767 | -3,0 | -3,5 | -4,1 |
| peptidoglycan-binding lysm:peptidase m23b precursor | WR30_RS25340 | BCAM1780 | -3,8 | 1,7 | 4,5 |
| hypothetical protein | WR30_RS25515 | BCAM1803 | 3,2 | 1,9 | 8,8 |
| putative universal stress protein | WR30_RS25665 | BCAM1829 | 35,8 | 1,2 | 22,0 |
| putative acetyltransferase | WR30_RS25700 | BCAM1840 | -3,8 | -2,4 | -1,7 |
| hypothetical protein | WR30_RS25750 | BCAM1850 | 1,6 | 3,9 | 1,1 |
| short chain dehydrogenase | WR30_RS25755 | BCAM1851 | -2,0 | 3,4 | 2,7 |
| [2Fe-2S]-binding protein | WR30_RS25760 | BCAM1852 | -2,3 | -2,0 | -5,1 |
| conserved hypothetical protein | WR30_RS25795 | BCAM1858 | 6,6 | 1,9 | 3,5 |
| putative transcription elongation factor | WR30_RS26195 | BCAM1928 | -4,0 | -1,4 | 1,4 |
| LuxR superfamily regulatory protein | WR30_RS26220 | BCAM1934 | 1,1 | -1,1 | -7,2 |
| putative isochorismatase | WR30_RS26230 | BCAM1936 | -1,4 | -1,6 | -6,1 |
| Major Facilitator Superfamily protein | WR30_RS26235 | BCAM1937 | 1,1 | -1,1 | -37,8 |
| ArsR family regulatory protein | WR30_RS26465 | BCAM1966 | -5,2 | -1,4 | -2,0 |
| putative xenobiotic reductase XenB | WR30_RS26470 | BCAM1967 | -2,4 | -1,5 | -3,2 |
| hypothetical protein | WR30_RS26540 | BCAM1978 | 2,3 | 3,0 | 3,7 |
| hypothetical protein | WR30_RS26665 | BCAM2002 | 4,9 | 2,6 | 1,0 |
| aspartate carbamoyltransferase PyrB | WR30_RS26690 | BCAM2006 | 9,7 | 24,8 | 11,5 |
| TonB-dependent siderophore receptor | WR30_RS26705 | BCAM2007 | -11,3 | -6,0 | -1,1 |
| hypothetical protein | WR30_RS26710 | BCAM2008 | -9,1 | -1,1 | -1,5 |
| 2OG-Fe(II) oxygenase superfamily protein | WR30_RS26715 | BCAM2009 | -51,3 | -10,0 | -1,6 |
| conserved hypothetical protein | WR30_RS26720 | BCAM2010 | -6,9 | -1,4 | -1,2 |
| LuxR superfamily regulatory protein | WR30_RS26795 | BCAM2022 | 2,8 | 2,8 | 6,8 |
| hypothetical protein | WR30_RS26800 | BCAM2023 | 1,0 | -1,7 | 6,0 |
| putative membrane protein | WR30_RS26805 | BCAM2024 | 24,9 | 4,5 | 27,9 |
| putative periplasmic trehalase precursor TreA | WR30_RS26980 | BCAM2064 | 1,9 | 5,6 | 2,2 |
| putative undecaprenyl pyrophosphate synthetase UppS | WR30_RS26990 | BCAM2067 | 3,9 | 5,4 | 2,9 |
| hypothetical protein | WR30_RS26995 | BCAM2068 | 2,5 | 10,1 | 2,8 |
| putative exported protein | WR30_RS27020 | BCAM2073 | -10,1 | 1,4 | -1,9 |
| diaminopimelate decarboxylase LysA | WR30_RS27035 | BCAM2076 | 1,5 | -3,1 | -1,9 |
| hypothetical protein | WR30_RS27090 | BCAM2081 | 13,0 | 2,3 | 3,5 |
| putative membrane protein | WR30_RS27130 | BCAM2085 | -1,9 | -2,7 | -4,3 |
| putative spermidine synthase | WR30_RS27135 | BCAM2086 | -1,9 | -1,8 | -5,1 |
| putative lipoprotein | WR30_RS27140 | BCAM2087 | -2,3 | -1,5 | -10,6 |
| putative gamma-glutamylputrescine synthetase PuuA | WR30_RS27185 | BCAM2094 | 3,0 | 6,1 | -17,3 |
| putative DNA-binding protein | WR30_RS27190 | BCAM2095 | 1,5 | 2,3 | -15,3 |
| putative gamma-glutamylputrescine reductase PuuB | WR30_RS27195 | BCAM2096 | 2,6 | 3,4 | -76,6 |
| hypothetical protein | WR30_RS27295 | BCAM2154 | 3,6 | 3,2 | 3,9 |
| hypothetical protein | WR30_RS27320 | BCAM2159 | 4,4 | -4,3 | 1,7 |
| conserved hypothetical protein | WR30_RS27350 | BCAM2167 | 12,0 | 1,7 | 6,6 |
| putative outer membrane autotransporter | WR30_RS27360 | BCAM2169 | 13,5 | 21,0 | 65,8 |
| hypothetical protein | WR30_RS27365 | BCAM2170 | 4,1 | 1,2 | 2,5 |
| rod shape-determining protein MreB | WR30_RS27370 | BCAM2171 | 8,9 | 6,8 | 12,2 |
| enoyl-CoA hydratase/isomerase family | WR30_RS27640 | BCAM2191 | 3,3 | -2,8 | -13,2 |
| enoyl-CoA hydratase | WR30_RS27645 | BCAM2192 | 3,3 | -1,6 | -7,7 |
| putative 3-hydroxyisobutyrate dehydrogenase MmsB | WR30_RS27650 | BCAM2193 | 3,5 | -5,0 | -10,1 |
| methylmalonate-semialdehyde dehydrogenase MmsA | WR30_RS27655 | BCAM2194 | 3,9 | -8,9 | -4,7 |
| putative AMP-binding enzyme | WR30_RS27660 | BCAM2195 | 4,3 | -7,0 | -4,3 |
| putative acyl-CoA dehydrogenase | WR30_RS27665 | BCAM2196 | 2,9 | -7,7 | -6,4 |
| hypothetical protein | WR30_RS27720 | BCAM2207 | 2,7 | 3,5 | 3,7 |
| conserved hypothetical protein | WR30_RS27735 | BCAM2209 | 24,4 | 2,1 | 5,2 |
| putative membrane protein | WR30_RS27740 | BCAM2210 | 26,9 | 4,0 | 6,0 |
| 2Fe-2S iron-sulfur | WR30_RS27750 | BCAM2212 | 1,1 | 1,9 | -7,2 |
| glyoxylase/bleomycin resistance protein/dioxygenase family protein | WR30_RS27835 | BCAM2242 | -1,6 | -3,9 | -4,0 |
| putative amino acid ABC transporter ATP-binding protein | WR30_RS27860 | BCAM2247 | 2,8 | 4,4 | -1,2 |
| putative methylamine dehydrogenase | WR30_RS28040 | BCAM2301 | 1,5 | 5,7 | 8,8 |
| putative leucyl aminopeptidase precursor | WR30_RS33505 | BCAM2308 | 4,3 | 12,0 | 15,9 |
| major facilitator superfamily protein | WR30_RS33530 | BCAM2310 | -7,3 | -3,4 | -2,6 |
| putative outer membrane porin protein | WR30_RS33560 | BCAM2311 | 1,0 | 4,2 | 1,7 |
| hypothetical protein (ProX; ABC-type proline/glycine betaine transport systems, periplasmic components) | WR30_RS33600 | BCAM2317 | 2,0 | 27,3 | 9,6 |
| putative ferredoxin oxidoreductase | WR30_RS33605 | BCAM2318 | 1,6 | 19,3 | 6,4 |
| iron-sulphur Rieske protein | WR30_RS33610 | BCAM2319 | 1,4 | 24,1 | 11,3 |
| putative electron transfer flavoprotein subunit alpha EtfA | WR30_RS33620 | BCAM2321 | 2,0 | 38,6 | 11,3 |
| putative iron-sulfur cluster membrane protein | WR30_RS33625 | BCAM2322 | 1,5 | 19,2 | 3,2 |
| putative N-methylproline demethylase | WR30_RS33630 | BCAM2323 | -1,3 | 16,8 | 8,0 |
| hypothetical protein | WR30_RS33635 | BCAM2324 | 2,1 | 30,9 | 17,0 |
| putative dipeptidase | WR30_RS33640 | BCAM2325 | 1,6 | 30,7 | 15,8 |
| serine hydroxymethyltransferase GlyA | WR30_RS33645 | BCAM2326 | 1,7 | 31,3 | 12,7 |
| AraC family regulatory protein | WR30_RS33650 | BCAM2327 | 1,8 | 4,3 | 2,0 |
| hypothetical protein | WR30_RS33655 | BCAM2328 | 2,8 | 4,6 | 9,6 |
| putative glutathione-independent formaldehyde dehydrogenase FdhA | WR30_RS33680 | BCAM2333 | 2,4 | 63,6 | 11,4 |
| conserved hypothetical protein | WR30_RS33750 | BCAM2344 | -1,3 | 8,7 | -2,5 |
| hypothetical protein | WR30_RS33755 | BCAM2345 | 1,0 | 76,6 | 1,4 |
| putative OmpW-family exported protein | WR30_RS33760 | BCAM2346 | -1,5 | 6,6 | -1,3 |
| putative lipoprotein | WR30_RS33765 | BCAM2347 | 1,5 | 22,5 | -1,9 |
| putative lipoprotein | WR30_RS33770 | BCAM2348 | 5,3 | 12,0 | 2,4 |
| putative ABC transporter transmembrane protein | WR30_RS33785 | BCAM2351 | 2,3 | 3,6 | -1,1 |
| putative signal-transduction and transcriptional regulator Fis and NtrC family protein | WR30_RS33890 | BCAM2365 | 4,6 | 1,9 | 2,5 |
| penicillin-binding protein (fragment) | WR30_RS33940 | BCAM2375 | 2,9 | 3,5 | 1,6 |
| permease | WR30_RS33950 | BCAM2376 | 1,2 | 1,5 | 5,4 |
| hypothetical protein | WR30_RS33955 | BCAM2377 | 8,5 | 9,4 | 68,1 |
| x-prolyl-dipeptidyl aminopeptidase PepX | WR30_RS33960 | BCAM2378 | 7,3 | 58,9 | 72,0 |
| putative ABC transporter system permease | WR30_RS33985 | BCAM2382 | -1,9 | 3,1 | -4,1 |
| ABC transporter ATP-binding protein | WR30_RS33995 | BCAM2384 | 1,0 | 2,4 | -4,2 |
| rifampin ADP-ribosyl transferase Arr | WR30_RS34000 | BCAM2385 | -3,1 | -1,8 | -2,4 |
| putative SLT transglycosylase | WR30_RS34005 | BCAM2386 | 4,0 | 2,0 | -1,1 |
| hypothetical protein | WR30_RS34130 | BCAM2400b | 1,7 | 1,1 | -6,5 |
| putative glycine betaine/L-proline ABC transporter substrate-binding protein | WR30_RS34175 | BCAM2407 | 2,3 | 50,6 | 4,9 |
| AraC family regulatory protein | WR30_RS34180 | BCAM2408 | 2,1 | 45,9 | 5,2 |
| putative glycine-betaine ABC transporter permease | WR30_RS34185 | BCAM2409 | 1,8 | 15,2 | 2,5 |
| putative glycine betaine/L-proline ABC transporter ATP-binding protein | WR30_RS34190 | BCAM2410 | 1,1 | 21,4 | 3,7 |
| hypothetical protein | WR30_RS34200 | BCAM2411 | 2,3 | 4,4 | 1,3 |
| hypothetical protein | WR30_RS34335 | BCAM2425 | 2,0 | 3,1 | 15,6 |
| putative diguanylate phosphodiesterase | WR30_RS34340 | BCAM2426 | 1,7 | 3,3 | 4,3 |
| putative phospholipase C | WR30_RS34355 | BCAM2429 | 1,0 | -5,5 | -1,6 |
| putative biotin carboxylase | WR30_RS34365 | BCAM2430 | 1,3 | -14,2 | -1,7 |
| enoyl-CoA hydratase | WR30_RS34370 | BCAM2431 | 1,7 | -6,0 | 1,0 |
| putative biotin-dependent carboxyl transferase | WR30_RS34375 | BCAM2432 | 1,1 | -32,9 | -1,2 |
| putative acyl-CoA dehydrogenase | WR30_RS34380 | BCAM2433 | 3,0 | -28,4 | 1,2 |
| TetR family regulatory protein | WR30_RS34385 | BCAM2434 | 1,0 | -9,3 | -2,3 |
| putative exported protein | WR30_RS34445 | BCAM2440 | -16,3 | 1,0 | -21,3 |
| putative solute-binding protein precursor | WR30_RS34450 | BCAM2441 | -16,7 | 1,7 | -50,0 |
| putative cytochrome c | WR30_RS34460 | BCAM2443 | -1,7 | 1,4 | -8,3 |
| hypothetical protein | WR30_RS34565 | BCAM2463 | 2,8 | -2,4 | 4,7 |
| putative transport membrane protein | WR30_RS34585 | BCAM2465 | -3,2 | -1,8 | -1,3 |
| putative aldehyde dehydrogenase family protein | WR30_RS34590 | BCAM2468 | -14,4 | -7,2 | -1,3 |
| DeoR family regulatory protein | WR30_RS34620 | BCAM2473 | 3,6 | 1,5 | 1,9 |
| LysR family regulatory protein | WR30_RS34660 | BCAM2481 | -2,0 | -3,6 | -2,0 |
| agmatinase SpeB | WR30_RS34665 | BCAM2482 | -2,5 | -5,5 | -2,5 |
| Major Facilitator Superfamily protein | WR30_RS34670 | BCAM2483 | -47,5 | -97,7 | -35,3 |
| 5-methyltetrahydropteroyltriglutamate--homocysteine methyltransferase MetE | WR30_RS34680 | BCAM2484 | -109,1 | -362,0 | -109,1 |
| conserved hypothetical protein | WR30_RS15000 | BCAM2492 | -2,8 | -1,6 | -5,7 |
| putative NADP-dependent glyceraldehyde-3-phosphate dehydrogenase | WR30_RS15005 | BCAM2493 | -2,1 | -3,5 | -4,2 |
| putative phosphonoacetate hydrolase PhnA | WR30_RS15010 | BCAM2494 | -1,4 | -3,1 | -4,7 |
| binding-protein-dependent transport system protein | WR30_RS15020 | BCAM2496 | -3,5 | -2,7 | -5,1 |
| ABC transporter ATP-binding protein | WR30_RS15025 | BCAM2497 | -1,2 | 1,0 | -9,4 |
| extracellular solute-binding protein | WR30_RS15030 | BCAM2498 | -1,7 | -4,5 | -5,0 |
| 2-aminoethylphosphonate--pyruvate transaminase | WR30_RS15035 | BCAM2499 | -1,2 | -7,2 | -3,8 |
| conserved hypothetical protein | WR30_RS15060 | BCAM2504 | -8,5 | -7,7 | -5,5 |
| putative extracellular ligand-binding receptor | WR30_RS15070 | BCAM2506 | 12,1 | -2,4 | 1,1 |
| dihydroorotase PyrC | WR30_RS15150 | BCAM2521 | 1,3 | -3,2 | -1,5 |
| putative hexapeptide repeat protein | WR30_RS15155 | BCAM2522 | 1,1 | -3,9 | -1,7 |
| hypothetical protein | WR30_RS15160 | BCAM2523 | 1,0 | -5,9 | -1,1 |
| putative alpha-beta hydrolase | WR30_RS15230 | BCAM2536 | -2,5 | -1,6 | -3,3 |
| multidrug efflux system outer membrane protein OpcM | WR30_RS15250 | BCAM2549 | 4,5 | 54,6 | 3,0 |
| multidrug efflux system transporter protein CeoB | WR30_RS15255 | BCAM2550 | 3,2 | 30,5 | 5,4 |
| multidrug efflux system transport protein CeoA | WR30_RS15260 | BCAM2551 | 3,3 | 40,8 | 4,8 |
| putative hydrolase LlpE | WR30_RS15265 | BCAM2552 | 1,3 | 10,5 | 4,3 |
| putative 4-aminobutyrate aminotransferase | WR30_RS15305 | BCAM2561 | 2,3 | 2,0 | -6,0 |
| putative beta-ketoadipyl CoA thiolase | WR30_RS15390 | BCAM2568 | -10,3 | -14,3 | -14,1 |
| IclR family regulatory protein | WR30_RS15395 | BCAM2569 | -30,1 | -29,0 | -50,2 |
| putative carboxymuconolactone dehydrogenase family protein | WR30_RS28085 | BCAM2595 | -1,8 | -3,0 | -2,9 |
| putative hemin transport system, substrate-binding protein HmuT | WR30_RS28215 | BCAM2628 | -1,4 | 3,1 | 2,7 |
| putative membrane protein | WR30_RS28230 | BCAM2631 | -8,6 | 1,2 | 1,0 |
| putative methyltransferase | WR30_RS28260 | BCAM2640 | 4,3 | 1,9 | 1,7 |
| hypothetical protein | WR30_RS28295 | BCAM2647 | 7,8 | -2,3 | -2,0 |
| ArsR family regulatory protein | WR30_RS28345 | BCAM2655 | 5,3 | 3,3 | 2,1 |
| putative amidohydrolase | WR30_RS28360 | BCAM2658 | 2,1 | 1,7 | 3,8 |
| IclR family regulatory protein | WR30_RS28380 | BCAM2662 | -3,5 | -1,1 | -7,2 |
| DsbA-like thioredoxin protein | WR30_RS28385 | BCAM2663 | -5,8 | -3,7 | -7,7 |
| LysR family regulatory protein | WR30_RS28390 | BCAM2664 | -14,1 | -6,2 | -6,0 |
| putative low-specificity L-threonine aldolase LtaE | WR30_RS28430 | BCAM2671 | -2,4 | 1,4 | -4,0 |
| putative cytochrome oxidase subunit I | WR30_RS28445 | BCAM2674 | 1,1 | -8,1 | 1,5 |
| hypothetical protein | WR30_RS28520 | BCAM2680 | 3,1 | 4,4 | 2,5 |
| putative acetyltransferase | WR30_RS28540 | BCAM2684 | 3,8 | 1,0 | 1,5 |
| putative membrane protein | WR30_RS28615 | BCAM2700 | 1,1 | -8,1 | -5,5 |
| aconitate hydratase 1 AcnA | WR30_RS28620 | BCAM2701 | -1,3 | -8,3 | -5,7 |
| 2-methylcitrate synthase PrpC | WR30_RS28625 | BCAM2702 | -1,4 | -9,2 | -3,7 |
| probable methylisocitrate lyase PrpB | WR30_RS28630 | BCAM2703 | -1,5 | -10,3 | -4,0 |
| hypothetical protein | WR30_RS28665 | BCAM2709 | -2,0 | -4,3 | -1,8 |
| putative acetyl-CoA synthetase | WR30_RS28670 | BCAM2710 | 5,4 | 1,3 | 2,9 |
| putative exported protein | WR30_RS28685 | BCAM2713 | -1,2 | -3,7 | -4,4 |
| putative sigma-54 dependent transcriptional regulator | WR30_RS28690 | BCAM2715 | -2,5 | -2,7 | -3,8 |
| putative citrate transporter | WR30_RS28695 | BCAM2716 | 4,9 | -2,3 | -2,2 |
| putative outer membrane porin protein | WR30_RS28735 | BCAM2723 | 1,4 | 1,5 | 12,0 |
| putative oligopeptide ABC transporter ATP-binding protein OppD | WR30_RS28745 | BCAM2725 | 1,0 | 1,3 | 4,0 |
| putative ABC transporter system permease OppC | WR30_RS28750 | BCAM2726 | 1,1 | 1,2 | 7,5 |
| putative oligopeptide transporter permease OppB | WR30_RS28755 | BCAM2727 | -1,7 | 1,2 | 4,9 |
| putative periplasmic oligopeptide-binding protein precursor OppA | WR30_RS28760 | BCAM2728 | -1,6 | -1,3 | 5,3 |
| putative tripeptide permease | WR30_RS28770 | BCAM2730 | 1,1 | -3,4 | 2,4 |
| MoaA/NifB/PqqE family protein | WR30_RS28815 | BCAM2739 | -3,4 | -2,3 | 1,1 |
| conserved hypothetical protein | WR30_RS28835 | BCAM2742 | 9,1 | 1,6 | 13,1 |
| hypothetical protein (Protein of unknown function (DUF466)) | WR30_RS28855 | BCAM2745 | 38,3 | 49,2 | 3,3 |
| carbon starvation protein A CstA | WR30_RS28860 | BCAM2746 | 22,3 | 27,5 | 2,8 |
| putative exported protein | WR30_RS28900 | BCAM2755 | 6,7 | -1,1 | 2,6 |
| putative dioxygenase | WR30_RS28955 | BCAM2775 | 2,6 | 9,3 | -1,9 |
| putative exported cyclase | WR30_RS28975 | BCAM2778 | -1,6 | -1,8 | -3,1 |
| aminotransferase | WR30_RS29000 | BCAM2784 | -2,1 | -1,2 | -3,8 |
| putative diguanylate cyclase | WR30_RS29175 | BCAM2822 | 4,0 | 2,3 | 1,5 |
| squalene--hopene cyclase Shc | WR30_RS29220 | BCAM2831 | -4,9 | -3,3 | -4,6 |
| putative flavin containing amine oxidase | WR30_RS29225 | BCAM2832 | -3,2 | -2,5 | -3,6 |
| hypothetical protein | WR30_RS29235 | BCAM2834 | 4,0 | 3,4 | 1,8 |
| putative esterase | WR30_RS29240 | BCAM2835 | 2,1 | 1,6 | 3,2 |
| putative diguanylate cyclase | WR30_RS29245 | BCAM2836 | 3,9 | 1,6 | 18,1 |
| putative fusaric acid resistance transporter protein | WR30_RS20650 | BCAS0016 | -5,1 | -2,6 | -2,9 |
| MarR family regulatory protein | WR30_RS20640 | BCAS0018 | 1,4 | 1,1 | -3,0 |
| putative oxidoreductase | WR30_RS20580 | BCAS0058 | -32,7 | -4,9 | -1,5 |
| dihydrodipicolinate synthetase family protein | WR30_RS20410 | BCAS0102 | -9,2 | -1,9 | 1,3 |
| putative putrescine-binding periplasmic protein precursor | WR30_RS20315 | BCAS0124 | 1,2 | 13,4 | -1,6 |
| hypothetical protein | WR30_RS20070 | BCAS0151 | 17,4 | 21,3 | 56,5 |
| putative hydrolase | WR30_RS20065 | BCAS0152 | 1,6 | 1,5 | 9,6 |
| hypothetical protein | WR30_RS19750 | BCAS0194 | -1,8 | -3,9 | -2,9 |
| putative transporter protein-Dct family | WR30_RS19715 | BCAS0199 | 1,5 | 1,1 | -4,4 |
| putative FAD dependent oxidoreductase | WR30_RS19705 | BCAS0201 | 1.3 | 2.5 | -45.9 |
| hypothetical protein | WR30_RS19700 | BCAS0202 | -1.9 | -2.1 | -10.6 |
| ABC transporter protein | WR30_RS19695 | BCAS0203 | -1.7 | 1.3 | -26.4 |
| ABC transporter ATP-binding protein | WR30_RS19690 | BCAS0204 | -1.5 | 1.6 | -142.9 |
| TauD/TfdA taurine catabolism dioxygenase family protein | WR30_RS19685 | BCAS0205 | -1.7 | -2.6 | -25.3 |
| putative methyltransferase family protein | WR30_RS19680 | BCAS0206 | -1.2 | -1.9 | -89.3 |
| hypothetical protein | WR30_RS19675 | BCAS0207 | 1.1 | -1.2 | -52.4 |
| putative acyl-CoA dehydrogenase | WR30_RS19670 | BCAS0208 | -1.3 | -1.9 | -159.8 |
| conserved hypothetical protein | WR30_RS19665 | BCAS0209 | -1.3 | 5.2 | -130.7 |
| putative AMP-binding enzyme | WR30_RS19660 | BCAS0210 | -0.5 | -1.5 | -18.4 |
| putative pyridoxal-dependent decarboxylase | WR30_RS19655 | BCAS0211 | -1.0 | -1.6 | -47.2 |
| conserved hypothetical protein | WR30_RS19650 | BCAS0212 | -0.8 | 2.4 | -13.5 |
| hypothetical protein | WR30_RS19645 | BCAS0213 | -1.7 | -6.5 | -149.1 |
| hypothetical protein | WR30_RS19640 | BCAS0214 | -1.6 | 2.8 | -217.0 |
| putative acyl carrier protein | WR30_RS19630 | BCAS0216 | -2.0 | -3.5 | -229.1 |
| hypothetical protein | WR30_RS19625 | BCAS0217 | -2.2 | -2.6 | -230.7 |
| hypothetical protein | WR30_RS19620 | BCAS0218 | -2.0 | -3.5 | -132.5 |
| putative exported protein | WR30_RS19615 | BCAS0219 | 1.1 | -3.9 | -324.0 |
| putative permease | WR30_RS19610 | BCAS0220 | 1.6 | -1.5 | -98.4 |
| ABC transporter ATP-binding protein | WR30_RS19605 | BCAS0221 | 2.3 | -1.4 | -238.9 |
| putative AMP-dependent synthetase | WR30_RS19600 | BCAS0222 | 1.2 | -1.6 | -166.6 |
| putative fatty acid desaturase | WR30_RS19595 | BCAS0223 | -1.1 | -2.5 | -173.6 |
| conserved hypothetical protein | WR30_RS01490 | BCAS0224 | -1.2 | -3.6 | -206.5 |
| LysR family regulatory protein | WR30_RS19585 | BCAS0225 | -1.1 | -1.7 | -6.6 |
| putative hydrolase | WR30_RS19580 | BCAS0226 | -13.3 | -9.4 | -8.7 |
| putative proline/betaine transporter | WR30_RS19575 | BCAS0227 | -14.2 | -17.8 | -5.3 |
| hybrid two-component system kinase-response regulator protein | WR30_RS19545 | BCAS0234 | -3,5 | -1,1 | -4,3 |
| hypothetical protein | WR30_RS19510 | BCAS0238 | 11,6 | 1,0 | 6,1 |
| hypothetical protein | WR30_RS19405 | BCAS0242 | -4,0 | -4,2 | -2,8 |
| GntR family regulatory protein | WR30_RS19360 | BCAS0258 | 4,3 | 3,5 | 3,2 |
| putative acetyltransferase | WR30_RS19280 | BCAS0262 | 1,6 | 3,0 | 4,6 |
| two-component regulatory system, response regulator protein | WR30_RS19275 | BCAS0263 | 2,6 | 10,4 | 10,1 |
| two-component regulatory system, sensor kinase protein | WR30_RS19270 | BCAS0264 | 1,8 | 7,3 | 15,0 |
| subfamily S9C non-peptidase homologue | WR30_RS19265 | BCAS0265 | 3,6 | 11,9 | 4,6 |
| periplasmic solute-binding protein | WR30_RS19215 | BCAS0291 | 15,5 | 15,0 | 12,2 |
| hypothetical protein | WR30_RS19205 | BCAS0292 | 25,8 | 31,6 | 18,1 |
| putative GtrA-like family protein | WR30_RS19195 | BCAS0294 | 4,7 | 1,4 | 1,2 |
| glycosyltransferase | WR30_RS19190 | BCAS0295 | 3,3 | 1,7 | 2,5 |
| hypothetical protein | WR30_RS19085 | BCAS0313 | 1,0 | 3,4 | 3,4 |
| putative aldose 1-epimerase | WR30_RS16055 | BCAS0353 | 1,3 | 3,5 | -1,5 |
| AraC family regulatory protein | WR30_RS16060 | BCAS0354 | -1,3 | 4,4 | -2,2 |
| xylose isomerase XylA | WR30_RS16065 | BCAS0355 | 1,1 | 18,4 | -2,1 |
| D-xylose-binding periplasmic protein precursor XylF | WR30_RS16075 | BCAS0356 | 1,2 | 32,4 | -1,8 |
| xylose transporter ATP-binding subunit XylG | WR30_RS16080 | BCAS0357 | -2,0 | 38,1 | -1,8 |
| xylose transport system permease protein XylH | WR30_RS16085 | BCAS0358 | -1,5 | 21,7 | -1,9 |
| putative periplasmic solute-binding protein | WR30_RS16375 | BCAS0388 | 1,6 | 3,3 | 3,4 |
| putative diguanylate cyclase | WR30_RS16240 | BCAS0398 | 2,5 | -1,2 | 8,8 |
| OsmC family protein | WR30_RS19930 | BCAS0437 | -1,5 | 4,2 | 5,1 |
| putative acetyltransferase-GNAT family | WR30_RS18200 | BCAS0468 | -11,6 | -2,8 | -2,1 |
| conserved hypothetical protein | WR30_RS18195 | BCAS0469 | -8,7 | -4,0 | -3,8 |
| two-component regulatory system, response regulator protein | WR30_RS18190 | BCAS0470 | -6,1 | 1,0 | 1,0 |
| putative endoribonuclease L-psp family protein | WR30_RS18170 | BCAS0492 | 2,3 | 11,7 | 1,3 |
| 1,6-dihydroxycyclohexa-2,4-diene-1-carboxylate dehydrogenase BenD | WR30_RS18165 | BCAS0493 | 1,5 | 11,3 | -2,4 |
| benzoate 1,2-dioxygenase electron transfer component BenC | WR30_RS18160 | BCAS0494 | 1,0 | 8,5 | -1,6 |
| benzoate 1,2-dioxygenase beta subunit BenB | WR30_RS18155 | BCAS0495 | 1,2 | 6,3 | -1,5 |
| benzoate 1,2-dioxygenase alpha subunit BenA | WR30_RS18150 | BCAS0496 | 1,2 | 8,6 | -1,2 |
| putative transporter-NRAMP family | WR30_RS15680 | BCAS0630 | -7,2 | -7,5 | -2,6 |
| 60 kDa chaperonin 3 GroEL3 | WR30_RS18960 | BCAS0637 | -13,5 | -2,2 | -1,4 |
| 10 kDa chaperonin 3 GroES3 | WR30_RS18965 | BCAS0638 | -13,4 | -2,8 | -1,6 |
| putative Na+ dependent nucleoside transporter family protein | WR30_RS21000 | BCAS0730 | -1,7 | 1,0 | -3,1 |
| phenylhydantoinase Dht | WR30_RS20995 | BCAS0731 | -1,1 | 4,0 | -2,8 |
| putative cytosine/purines, uracil, thiamine, allantoin permease | WR30_RS20990 | BCAS0732 | -1,4 | 2,6 | -3,2 |
| dihydropyrimidine dehydrogenase | WR30_RS20985 | BCAS0733 | 1,6 | 4,9 | -2,9 |
| putative oxidoreductase | WR30_RS20980 | BCAS0734 | 1,1 | 3,9 | -2,8 |
| allantoate amidohydrolase | WR30_RS20975 | BCAS0735 | 5,3 | 3,6 | -1,6 |
| putative short-chain dehydrogenase family protein | WR30_RS20960 | BCAS0738 | 2,8 | -6,1 | -1,6 |
| putative acetyl-CoA synthetase | WR30_RS20955 | BCAS0739 | 5,5 | -3,6 | -1,6 |
| putative peptidoglycan-binding membrane protein | WR30_RS26880 | BCAS0759 | 3,6 | 2,3 | -1,7 |
| methyltransferase type 12 | WR30_RS33965 | Bcen2424_6510 | 3,9 | 3,0 | 1,9 |
| MoeA domain-containing protein | WR30_RS31785 | Bcenmc03_4042 | 2,5 | 3,6 | 4,2 |
| permease for cytosine/purines uracil thiamine allantoin | WR30_RS17890 | Bcenmc03_4305 | -72,5 | -278,2 | -91,8 |
| transcriptional regulator, LysR family | WR30_RS20270 | Bcenmc03_5647 | -8,5 | -7,0 | -3,6 |
| short-chain dehydrogenase/reductase SDR | WR30_RS15690 | Bcenmc03_6137 | -59,7 | -44,9 | -30,5 |
| acriflavin resistance protein | WR30_RS15695 | Bcenmc03_6138 | -28,6 | -21,7 | -14,0 |
| RND family efflux transporter MFP subunit | WR30_RS15700 | Bcenmc03_6139 | -12,5 | -2,8 | -5,9 |
| RND efflux system, outer membrane lipoprotein, NodT family | WR30_RS15710 | Bcenmc03_6141 | -11,7 | -11,3 | -16,2 |
| diguanylate cyclase | WR30_RS16240 | Bcenmc03_6264 | 2,3 | 1,7 | 7,1 |
| transcriptional regulator, AraC family | WR30_RS17600 | Bcenmc03_7037 | -4,2 | -2,1 | -3,9 |
| RND efflux system, outer membrane lipoprotein, NodT family | WR30_RS03025 | Bcep1808_5402 | 3,2 | 52,3 | 4,8 |
| acriflavin resistance protein | WR30_RS31945 | Bcep1808_5403 | 3,7 | 54,6 | 1,6 |
| RND family efflux transporter MFP subunit | WR30_RS31940 | Bcep1808_5404 | 1,1 | 186,1 | 1,3 |
| hypothetical protein | WR30_RS31935 | Bcep1808_5405 | 1,6 | 21,7 | 1,1 |
| drug resistance transporter, Bcr/CflA subfamily | WR30_RS31930 | Bcep1808_5406 | -1,3 | 20,5 | -1,1 |
| hypothetical protein (putative thermostable hemolysin) | WR30_RS31925 | Bcep1808_5407 | 1,1 | 69,1 | 1,1 |
| AMP-dependent synthetase and ligase | WR30_RS31920 | Bcep1808_5408 | 1,1 | 56,9 | 1,2 |
| Taurine catabolism dioxygenase TauD/TfdA | WR30_RS31915 | Bcep1808_5409 | 1,0 | 68,1 | 1,4 |
| alpha/beta hydrolase | WR30_RS31900 | Bcep1808_5412 | 1,4 | 3,8 | 1,5 |
| FAD-dependent pyridine nucleotide-disulphide reductase | WR30_RS18670 | Bcep1808_5560 | 7,3 | 2,6 | 11,8 |
| amino acid ABC transporter substrate-binding protein, PAAT family | WR30_RS14240 | Bcep1808_6366 | 6,6 | 3,8 | 8,3 |
| glycosyl transferase family protein | WR30_RS07045 | Bcep18194_A3881 | 3,8 | 3,8 | 1,0 |
| transcriptional regulator NrdR | WR30_RS07125 | Bcep18194_A3896 | 1,2 | -5,2 | -1,3 |
| Tfp pilus assembly protein FimT-like | WR30_RS07130 | Bcep18194_A3897 | 4,7 | -1,9 | -2,1 |
| putative prepilin-type cleavage/methylation-like protein | WR30_RS07140 | Bcep18194_A3899 | 4,5 | -1,1 | -2,3 |
| hypothetical protein | WR30_RS07145 | Bcep18194_A3900 | 10,9 | -1,5 | -1,9 |
| Tfp pilus assembly protein PilE | WR30_RS07150 | Bcep18194_A3901 | -1,3 | 1,2 | -3,0 |
| hypothetical protein | WR30_RS11315 | Bcep18194_A4711 | 7,2 | -1,2 | 3,2 |
| Lipolytic enzyme, G-D-S-L | WR30_RS00900 | Bcep18194_A6375 | 8,0 | 2,0 | 15,7 |
| Tellurite resistance TerB | WR30_RS15345 | Bcep18194_B0272 | -7,8 | -1,5 | 1,1 |
| stress protein | WR30_RS15340 | Bcep18194_B0273 | -4,9 | -1,3 | 1,2 |
| stress protein | WR30_RS15335 | Bcep18194_B0274 | -4,6 | -1,8 | 1,4 |
| fructose-bisphosphate aldolase | WR30_RS33675 | Bcep18194_B0565 | -1,2 | 4,1 | 2,2 |
| cysteinyl-tRNA synthetase | WR30_RS33535 | Bcep18194_B0590 | -9,8 | -6,4 | -6,5 |
| transcriptional activator FtrA | WR30_RS27275 | Bcep18194_B0778 | 10,4 | -1,3 | 3,1 |
| arginine decarboxylase | WR30_RS27270 | Bcep18194_B0779 | 22,0 | -1,4 | 6,8 |
| arginine:agmatin antiporter | WR30_RS27265 | Bcep18194_B0780 | 14,2 | -1,2 | 3,9 |
| Histone-like nucleoid-structuring protein H-NS | WR30_RS25410 | Bcep18194_B1226 | -1,1 | -2,1 | -3,6 |
| hypothetical protein | WR30_RS25125 | Bcep18194_B1280 | -5,2 | -4,4 | -4,1 |
| peptidoglycan-binding LysM | WR30_RS24955 | Bcep18194_B1315 | 1,8 | 3,6 | -1,6 |
| transcriptional regulator, TetR family | WR30_RS22930 | Bcep18194_B1743 | 26,5 | 9,0 | 3,5 |
| HlyD family secretion protein | WR30_RS22925 | Bcep18194_B1744 | 30,7 | 5,2 | 2,9 |
| ABC transporter, ATPase subunit | WR30_RS22920 | Bcep18194_B1745 | 23,1 | 6,2 | 4,0 |
| ABC efflux pump, inner membrane subunit | WR30_RS22915 | Bcep18194_B1746 | 15,5 | 3,2 | 3,8 |
| RND efflux system, outer membrane lipoprotein, NodT family | WR30_RS22910 | Bcep18194_B1747 | 12,2 | 6,8 | 3,2 |
| putative porin | WR30_RS22905 | Bcep18194_B1748 | 2,0 | 3,9 | 2,7 |
| Phosphotransferase KptA/Tpt1 | WR30_RS22700 | Bcep18194_B1795 | 4,5 | -1,6 | -1,3 |
| glycosyl transferase, group 1 | WR30_RS22675 | Bcep18194_B1800 | 6,7 | -1,1 | 1,8 |
| alpha/beta hydrolase | WR30_RS22440 | Bcep18194_B1862 | -1,1 | -4,2 | 1,1 |
| family M55 metallopeptidase | WR30_RS22435 | Bcep18194_B1863 | -2,2 | -5,7 | -1,3 |
| Alpha-1,2-mannosidase, putative | WR30_RS21870 | Bcep18194_B1991 | 1,1 | 1,0 | -6,5 |
| type II secretion system protein | WR30_RS35015 | Bcep18194_B2053 | 5,5 | 10,1 | 3,4 |
| type II secretion system protein E | WR30_RS35020 | Bcep18194_B2054 | 4,7 | 11,9 | 5,2 |
| Peptidase A24A, prepilin type IV/Flp/Fap pilin component | WR30_RS35045 | Bcep18194_B2059 | 17,4 | 14,5 | 6,7 |
| transcriptional regulator, AraC family | WR30_RS33435 | Bcep18194_B2295 | -3,1 | -10,7 | -4,6 |
| acetate kinase | WR30_RS31970 | Bcep18194_B2633 | 4,3 | 2,6 | -1,1 |
| enoyl-ACP reductase | WR30_RS31955 | Bcep18194_B2636 | 4,9 | 2,1 | 1,1 |
| Transglycosylase-associated protein | WR30_RS31725 | Bcep18194_B2685 | -13,6 | -3,0 | 2,0 |
| Alpha-1,2-mannosidase, putative | WR30_RS30890 | Bcep18194_B2906 | -1,8 | -24,8 | -81,6 |
| Alpha-1,2-mannosidase, putative | WR30_RS30885 | Bcep18194_B2907 | -2,3 | -28,6 | -95,7 |
| monooxygenase component MmoB/DmpM | WR30_RS30145 | Bcep18194_B2969 | -1,1 | -3,0 | -2,5 |
| transcriptional regulator, MarR family | WR30_RS30610 | Bcep18194_C6567 | 8,7 | 7,2 | 4,7 |
| amino acid transporter | WR30_RS30615 | Bcep18194_C6568 | 4,6 | 1,9 | 2,7 |
| Cl- channel, voltage gated | WR30_RS30620 | Bcep18194_C6569 | 4,3 | 2,3 | 3,0 |
| CBS | WR30_RS30620 | Bcep18194_C6570 | 4,3 | 3,6 | 3,5 |
| OmpA/MotB family outer membrane protein | WR30_RS30630 | Bcep18194_C6571 | 2,2 | 3,0 | 8,8 |
| chitin-binding protein | WR30_RS16470 | Bcep18194_C6726 | 87,4 | 11,6 | 14,5 |
| glycosyl hydrolase chitinase | WR30_RS16475 | Bcep18194_C6728 | 19,8 | 12,5 | 32,7 |
| hypothetical protein | WR30_RS16480 | Bcep18194_C6731 | 4,4 | 5,9 | 17,1 |
| FAD dependent oxidoreductase | WR30_RS16810 | Bcep18194_C6735 | 14,0 | 2,5 | 13,7 |
| branched chain amino acid: 2-keto-4-methylthiobutyrate aminotransferase | WR30_RS16905 | Bcep18194_C6784 | -8,7 | -8,4 | -1,9 |
| aldehyde dehydrogenase | WR30_RS16910 | Bcep18194_C6785 | -3,7 | -3,8 | -3,1 |
| transcriptional regulator, TetR family | WR30_RS16915 | Bcep18194_C6786 | -12,6 | -6,9 | -4,4 |
| beta-hydroxyacid dehydrogenase | WR30_RS16925 | Bcep18194_C6789 | -6,6 | -10,6 | -8,3 |
| Phytanoyl-CoA dioxygenase | WR30_RS16930 | Bcep18194_C6790 | -6,8 | -11,6 | -10,6 |
| Major facilitator superfamily, (MFS_1) family | WR30_RS16935 | Bcep18194_C6791 | -2,5 | -3,6 | -3,5 |
| aminotransferase | WR30_RS16950 | Bcep18194_C6794 | -1,8 | -3,7 | -1,7 |
| collagenase | WR30_RS17220 | Bcep18194_C6876 | 2,6 | 5,5 | 1,9 |
| universal stress protein | WR30_RS17690 | Bcep18194_C6944 | 54,6 | 2,4 | 8,8 |
| hypothetical protein | WR30_RS17695 | Bcep18194_C6945 | 57,7 | 8,6 | 18,1 |
| TPR repeat-containing protein | WR30_RS17700 | Bcep18194_C6947 | 7,3 | 1,2 | 3,4 |
| thiamine S | WR30_RS17745 | Bcep18194_C6963 | -5,4 | -1,0 | -7,5 |
| Molybdopterin biosynthesis MoaE | WR30_RS17750 | Bcep18194_C6964 | -8,1 | -3,4 | -4,1 |
| GTP cyclohydrolase subunit MoaA | WR30_RS17755 | Bcep18194_C6965 | -5,9 | -1,5 | -2,8 |
| cytochrome bd ubiquinol oxidase, subunit I | WR30_RS17770 | Bcep18194_C6968 | -3,7 | -2,0 | -3,1 |
| cytochrome bd ubiquinol oxidase, subunit II | WR30_RS17775 | Bcep18194_C6969 | -4,3 | -1,6 | -3,8 |
| hypothetical protein | WR30_RS17780 | Bcep18194_C6970 | -4,4 | -2,1 | -3,0 |
| cyclic nucleotide-binding protein | WR30_RS17820 | Bcep18194_C6981 | 4,6 | 2,2 | 18,6 |
| sigma-24 (FecI-like) | WR30_RS17825 | Bcep18194_C6982 | 3,6 | 2,2 | 6,2 |
| putative tyrosinase | WR30_RS17845 | Bcep18194_C6986 | 2,9 | 30,1 | 22,9 |
| Phosphate-starvation-inducible protein E | WR30_RS17925 | Bcep18194_C7003 | -1,4 | -1,4 | -3,2 |
| membrane protein | WR30_RS17935 | Bcep18194_C7004 | -1,4 | -2,4 | -3,2 |
| hypothetical protein | WR30_RS17940 | Bcep18194_C7005 | -1,1 | -2,1 | -3,7 |
| transcriptional regulator, GntR family | WR30_RS17950 | Bcep18194_C7006 | -7,0 | -6,1 | -4,2 |
| formate dehydrogenase | WR30_RS17955 | Bcep18194_C7007 | -4,2 | -4,2 | -5,9 |
| FAD-dependent pyridine nucleotide-disulphide oxidoreductase | WR30_RS17960 | Bcep18194_C7008 | -6,1 | -1,8 | -2,1 |
| two component, sigma54 specific, transcriptional regulator, Fis family | WR30_RS17985 | Bcep18194_C7013 | -1,1 | -2,7 | -4,2 |
| PAS/PAC sensor hybrid histidine kinase | WR30_RS17990 | Bcep18194_C7014 | -1,3 | 11,0 | -1,4 |
| RNA polymerase factor sigma-54 | WR30_RS18010 | Bcep18194_C7019 | -14,7 | -1,2 | -2,7 |
| amidase-like protein | WR30_RS18175 | Bcep18194_C7051 | 48,8 | 2,9 | 25,6 |
| conserved hypothetical protein | WR30_RS18180 | Bcep18194_C7052 | 20,0 | 4,7 | 8,7 |
| response regulator receiver domain-containing protein | WR30_RS18260 | Bcep18194_C7079 | 3,7 | 1,8 | 3,4 |
| diguanylate cyclase/phosphodiesterase | WR30_RS18970 | Bcep18194_C7110 | 1,0 | 2,0 | 3,6 |
| diguanylate cyclase/phosphodiesterase | WR30_RS18975 | Bcep18194_C7111 | 1,2 | 3,0 | 2,6 |
| hypothetical protein | WR30_RS19285 | Bcep18194_C7225 | 1,6 | 2,1 | 4,7 |
| carbonate dehydratase | WR30_RS19410 | Bcep18194_C7283 | -3,8 | -3,1 | -2,1 |
| hypothetical protein | WR30_RS29710 | Bcep18194_C7367 | 17,6 | -1,1 | 18,9 |
| histidinol-phosphate aminotransferase | WR30_RS19745 | Bcep18194_C7445 | -1,5 | -3,7 | -3,8 |
| porin | WR30_RS19805 | Bcep18194_C7455 | 1,3 | 5,4 | 2,4 |
| TetR family transcriptional regulator | WR30_RS19870 | Bcep18194_C7473 | 3,1 | 2,2 | 1,8 |
| Beta-galactosidase/beta- glucuronidase family protein | WR30_RS20085 | Bcep18194_C7540 | 1,1 | -4,4 | -38,3 |
| Alpha-1,2-mannosidase, putative | WR30_RS20090 | Bcep18194_C7541 | 1,0 | -6,2 | -46,9 |
| thioesterase superfamily protein | WR30_RS12165 | DM41_3182 | 7,5 | 4,4 | 3,8 |
| phage Gp37/Gp68 family protein | WR30_RS10955 | DM42_3324 | -2,4 | -3,6 | -3,3 |
| ATP synthase family protein | WR30_RS30665 | DM80_5962 | 13,9 | 3,1 | 38,1 |
| ATP synthase F1, alpha subunit AtpA | WR30_RS30670 | DM80_5963 | 64,4 | 5,8 | 51,6 |
| ATP synthase delta (OSCP) subunit | WR30_RS30675 | DM80_5964 | 97,7 | 8,6 | 89,3 |
| ATP synthase F0, C subunit AtpE | WR30_RS30680 | DM80_5965 | 85,0 | 6,0 | 42,2 |
| ATP_synt_6_or_A: ATP synthase F0, A subunit AtpB | WR30_RS30685 | DM80_5966 | 141,0 | 10,9 | 79,9 |
| N-ATPase, AtpR subunit | WR30_RS30690 | DM80_5967 | 14,6 | 5,5 | 14,8 |
| F0F1-ATPase subunit family protein | WR30_RS30695 | DM80_5968 | 37,8 | 11,5 | 38,1 |
| ATP synthase, Delta/Epsilon chain, beta-sandwich domain protein | WR30_RS30700 | DM80_5969 | 30,5 | 5,4 | 44,3 |
| ATP synthase F1, beta subunit AtpD | WR30_RS30705 | DM80_5970 | 174,9 | 14,6 | 76,1 |
| BON domain protein | WR30_RS30710 | DM80_5971 | 200,9 | 6,8 | 160,9 |
| transposase | WR30_RS30505 | GEM_0247 | -9,3 | -12,1 | -7,3 |
| transcriptional regulator, LysR family | WR30_RS25330 | GEM_3858 | 5,6 | 2,0 | 14,8 |
| hypothetical protein | WR30_RS25325 | GEM_3859 | 10,7 | 7,2 | 36,0 |
| putative transglycosylase associated protein | WR30_RS25320 | GEM_3860 | 1,2 | 3,0 | 3,9 |
| putative fimbrial protein | WR30_RS25315 | GEM_3861 | 17,4 | 1,4 | 12,9 |
| Outer membrane usher protein | WR30_RS25310 | GEM_3862 | -1,4 | -7,6 | -2,2 |
| gp58 | WR30_RS26055 | GEM_4034 | 15,8 | 61,0 | 19,0 |
| hypothetical protein | WR30_RS26050 | GEM_4035 | 26,7 | 109,9 | 35,8 |
| gp8 | WR30_RS26045 | GEM_4036 | 27,1 | 129,8 | 32,0 |
| gp4 | WR30_RS26035 | GEM_4039 | 19,8 | 56,1 | 22,0 |
| gp3 | WR30_RS26030 | GEM_4040 | 9,6 | 33,1 | 12,2 |
| hypothetical protein | WR30_RS26025 | GEM_4041 | 15,6 | 50,6 | 8,7 |
| putative terminase (small subunit) | WR30_RS26015 | GEM_4045 | 16,8 | 23,9 | 8,7 |
| putative terminase (large subunit) | WR30_RS26010 | GEM_4046 | 16,4 | 33,1 | 6,7 |
| hypothetical protein | WR30_RS26005 | GEM_4047 | 24,8 | 89,9 | 15,2 |
| putative portal protein | WR30_RS26000 | GEM_4048 | 12,6 | 24,9 | 7,1 |
| phage minor capsid protein C, putative | WR30_RS25995 | GEM_4049 | 14,3 | 41,4 | 11,1 |
| gp6, major capsid head protein | WR30_RS25990 | GEM_4050 | 68,6 | 130,7 | 43,4 |
| gp7, conserved hypothetical protein | WR30_RS25985 | GEM_4051 | 51,6 | 213,8 | 29,4 |
| gp8, conserved hypothetical protein | WR30_RS25980 | GEM_4052 | 58,5 | 196,7 | 49,5 |
| gp9, phage head-tail adaptor, putative | WR30_RS25975 | GEM_4053 | 44,3 | 115,4 | 39,1 |
| hypothetical protein | WR30_RS25970 | GEM_4054 | 34,1 | 108,4 | 24,3 |
| hypothetical protein | WR30_RS25965 | GEM_4055 | 103,3 | 278,2 | 106,2 |
| gp70, conserved hypothetical protein | WR30_RS25960 | GEM_4056 | 85,6 | 198,1 | 73,0 |
| gp69, conserved hypothetical protein | WR30_RS25955 | GEM_4057 | 41,1 | 82,7 | 22,3 |
| gp13, conserved hypothetical protein | WR30_RS25950 | GEM_4058 | 36,0 | 136,2 | 17,8 |
| tail length tape measure protein gp13 | WR30_RS25945 | GEM_4059 | 20,7 | 61,8 | 12,0 |
| minor tail protein gp14 | WR30_RS25940 | GEM_4060 | 30,3 | 55,7 | 14,3 |
| minor tail protein gp16 | WR30_RS25930 | GEM_4062 | 23,9 | 81,0 | 17,1 |
| hypothetical protein | WR30_RS25925 | GEM_4063 | 50,9 | 190,0 | 41,6 |
| tail component protein gp18 | WR30_RS25920 | GEM_4064 | 21,4 | 85,0 | 10,8 |
| tail tip fiber protein gp19 | WR30_RS25915 | GEM_4065 | 10,7 | 23,9 | 12,0 |
| hypothetical protein | WR30_RS25905 | GEM_4067 | 5,7 | 8,3 | 3,2 |
| Glycosyltransferase | WR30_RS21615 | MYA_0151 | 11,6 | 20,0 | 10,3 |
| 4-keto-6-deoxy-N-Acetyl-D-hexosaminyl-(Lipid carrier) aminotransferase | WR30_RS21610 | MYA_0153 | 13,7 | 14,8 | 5,4 |
| SAM (and some other nucleotide) binding motif protein | WR30_RS21605 | MYA_0154 | 26,0 | 23,8 | 11,2 |
| hypothetical protein | WR30_RS21600 | MYA_0155 | 26,5 | 19,2 | 18,4 |
| Glycine oxidase ThiO | WR30_RS21595 | MYA_0156 | 41,9 | 41,1 | 17,3 |
| hypothetical protein | WR30_RS21590 | MYA_0157 | 18,6 | 13,5 | 14,4 |
| TPR domain protein, Putative component of TonB system | WR30_RS21585 | MYA_0159 | 10,9 | 12,8 | 3,4 |
| TPR domain protein, Putative component of TonB system | WR30_RS21575 | MYA_0162 | 10,1 | 12,3 | 10,4 |
| Curculin domain protein (mannose-binding) lectin | WR30_RS07565 | MYA_0783 | 3,0 | 3,4 | 24,9 |
| Arsenical resistance operon trans-acting repressor ArsD | WR30_RS18685 | MYA_4549 | 18,3 | 1,8 | 19,8 |
| Arsenical pump-driving ATPase | WR30_RS18675 | MYA_4550 | 10,0 | 1,5 | 8,9 |
