# Supplementary material for: Understanding the Pathogenicity of Burkholderia contaminans, an Emerging Pathogen in Cystic Fibrosis
Source: PLoS One. 2016 Aug 11;11(8):e0160975. doi: 10.1371/journal.pone.0160975 (PMC4981469; doi:10.1371/journal.pone.0160975)
Supplement: S2 Table — (DOCX) [file pone.0160975.s002.docx]

| **Gene product** | **Gene symbol** | **Accession No: *B. contaminans* FFH2055** | **Accession No: *B. cenocepacia* J2315** | **Fold change of expression (MF16_B/467_S)** | | |
| --- | --- | --- | --- | --- | --- | --- |
|  |  |  |  | **Serum** | **Sputum** | **BSM** |
| ***BDSF synthesis*** |  |  |  |  |  |  |
| Cis-2-dodecenoic acid receptor | RpfR | WR30_RS32185 | BCAM0580 | 2.5 | 2.0 | 3.4 |
| bifunctional crotonase homologue | RpfF | WR30_RS32190 | BCAM0581 | 2.7 | 1.3 | 3.5 |
| ***N-AHL synthesis*** |  |  |  |  |  |  |
| N-acylhomoserine lactone synthase | CepI | WR30_RS25850 | BCAM1870 | 7.8 | 2.0 | 1.3 |
| conserved hypothetical protein |  | WR30_RS25855 | BCAM1871 | 6.4 | 1.9 | 1.6 |
| ***Extracellular proteases*** |  |  |  |  |  |  |
| zinc metalloprotease | ZmpA | WR30_RS18980 | BCAS0409 | 3.0 | 8.9 | 1.5 |
| zinc metalloprotease | ZmpB | WR30_RS33500 | BCAM2307 | 45.3 | 64.4 | 48.8 |
| ***Ornibactin*** |  |  |  |  |  |  |
| ornibactin biosynthesis non-ribosomal peptide synthase | OrbI | WR30_RS11705 | BCAL1696 | -1.3 | 4.4 | 1.2 |
| ornibactin biosynthesis non-ribosomal peptide synthase | OrbJ | WR30_RS11710 | BCAL1697 | 1.7 | 3.9 | 1.4 |
| ornibactin biosynthesis protein | OrbK | WR30_RS11715 | BCAL1698 | 4.3 | 2.1 | -1.3 |
| putative L-ornithine 5-monooxygenase | PvdA | WR30_RS11720 | BCAL1699 | -1.2 | 5.5 | 6.1 |
| ornibactin receptor | OrbA | WR30_RS11725 | BCAL1700 | -1.5 | 4.6 | 3.4 |
| ornibactin synthetase F | OrbF | WR30_RS11730 | BCAL1701 | -1.9 | 4.4 | 2.8 |
| ***Lectins*** |  |  |  |  |  |  |
| lectin | BclC | WR30_RS30310 | BCAM0185 | 1.8 | 12.8 | 4.4 |
| lectin | BclA | WR30_RS30315 | BCAM0186 | 6.3 | 7.2 | 4.1 |
| ***Nematocidal protein*** |  |  |  |  |  |  |
| nematocidal protein | AidA | WR30_RS19200 | BCAS0293 | 41.9 | 45.3 | 20.5 |
| ***Flp-type pili*** |  |  |  |  |  |  |
| Flp/Fap pilin component |  | WR30_RS10640 | BCAL1525 | 22.3 | 13.7 | 24.8 |
| peptidase A24A, prepilin type IV |  | WR30_RS10645 | BCAL1525a | 17.1 | 8.4 | 17.5 |
| TadE family protein |  | WR30_RS10650 | BCAL1526 | 23.6 | 12.7 | 30.5 |
| Flp pilus assembly protein CpaB family |  | WR30_RS10655 | BCAL1527 | 17.8 | 17.9 | 28.2 |
| type II and III secretion system protein |  | WR30_RS10660 | BCAL1528 | 18.4 | 10.3 | 22.3 |
| response regulator receiver protein |  | WR30_RS10665 | BCAL1529 | 7.0 | 6.5 | 10.9 |
| type II secretion system protein E |  | WR30_RS10670 | BCAL1530 | 4.2 | 4.9 | 11.2 |
| type II secretion system protein |  | WR30_RS10675 | BCAL1531 | 4.4 | 2.4 | 5.1 |
| type II secretion system protein |  | WR30_RS10680 | BCAL1532 | 4.4 | 4.7 | 7.4 |
| tetratricopeptide TPR_2 repeat protein |  | WR30_RS10685 | BCAL1533 | 4.3 | 5.8 | 5.1 |
| FAD linked oxidase domain protein |  | WR30_RS10690 | BCAL1534 | 12.6 | 6.7 | 14.7 |
| membrane protein |  | WR30_RS10695 | BCAL1535 | 3.2 | 4.9 | 10.3 |
| ***T3SS*** |  |  |  |  |  |  |
| hypothetical protein |  | WR30_RS26925 | BCAM2052 | 7.1 | 3.9 | -1.3 |
| hypothetical protein |  | WR30_RS26930 | BCAM2053 | 23.8 | 14.6 | 1.2 |
| type III secretion system protein | BcsC | WR30_RS26940 | BCAM2055 | 4.4 | 1.7 | -2.8 |
| type III secretion system protein | BscS | WR30_RS26945 | BCAM2056 | 6.6 | 7.2 | -1.9 |
| type III secretion system protein | BscV | WR30_RS26950 | BCAM2057 | 6.3 | 5.5 | -2.1 |
| ***T6SS*** |  |  |  |  |  |  |
| putative type VI secretion system protein | TssC | WR30_RS05580 | BCAL0342 | 5.1 | 3.6 | 3.7 |
| putative type VI secretion system protein | TssD | WR30_RS05585 | BCAL0343 | 4.1 | 2.8 | 4.3 |

**detected mutations in isolate 467_S:**

BLUE - missense
