# Supplementary material for: Understanding the Pathogenicity of Burkholderia contaminans, an Emerging Pathogen in Cystic Fibrosis
Source: PLoS One. 2016 Aug 11;11(8):e0160975. doi: 10.1371/journal.pone.0160975 (PMC4981469; doi:10.1371/journal.pone.0160975)
Supplement: S3 Table — (DOCX) [file pone.0160975.s003.docx]

| **Gene product** | **Gene symbol** | **Accession No: *B. contaminans* FFH2055** | **Accession No: *B. cenocepacia* J2315** | **Fold change of expression (MF16_B/467_S)** | | |
| --- | --- | --- | --- | --- | --- | --- |
|  |  |  |  | **Serum** | **Sputum** | **BSM** |
| B-type flagellar hook-associated protein 2 | FliD1 | WR30_RS21565 | BCAL0113 | 3.2 | 11.7 | 50.9 |
| flagellin | FliC | WR30_RS21560 | BCAL0114 | 12.9 | 19.8 | 146.0 |
| transcriptional activator | FlhD | WR30_RS21520 | BCAL0124 | 21.1 | 2.7 | 2.9 |
| transcriptional activator | FlhC | WR30_RS21515 | BCAL0125 | 17.9 | 3.2 | 3.7 |
| flagellar motor protein | MotA | WR30_RS21510 | BCAL0126 | 30.1 | 8.2 | 13.6 |
| flagellar motor protein | MotB | WR30_RS21505 | BCAL0127 | 25.8 | 7.2 | 27.3 |
| chemotaxis two-component response regulator | CheY | WR30_RS21500 | BCAL0128 | 4.3 | 5.1 | 3.6 |
| chemotaxis two-component sensor kinase | CheA | WR30_RS21495 | BCAL0129 | 7.6 | 6.1 | 13.8 |
| chemotaxis protein | CheW | WR30_RS21490 | BCAL0130 | 9.3 | 4.9 | 18.1 |
| methyl-accepting chemotaxis protein | Tar | WR30_RS21485 | BCAL0131 | 14.3 | 8.6 | 11.9 |
| chemotaxis protein methyltransferase | CheR | WR30_RS21480 | BCAL0132 | 11.6 | 9.2 | 21.3 |
| chemoreceptor glutamine deamidase | CheD | WR30_RS21475 | BCAL0133 | 4.1 | 2.5 | 18.3 |
| chemotaxis-specific methylesterase | CheB1 | WR30_RS21470 | BCAL0134 | 9.6 | 4.7 | 17.8 |
| chemotaxis protein | CheY | WR30_RS21465 | BCAL0135 | 1.7 | 2.1 | 5.9 |
| flagellar biosynthesis protein | FlhB | WR30_RS21430 | BCAL0140 | 39.1 | 48.8 | 11.8 |
| flagellar biosynthesis protein | FlhA | WR30_RS21425 | BCAL0141 | 21.4 | 31.6 | 9.7 |
| flagellar biosynthesis regulator | FlhF | WR30_RS21420 | BCAL0142 | 18.9 | 26.7 | 19.4 |
| putative flagellar biosynthesis protein |  | WR30_RS21415 | BCAL0143 | 28.6 | 8.8 | 7.2 |
| flagellar biosynthesis sigma factor | FliA | WR30_RS21410 | BCAL0144 | 12.9 | 22.2 | 4.6 |
| putative flagellar hook-length control protein | FliK | WR30_RS00685 | BCAL0520 | 47.2 | 16.0 | 8.9 |
| flagellum-specific ATP synthase | FliI | WR30_RS00695 | BCAL0522 | 30.1 | 17.1 | 10.7 |
| flagellar assembly protein H | FliH | WR30_RS00700 | BCAL0523 | 57.3 | 1.2 | 1.4 |
| flagellar motor switch protein G | FliG | WR30_RS00705 | BCAL0524 | 44.9 | 34.1 | 14.5 |
| flagellar MS-ring protein | FliF | WR30_RS00710 | BCAL0525 | 23.9 | 27.1 | 7.6 |
| flagellar hook-basal body complex protein | FliE | WR30_RS00715 | BCAL0526 | 11.2 | 12.0 | 4.9 |
| flagellar protein | FliS | WR30_RS00720 | BCAL0527 | 11.0 | 11.1 | 19.6 |
| hypothetical protein |  | WR30_RS00725 | BCAL0528 | 35.3 | 3.3 | 4.0 |
| hypothetical protein |  | WR30_RS00730 | BCAL0529 | 10.9 | 1.9 | 9.2 |
| putative export system protein |  | WR30_RS00735 | BCAL0530 | 3.9 | 1.3 | 7.9 |
| flagella synthesis protein | FlgN | WR30_RS00905 | BCAL0561 | 12.5 | 13.5 | 86.2 |
| flagellin synthesis anti-sigma-28 factor | FlgM | WR30_RS00910 | BCAL0562 | 55.3 | 68.6 | 26.4 |
| flagellar basal body P-ring biosynthesis protein | FlgA | WR30_RS00915 | BCAL0563 | 36.8 | 24.6 | 6.5 |
| flagellar basal body rod protein | FlgB | WR30_RS00920 | BCAL0564 | 60.1 | 25.1 | 24.1 |
| flagellar basal body rod protein | FlgC | WR30_RS00925 | BCAL0565 | 55.7 | 27.1 | 20.4 |
| flagellar basal body rod modification protein | FlgD | WR30_RS00930 | BCAL0566 | 36.8 | 33.6 | 20.3 |
| flagellar hook protein | FlgE | WR30_RS00935 | BCAL0567 | 31.6 | 20.5 | 15.9 |
| flagellar basal body rod protein | FlgF | WR30_RS00940 | BCAL0568 | 35.8 | 27.1 | 21.6 |
| flagellar basal body rod protein | FlgG | WR30_RS00945 | BCAL0569 | 26.7 | 30.3 | 18.3 |
| flagellar basal body L-ring protein | FlgH | WR30_RS00950 | BCAL0570 | 33.8 | 44.0 | 16.1 |
| flagellar basal body P-ring protein | FlgI | WR30_RS00955 | BCAL0571 | 67.6 | 41.6 | 6.4 |
| flagellar rod assembly protein/muramidase | FlgJ | WR30_RS00960 | BCAL0572 | 21.4 | 16.6 | 11.1 |
| YcgR family protein |  | WR30_RS00965 | BCAL0575 | 2.2 | 1.9 | 6.8 |
| flagellar hook-associated protein | FlgK | WR30_RS00970 | BCAL0576 | 19.0 | 12.4 | 88.6 |
| flagellar hook-associated protein | FlgL | WR30_RS00975 | BCAL0577 | 39.1 | 51.6 | 212.3 |
| putative methyl-accepting chemotaxis protein |  | WR30_RS01865 | BCAL0762 | 9.8 | 13.5 | 64.4 |
| putative methyl-accepting chemotaxis protein |  | WR30_RS10290 | BCAL1452 | 2.2 | 13.0 | 14.7 |
| flagellar biosynthetic protein | FliR | WR30_RS36225 | BCAL3501 | 8.9 | 26.9 | 3.8 |
| flagellar biosynthesis protein | FliQ | WR30_RS36230 | BCAL3502 | 15.3 | 1.9 | 1.1 |
| flagellar biosynthesis protein | FliP | WR30_RS36235 | BCAL3503 | 10.6 | 14.0 | 10.8 |
| flagellar protein | FliO | WR30_RS36240 | BCAL3504 | 43.1 | 8.8 | 1.1 |
| flagellar motor switch protein | FliN | WR30_RS36245 | BCAL3505 | 19.3 | 24.3 | 7.5 |
| flagellar motor switch protein | FliM | WR30_RS36250 | BCAL3506 | 27.7 | 37.3 | 13.9 |
| flagellar basal body-associated protein | FliL | WR30_RS36255 | BCAL3507 | 45.9 | 35.3 | 19.0 |
| chemotaxis protein | PomA | WR30_RS33125 | BCAM0777 | 15.3 | 8.6 | 72.0 |
| OmpA family protein |  | WR30_RS33130 | BCAM0778 | 16.7 | 3.6 | 80.4 |
| putative methyl-accepting chemotaxis protein |  | WR30_RS33135 | BCAM0779 | 1.9 | 3.1 | 9.6 |
| flagellar hook protein | FlgE | WR30_RS35370 | BCAM0987 | -2.1 | 11.1 | 6.0 |
| methyl-accepting chemotaxis protein |  | WR30_RS23080 | BCAM1424 | 8.8 | 9.9 | 50.9 |
| putative methyl-accepting chemotaxis protein |  | WR30_RS23490 | BCAM1503 | 2.0 | 3.9 | 11.2 |
| methyl-accepting chemotaxis protein |  | WR30_RS24155 | BCAM1572 | 10.8 | 4.3 | 59.3 |
| methyl-accepting chemotaxis protein |  | WR30_RS25520 | BCAM1804 | 43.7 | 12.6 | 87.4 |
| methyl-accepting chemotaxis protein |  | WR30_RS26785 | BCAM2021 | 1.9 | 5.4 | 34.1 |
| putative methyl-accepting chemotaxis protein |  | WR30_RS33935 | BCAM2374 | 1.4 | 10.1 | 33.8 |
| methyl-accepting chemotaxis protein | Tsr | WR30_RS15365 | BCAM2563 | 22.0 | 6.5 | 37.0 |
| putative methyl-accepting chemotaxis protein | Tar | WR30_RS28560 | BCAM2689 | -1.1 | -1.1 | 4.6 |
| A-type flagellar hook-associated protein 2 | FliD2 | WR30_RS20405 | BCAS0104 | -17.1 | -1.4 | 1.7 |
| putative methyl-accepting chemotaxis protein |  | WR30_RS19935 | BCAS0181 | 2.2 | 3.1 | 6.3 |
| flagellar protein | FliT | WR30_RS21570 | x | 4.7 | 12.6 | 82.7 |
| flagellar motor component-like protein |  | WR30_RS31200 | x | 2.7 | 3.6 | 23.9 |
| outer membrane protein, OmpA/MotB family |  | WR30_RS31195 | x | 5.9 | 6.1 | 24.4 |
| methyl-accepting chemotaxis sensory transducer |  | WR30_RS20930 | x | 1.7 | 2.8 | 10.1 |
| methyl-accepting chemotaxis (MCP) signaling domain protein |  | WR30_RS18510 | x | -9.0 | -1.7 | -1.9 |

**detected mutations in isolate 467_S:**

BLUE - missense

YELLOW - frameshift
