# Supplementary material for: Understanding the Pathogenicity of Burkholderia contaminans, an Emerging Pathogen in Cystic Fibrosis
Source: PLoS One. 2016 Aug 11;11(8):e0160975. doi: 10.1371/journal.pone.0160975 (PMC4981469; doi:10.1371/journal.pone.0160975)
Supplement: S4 Table — (DOCX) [file pone.0160975.s004.docx]

| **Gene product** | **Gene symbol** | **Accession No: *B. contaminans* FFH2055** | **Fold change of expression (MF16_B/467_S)** | | |
| --- | --- | --- | --- | --- | --- |
|  |  |  | **Serum** | **Sputum** | **BSM** |
| ***Pyrrolnitrin*** |  |  |  |  |  |
| tryptophan halogenase | PrnA | WR30_RS16030 | 5.9 | 13.2 | 21.0 |
| hypothetical protein | PrnB | WR30_RS16035 | 8.6 | 18.6 | 20.3 |
| FAD dependent oxidoreductase | PrnC | WR30_RS16040 | 3.0 | 8.1 | 12.3 |
| Rieske (2Fe-2S) protein | PrnD | WR30_RS16045 | 3.9 | 36.5 | 15.3 |
| ***Occidiofungin*** |  |  |  |  |  |
| putative FAD linked oxidase domain protein |  | WR30_RS20035 | 3.5 | 11.9 | 4.5 |
| putative LuxR-type regulator | AmbR1 | WR30_RS20030 | 3.7 | 15.1 | 2.3 |
| putative LuxR-type regulator | AmbR2 | WR30_RS20025 | 6.3 | 29.0 | 2.7 |
| putative cyclic peptide transporter | OcfA | WR30_RS20020 | 4.4 | 17.8 | 6.1 |
| hypothetical protein | OcfB | WR30_RS20015 | 2.5 | 20.7 | 9.8 |
| putative glycosyl transferase | OcfC | WR30_RS20010 | 6.1 | 67.6 | 46.2 |
| putative nonribosomal peptide synthetase | OcfD | WR30_RS20005 | 14.5 | 120.3 | 21.1 |
| putative nonribosomal peptide synthetase | OcfE | WR30_RS20000 | 8.5 | 67.2 | 17.0 |
| putative nonribosomal peptide synthetase | OcfF | WR30_RS19995 | 9.2 | 124.5 | 8.8 |
| putative beta-lactamase domain protein | OcfG | WR30_RS19990 | 14.1 | 99.0 | 16.4 |
| putative beta-ketoacyl synthase nonribosomal peptide synthetase | OcfH | WR30_RS19985 | 12.9 | 131.6 | 22.3 |
| putative short chain dehydrogenase/reductase SDR | OcfI | WR30_RS19980 | 10.3 | 38.1 | 6.1 |
| putative beta-ketoacyl synthetase | OcfJ | WR30_RS19975 | 10.6 | 50.9 | -1.7 |
| putative taurine catabolism dioxygenase | OcfK | WR30_RS19970 | 4.4 | 23.6 | 3.6 |
| putative transaminase | OcfL | WR30_RS19965 | 3.8 | 20.0 | 2.9 |
| putative epimerase/dehydratase | OcfM | WR30_RS19960 | 3.2 | 7.9 | 2.1 |
| putative thioesterase | OcfN | WR30_RS19955 | 3.1 | 10.2 | 1.8 |
| hypothetical protein |  | WR30_RS19950 | 1.2 | 2.4 | 2.1 |

¨

**detected mutations in isolate 467_S:**

BLUE - missense

YELLOW - frameshift
